# Supplementary material for: Exploration of recovery of people living with severe mental illness (SMI) in low-income and middle-income countries (LMIC): a scoping review protocol
Source: BMJ Open. 2020 Feb 3;10(2):e032912. doi: 10.1136/bmjopen-2019-032912 (PMC7044907; doi:10.1136/bmjopen-2019-032912)
Supplement: Author's manuscript [file bmjopen-2019-032912.draft_revisions.pdf]

BMJ Open

BMJ Open is committed to open peer review. As part of this commitment we make the peer review history of every article we publish publicly available.

When an article is published we post the peer reviewers' comments and the authors' responses online. We also post the versions of the paper that were used during peer review. These are the versions that the peer review comments apply to.

The versions of the paper that follow are the versions that were submitted during the peer review process. They are not the versions of record or the final published versions. They should not be cited or distributed as the published version of this manuscript.

BMJ Open is an open access journal and the full, final, typeset and author-corrected version of record of the manuscript is available on our site with no access controls, subscription charges or pay-per-view fees (http://bmjopen.bmj.com).

If you have any questions on BMJ Open's open peer review process please email info.bmjopen@bmj.com

BMJ Open

An exploration of recovery of people living with severe mental illness (SMI) in low- and- middle -income countries (LMIC): a scoping review protocol

Journal:	*BMJ Open*
Manuscript ID	bmjopen-2019-032912
Article Type:	Protocol
Date Submitted by the Author:	11-Jul-2019

Complete List of Authors:	Gamieldien, Fadia; University of Cape Town, Psychiatry; University of Cape Town, Health and Rehabilitation Sciences<br>Galvaan, Roshan ; University of Cape Town, Health and Rehabilitation Sciences<br>Myers, Bronwyn; South African Medical Research Council, Alcohol and Drug Abuse Research Unit<br>Sorsdahl, Katherine; University of Cape Town, Psychiatry

Keywords:	recovery, severe mental illness, scoping review, low-and-middle- income countries

SCHOLARONE™ Manuscripts

I, the Submitting Author has the right to grant and does grant on behalf of all authors of the Work (as defined in the below author licence), an exclusive licence and/or a non-exclusive licence for contributions from authors who are: i) UK Crown employees; ii) where BMJ has agreed a CC-BY licence shall apply, and/or iii) in accordance with the terms applicable for US Federal Government officers or employees acting as part of their official duties; on a worldwide, perpetual, irrevocable, royalty-free basis to BMJ Publishing Group Ltd ("BMJ") its licensees and where the relevant Journal is co-owned by BMJ to the co-owners of the Journal, to publish the Work in this journal and any other BMJ products and to exploit all rights, as set out in our licence.

The Submitting Author accepts and understands that any supply made under these terms is made by BMJ to the Submitting Author unless you are acting as an employee on behalf of your employer or a postgraduate student of an affiliated institution which is paying any applicable article publishing charge ("APC") for Open Access articles. Where the Submitting Author wishes to make the Work available on an Open Access basis (and intends to pay the relevant APC), the terms of reuse of such Open Access shall be governed by a Creative Commons licence – details of these licences and which Creative Commons licence will apply to this Work are set out in our licence referred to above.

Other than as permitted in any relevant BMJ Author's Self Archiving Policies, I confirm this Work has not been accepted for publication elsewhere, is not being considered for publication elsewhere and does not duplicate material already published. I confirm all authors consent to publication of this Work and authorise the granting of this licence.

For peer review only

An exploration of recovery of people living with severe mental illness (SMI) in low- and- middle -income countries (LMIC): a scoping review protocol

Fadia Gamieldien[1], *Roshan Galvaan[2], Bronwyn Myers,[3]* ***Katherine Sorsdahl [1]***

[1] Alan J. Flisher Centre for Public Mental Health, Department of Psychiatry and Mental Health, University of Cape Town, Cape Town, South Africa

2. Division of Occupational Therapy, Department of Health and Rehabilitation Sciences, University of Cape Town, Cape Town, South Africa

3 Alcohol, Tobacco and Other Drug Research Unit, South African Medical Research Council and Division of Addiction Psychiatry, Department of Psychiatry and Mental Health, University of Cape Town,Cape Town, South Africa

Corresponding Author: Ms Fadia Gamieldien

Orcid number 0000-0003-2820-6484

Address: Alan J Flisher Centre for Public Mental Health

Department of Psychiatry and Mental Health

University of Cape Town

46 Sawkins Road

Rondebosch, 7700

Cape Town

South Africa

Fadia.Gamieldien@uct.ac.za

+27 82 974 7468

Keywords

Recovery, severe mental illness, low-and-middle-income countries, scoping review

For peer review only - http://bmjopen.bmj.com/site/about/guidelines.xhtml

ABSTRACT

Introduction: The construct of recovery was conceptualised in high income countries and its applicability in low-and- middle- income countries is underexplored. A scoping review is proposed to synthesise knowledge, review conceptual overlap and map key elements of recovery from severe mental illness in low-and- middle- income countries. We aim to appraise the literature so as to inform future recovery-oriented services that consider the cultural and contextual influences on recovery from severe mental illness.

Methods and analysis: The following electronic databases: MEDLINE via PubMed, SCOPUS (which included contents of Embase), PsycINFO, CINAHL, Africa-Wide Information, PSYCHarticles, Health source: Nursing/Academic Edition, Academic Search Premier and SocINDEX all via the EBSCOHOST platform, the Latin American and Caribbean Health Sciences Literature (LILACS), the Cochrane Centre Register of Controlled Trials) and grey literature sources will be searched to identify studies appropriate for inclusion. Eligible studies will be independently screened for inclusion and exclusion by two reviewers using a checklist developed for this purpose. Studies published between 1993 and 2019 that focuses on recovery from severe mental illness in a low-and -middle- income country will be included. Findings will be compared, and discrepancies will be discussed. Unresolved discrepancies will be referred to a third reviewer. All bibliographic data and study characteristics will be extracted and thematically analysed using a tool developed through an iterative process by the research team. Indicators will be classified according to a predefined conceptual framework and categorised and described using qualitative content analysis.

Ethics and dissemination: This scoping review aims at synthesising information from available publications hence it does not require ethical approval. The results will be disseminated through a peer-reviewed publication, conference presentations and future workshops with stakeholders involved within the recovery paradigm of mental health policy and practice. We have registered our scoping review title with the Joanna Briggs Institute.

For peer review only

Strengths and limitations of the study

- This scoping review allows for the systematic synthesis of knowledge on recovery from severe mental illness in to low- and middle-income countries in a rigorous and methodological manner
- This review will highlight the diversity of severe mental illness since there is no agreed upon criteria for including mental illnesses into this category
- Knowledge gaps related to recovery as a multidimensional construct in low- and middle-income countries will be identified
- Only conceptual and theoretical underpinnings of recovery as a construct in LMIC will be discussed without a focus on intervention outcomes.
- Data synthesis will be limited to full text articles available in English only published between 1993 and 2019

For peer review only

Word count: 3310 (excludes abstract, strengths and limitations, table and references)

For peer review only - http://bmjopen.bmj.com/site/about/guidelines.xhtml

INTRODUCTION

Mental, neurological and substance use (MNS) disorders contribute significantly to the global burden of disease. Whiteford, Ferrari, Degenhardt, Feigin, and Vos (2015) report that MNS disorders account for 10% of disability-adjusted life years (DALYs). However, it has been argued that the true global burden of mental health problems may be underestimated by more than a third and that DALYs are closer to 13%[1]. The lifetime prevalence of severe mental illness (SMIs) ranges between 1% and 4%[2]. Although this is relatively low in comparison to common mental disorders (CMDs), SMIs are more disabling and require complex, long-term interventions for the person and their family[1]. Given the move towards deinstitutionalisation of people with psychiatric disorders, shorter hospital stays are resulting in premature discharge rates and repeated relapse, creating a revolving door phenomenon[3]. These frequent hospital readmissions are costly to the health care system[4].

In low- and- middle- income countries (LMICs) many people living with a SMI are not detected and as a result do not receive the treatment they need[2, 5, 6]. In a service utilisation review of World Health Organisation member countries, it was found that the lifetime prevalence of mental disorders amongst adults was between 12% to 49%[5]. The review found the treatment gap to be wide across the psychiatric disorders examined. The treatment gap for schizophrenia and non-affective psychosis was 32%. The gap for other disorders was: major depressive disorder, 56%; bipolar disorder, 50%; and alcohol abuse and dependence, 78% [5]. While present in all countries, this treatment gap is greatest in LMICs where there is a need to scale up mental health services[7].

A systematic review on the scaling up of mental health services in LMICs found that in many countries the majority of resources for mental health care are rendered on an inpatient or outpatient basis at large psychiatric hospitals, with little provision of services at primary healthcare facilities or via integration between hospitals and the community[7]. Despite the drive for deinstitutionalisation and for the rendering of services at decentralised locations, in many countries most of the mental health care budget continues to be allocated to large psychiatric hospitals. In these hospitals, the treatment approach is primarily through medication to alleviate acute clinical symptoms, giving little to no attention to the psychosocial needs of service users[3, 7, 8]. Unfortunately the sole focus on clinical recovery and the limited availability of interventions that focus on rehabilitation and personal recovery further exacerbates the revolving door phenomenon and ongoing treatment gap[3, 9].

Recovery oriented approaches offer an extension to medical models of intervention, as they are not solely focused on symptomatic improvement as a marker of clinical recovery[10]. Personal recovery for people with SMI is conceptually distinct from the medical definition of

For peer review only - http://bmjopen.bmj.com/site/about/guidelines.xhtml

recovery which is clinical in nature and mostly values symptom remission[11, 12]. Personal recovery proposes that a person develops new meaning and purpose in their life over the longitudinal course of their illness[10, 13]. Psychiatric rehabilitation has highlighted the need to move beyond treating the symptoms of the illness, to treating its functional and disabling consequences as well [14]. Deegan, a mental health professional and person living with schizophrenia, suggested that mental health service users do not "get rehabilitated" by others, but instead they are active participants in their journey to recover new and valued personal meaning and purpose[15].

The recovery model was initiated in high income countries (HICs) with the mental health consumer and survivor movement at a time when having a diagnosis of SMI was highly stigmatised and the prognosis was poor[15]. The idea that people with an SMI cannot recover was challenged by people living with schizophrenia who were leading meaningful lives in their communities after deinstutionalisation, highlighting that recovery was not dependant on long term psychiatric treatment[16, 17]. For a person living with an SMI, receiving a diagnosis and psychiatric treatment focussed on clinical recovery is a necessary step towards recovery, but personal recovery is also critical given the cyclical, non-linear, long term course of the illness. In Anthony's seminal work[14], he defined recovery as:

"*a deeply personal, unique process of changing ones' attitude, values, feelings, goals, skills and/or roles. It is a way of living a satisfying, hopeful and contributing life even within the limitations caused by illness"*

Research exploring the meaning of recovery among people living with SMI[18-20] found that recovery means different things to different people, supporting a view that there is no definition of recovery that applies to everyone. A literature review conducted in 2018 explored the meaning of recovery amongst people living with schizophrenia[18]. This review included 17 studies explaining the subjective meaning of recovery. A majority of the included studies were qualitative, with a few taking a quantitative approach to exploring the meaning of recovery. Of the 17 papers only four studies were from LMICs (India (n=2), China and South Africa). The results revealed that recovery is complex, non-linear and perceived as both a process and an outcome. The process was viewed as being individual and it was recommended that more consumer perspectives to inform recovery-oriented interventions which are aligned to individuals' personal goals and aspirations were needed. The complexity embedded in viewing recovery as a process and an outcome with multidimensional indicators has been documented by others[21-23]. Further to this, it was recommended that clinicians, caregivers and researchers explore more qualitative and personal narratives of recovery, the tools needed to measure recovery and the development of community-based recovery- oriented services[18].

For peer review only - http://bmjopen.bmj.com/site/about/guidelines.xhtml

The aforementioned review was limited to recovery in people living with Schizophrenia and only included four studies from LMIC countries. Conducting a scoping review focusing specifically on studies conducted in LMICs will contribute to the literature on recovery in these specific contexts and will be useful for the following reasons. First, little attention has been given to describing recovery as a personal and individual journey from LMIC perspectives. As recovery for the person with an SMI takes place in a social context through a range of relationships, more understanding about how contexts can help or hinder recovery is needed[24].

Second, individual meaning-making within recovery is influenced by social, political, economic and human rights factors but further research is needed to understand the diversity of recovery narratives from the perspectives of different population groups [25]. A systematic review and narrative analysis synthesising descriptions and models of personal recovery was undertaken to inform the development of a conceptual framework to guide recovery oriented research and practice[9]. Studies conducted in HIC specifically the United States featured predominantly in the review which included 97 papers. The review culminated in the emergence of a conceptual framework to describe the characteristics of recovery, the processes of recovery and the stages of recovery. The authors reported a difference in studies which included ethnic minorities and where more emphasis was placed on stigma, spirituality, culture and the collectivist nature of recovery. They acknowledged that this area is underexplored[9].

Third, in many African cultures an individual's personhood is manifested through their agency in relation to spirituality, society and self[26]. This may impact on the person and how they experience mental illness and recovery. Understanding cultural concepts and the dynamics of interconnectedness means recognising that personhood in LMICs is weaved into how people experience health, illness and recovery[26]. Considering the person as a spiritual and social being provides opportunities to collaborate with caregivers and communities in developing appropriate interventions[26]. In countries that are spiritually diverse, multicultural and home to people of different religious backgrounds it is important to contextualise mental illness and explore health professionals own perspectives on culture, psychiatry and mental health and where mental illness must be contextualised[27, 28]. Given the reasons cited previously, further exploration into the recovery journeys of people living with SMI in LMICs and how they view the dimensions of recovery is warranted[18].

A scoping review is proposed as a means of synthesising knowledge on a broadly defined topic and systematically mapping key concepts, theories, evidence and research gaps in the topic area, while still being rigorous and methodological in its approach[29, 30]. While systematic reviews and meta-analyses limit their parameters to research trials and quantitative data

For peer review only - http://bmjopen.bmj.com/site/about/guidelines.xhtml

synthesis, scoping reviews adopt a broader approach to the narrative integration of evidence[31]. We aim to appraise the definitions of recovery and its current utilisation for people with SMI in LMICs. The review findings could be used to inform future practice applications which considers the cultural and contextual factors impacting on recovery[32] from SMI outside of HICs. This scoping review title is registered with the Joanna Briggs Institute.

METHODS

A scoping review was deemed as the most suitable method to map existing research on recovery from SMI in LMICs because it allows for the topic to be located, examined, summarised and be presented rapidly and systematically. Additionally it could serve as a precursor to a systematic review [33]. The proposed review will be conducted using the Arksey and O'Malley[30] scoping review methodology and will be reported on using the PRISMA guidelines[34] with an understanding that there is much development as scoping reviews gain popularity especially in health-related topics [29, 35].

Arksley and O'Malley [30] offer a methodological framework for conducting a scoping review. Their approach describes six stages: (1) identification of the research question, (2) identification of relevant studies, (3) selection of studies, (4) charting the data, (5) collating, summarising and reporting of results and (6) consultation with stakeholders [30]. The last stage is optional according to Arksley and O'Malley[30] but others have deemed it a necessary step to aide in methodological rigour[36]. Recommendations to enhance the methodology at each stage will be incorporated into this review[29, 34, 36].

Stage 1: Identifying the research question

In order to guide the search strategy an iterative process of discussion amongst authors occurred in order to develop and refine the research question. The question needed to be clear enough to inform the subsequent stages while still reflecting the scope of inquiry[36]. Given that scoping studies are focused on summarizing the breadth of evidence on a given topic the authors refined the exploratory question to ask the following:

What is known about recovery from severe mental illness in low-and-middle-income countries?

This broad question was developed to allow for a comprehensive review of the literature in order to review and appraise the definition of recovery and how it is understood in mental health research and practice in LMICs. While many have written about this in high income countries[20, 37] there is a dearth of such information in LMICs .

For peer review only - http://bmjopen.bmj.com/site/about/guidelines.xhtml

Stage 2: Identifying relevant studies

Given the aim of the study is to identify primary published and unpublished studies which will answer the research question, a comprehensive search strategy was developed to aide this. Two health sciences librarians assisted the first author in the iterative process of developing a search strategy including inclusion and exclusion criteria. Decisions were made about time frames, language, search terms and search strategies. Trial searches were run to assess whether relevant literature could be identified using the proposed strategy. A discussion of the identified databases, search strategy and selection criteria follow.

Databases

Electronic databases will be searched to identify studies published between January 1993-June 2019. This period was selected as it covers roughly 25 years in the recovery movement, starting with Anthony's initial definition of recovery from mental illness[14]. An expert librarian assisted the first author in identifying information sources that are relevant to the research question. The following databases were selected: MEDLINE via PubMed, SCOPUS (which included contents of Embase), PsycINFO, CINAHL, Africa-Wide Information, PSYCH articles, Health source: Nursing/Academic Edition, Academic Search Premier and SocINDEX all via the EBSCOHOST platform. Additionally, the Latin American and Caribbean Health Sciences Literature (LILACS), the Cochrane Centre Register of Controlled Trials. Grey literature sources will be identified to expand the search and enhance the data sources. Additional sources will be located by means of hand searching reference lists of relevant papers, contacting study authors, searching trial registers, and contacting key personnel involved in recovery-focussed programmes in LMICs. In this way peer reviewed and grey literature from the biomedical sciences, allied health sciences, social sciences and other disciplinary fields will be included.

Search Strategy

Two librarians and the first author developed the eligibility criteria for the scoping review. They identified various definitions of severe mental illness as well as recovery to include in this search strategy. A list of preliminary search terms and filtering methods was developed. The main filtering methods related to the date range of 25 years (1993-2018). This was extended to include January- June 2019 so that new publications would not be missed. A preliminary search was conducted to test the search strategy. Thereafter, the strategy was refined to include MESH terms, filters and Boolean operations to comply with searches across different databases. After the initial search, key words and index terms used across the databases were identified and plotted on a table as indicated below (Table 1).

For peer review only - http://bmjopen.bmj.com/site/about/guidelines.xhtml

Table 1 Search strategy terms

Keyword	Alternative
Recovery	Recovery OR recover OR psychosocial rehabilitation OR mental health rehabilitation OR psychiatric rehabilitation OR mental health recovery OR Recovery of function OR Quality of life
Severe mental illness	Severe mental illness OR bipolar OR delusional disorder OR delusional disorders OR major depressive disorder OR major depressive disorders OR schizophrenia OR manic OR manic-depressive OR paranoid disorder OR paranoid disorders OR psychoses OR psychosis OR psychotic disorder OR psychotic disorders OR schizoaffective disorder OR schizoaffective disorders OR Schizophreniform OR serious mental disorder OR serious mental disorders Schizophrenia Spectrum and Other Psychotic Disorders Bipolar and Related Disorders Depressive Disorder
Low- and-middle- income countries	Deprived Countries OR Deprived Population OR Deprived Populations OR Developing Countries OR Developing Country OR Developing Economies OR Developing Economy OR Developing Nation OR Developing Nations OR Developing Population OR Developing Populations OR Developing World OR LAMI Countries OR LAMI Country OR Less Developed Countries OR Less Developed Country OR Less Developed Economies OR Less Developed Nation OR Less Developed Nations OR Less Developed World OR Lesser Developed Countries OR Lesser Developed Nations OR LMIC OR LMICS OR Low GDP OR Low GNP OR Low Gross Domestic OR Low Gross National OR Low Income Countries OR Low Income Country OR Low Income Economies OR Low Income Economy OR Low Income Nations OR Low Income Population OR Low Income Populations OR Lower GDP OR lower gross domestic OR Lower Income Countries OR Lower Income Country OR Lower Income Nations OR Lower Income Population OR Lower Income Populations OR Middle Income Countries OR Middle Income Country OR Middle Income Economies OR Middle Income Nation OR Middle Income Nations OR Middle Income Population OR Middle Income Populations OR Poor Countries OR Poor Country OR Poor Economies OR Poor Economy OR Poor Nation OR Poor Nations OR Poor Population OR Poor Populations OR poor world OR Poorer Countries OR Poorer Economies OR Poorer Economy OR Poorer Nations OR Poorer Population OR Poorer Populations OR Third World OR Transitional Countries OR Transitional Country OR Transitional Economies OR Transitional Economy OR Under Developed Countries OR Under Developed Country OR under developed nations OR Under Developed World OR Under Served Population OR Under Served Populations OR Underdeveloped Countries OR Underdeveloped Country OR underdeveloped economies OR underdeveloped nations OR underdeveloped

For peer review only

	population OR Underdeveloped World OR Underserved Countries OR Underserved Nations OR Underserved Population OR Underserved Populations<br><br>OR<br>Afghanistan OR Albania OR Algeria OR American Samoa OR Angola OR Armenia OR Azerbaijan OR Bangladesh OR Belarus OR Byelarus OR Belorussia OR Belize OR Benin OR Bhutan OR Bolivia OR Bosnia OR Botswana OR Brazil OR Bulgaria OR Burma OR Burkina Faso OR Burundi OR Cabo Verde OR Cape Verde OR Cambodia OR Cameroon OR Central African Republic OR Chad OR China OR Colombia OR Comoros OR Comores OR Comoro OR Congo OR Costa Rica OR Côte d'Ivoire OR Cuba OR Djibouti OR Dominica OR Dominican Republic OR Ecuador OR Egypt OR El Salvador OR Equatorial Guinea OR Eritrea OR Ethiopia OR Fiji OR Gabon OR Gambia OR Gaza OR Georgia OR Georgia Republic OR Ghana OR Grenada OR Grenadines OR Guatemala OR Guinea OR Guinea- Bissau OR Guyana OR Haiti OR Herzegovina OR Hercegovina OR Honduras OR India OR Indonesia OR Iran OR Iraq OR Jamaica OR Jordan OR Kazakhstan OR Kenya OR Kiribati OR Democratic People's Republic of Korea OR Kosovo OR Kyrgyz OR Kirghizia OR Kirghiz OR Kyrgyzstan OR Lao PDR OR Laos OR Lebanon OR Lesotho OR Liberia OR Libya OR Macedonia OR Madagascar OR Malawi OR Malay OR Malaya OR Malaysia OR Maldives OR Mali OR Marshall Islands OR Mauritania OR Mauritius OR Mexico OR Micronesia OR Moldova OR Mongolia OR Montenegro OR Morocco OR Mozambique OR Myanmar OR Namibia OR Nepal OR Nicaragua OR Niger OR Nigeria OR Pakistan OR Palau OR Panama OR Papua New Guinea OR Paraguay OR Peru OR Philippines OR Principe OR Romania OR Ruanda OR Rwanda OR Samoa OR Sao Tome OR Senegal OR Serbia OR Sierra Leone OR Solomon Islands OR Somalia OR South Africa OR South Sudan OR Sri Lanka OR St Lucia OR St Vincent OR Sudan OR Surinam OR Suriname OR Swaziland OR Syria OR Syrian Arab Republic OR Tajikistan OR Tadzhikistan OR Tajikistan OR Tadzhik OR Tanzania OR Thailand OR Timor OR Togo OR Tonga OR Tunisia OR Turkey OR Turkmen OR Turkmenistan OR Tuvalu OR Uganda OR Ukraine OR Uzbek OR Uzbekistan OR Vanuatu OR Venezuela OR Vietnam OR West Bank OR Yemen OR Zambia OR Zimbabwe

Selection criteria

For peer review only - http://bmjopen.bmj.com/site/about/guidelines.xhtml

Articles will be included in the review if they meet the following inclusion and exclusion criteria

Table 2 Inclusion and exclusion criteria

Criteria for inclusion	Criteria for exclusion
• Published between January 1993- March 2019<br>• Qualitative and quantitative empirical (i.e. primary research) study designs<br>• Theoretical literature on the construct of recovery<br>• Studies will be included if they have been conducted in LMICs<br>• Peer reviewed scientific literature and "grey literature" from LMICs (literature found outside of formal academic publishing) will be considered<br>• Literature focussing on the population of interest; that is adults with SMI in LMICs will be included. The diagnosis of SMI includes schizophrenia spectrum disorders, schizoaffective disorders, bipolar affective disorders, major depressive disorder or related psychotic disorders (schizophreniform, delusional disorder, substance induced psychotic disorder or disorders not otherwise specified) as well as those with a comorbid substance use disorder. Studies and reports will be excluded if they do not include the keywords or alternatives as outlined in table 1<br>• Explicit mention of severe mental illness and recovery in LMICS	• The review will be limited to full text articles written in English. However, the authors acknowledge that this practical decision could mean that potentially relevant papers are missed<br>• Conference abstracts will be excluded although they will be reviewed to see if full reports were published<br>• Children will be excluded as the age range for inclusion is 18 years and older<br>• Duplicate articles from the same study will be excluded<br>• Policy documents will be excluded although they will be reviewed to access research in LMICs that they might have referenced<br>• Studies that occurred only in high income countries, without a LMIC focus will be excluded<br>• Studies must be conducted in LMIC and not in HIC with particpants from LMICs

Stage 3: Study selection

For peer review only - http://bmjopen.bmj.com/site/about/guidelines.xhtml

The scoping review guideline for identifying and selecting studies informs article selection and data extraction [30]. Two screening levels are proposed: a title and abstract review and a full-text review. For the first level of screening, two authors (FG, ZS) will independently screen the title and abstracts of all retrieved citations against a few select inclusion criteria. Any articles that seem relevant by either or both reviewers will be included in the full-text review. Next, these two reviewers will independently screen the full text articles to assess whether they meet the inclusion and exclusion criteria. At both levels of screening, inter-rater agreement will be assessed by calculating Cohen's Kappa. Any disagreement on study selection will be resolved by discussion with a third rater, who will be a senior member of the research team.

Using the aforementioned criteria, the number of studies identified and selected for inclusion in the scoping review will be reported on. A narrative description[29] will accompany the search decision flow chart[34]. The flow chart will show the results from the search, the removal of duplicates, study selection, full retrieval numbers, additional searches from reference lists and a final summary of decisions related to the search decision and an explanation of the review decision process[29].

Stage 4: Charting the data

Next, the two reviewers (FG and ZS) will independently extract data from the identified articles and chart it[30]. A draft data charting formwill be created in the MS Excel programme to facilitate this process[30]. This form will be pre-tested and an iterative process of refining the form will be undertaken as the review commences to ensure that all relevant information is extracted[29].

The form will allow the reviewers to confirm study relevance, record study characteristics and extract information relevant to the review question. The following information will be extracted from the articles: 1) study title; 2) author; 3) study population; 4) participant characteristics; 5) research question; 6) study methodology; 7) study description; 8) intervention type (if applicable); 9) intervention duration (if applicable); 10) outcome measures; 11 summary of findings; 12) definitions of key concepts (recovery and severe mental illness); 13) conceptual links identified; 14) practice implications and; 15) recommendations for further development.

While charting the data it is important to maintain flexibility around including emerging categories and consulting with the team as this occurs. The final version of the charting form along with definitions of included items will be provided as an appendix to the review. This process will facilitate the timeous completion of the review while also maintaining research rigour.

For peer review only

Stage 5: Collating, summarising and reporting the results

All results will be collated, summarised and reported on in order to provide a comprehensive and thorough overview of all the reviewed literature. A three-step approach[36] of analysing the data, reporting results and applying meaning to results will be followed to provide methodological rigour. Collating and summarising results will include a summary of the types of studies conducted (including the quantity, types of study design, populations, setting) as well as a thematic analysis of the results. More specifically, we will code how studies describe the concept of recovery from SMI and how it is defined and understood in LMICs. The framework used to report the findings will be developed iteratively through the examination of results. Additionally, the authors will report on the broader implications of the review in terms of research, policy and practice.

Stage 6: Consultation

While Arksey and O'Malley[30] view the consultation stage as an optional step, Levac[36] views it as a requirement to ensure methodological rigour. During each stage of the review process we will consult with an existing stakeholder advisory committee. This advisory committee consists of the research team and key stakeholders including mental health service providers and service users from within the public health and non-profit organisation (NPO) sectors. This committee has been constituted from the onset of the project and some members are part of a pre-existing group for public mental health specialists. The advisory committee will be consulted to get their views on the relevance of the review, guidance for accessing grey literature and their perspectives on the data extraction process and preliminary findings. Including an advisory committee in the review is informed by a pragmatic approach[38]where the research is treated as a human experience with the understanding that this consultation process is likely to enhance the relevance of this review. It will also support collaborative efforts to facilitate consumer participation, public involvement, access to information and transfer of knowledge[36].

Ethics and dissemination

Since the scoping review methodology aims at synthesising information from available publications, this study does not require ethical approval. Reviews of primary research allows for the mapping of evidence in fields where the body of literature has not yet been comprehensively reviewed or where working definitions and concepts are still developing. The findings will be made available in different formats to facilitate its dissemination. An article reporting on the results will be submitted for publication in a peer -reviewed journal. It will also be presented at relevant conferences and stakeholder engagements involved in mental health policy and practice. These stakeholders include MHSUs, academics, policy makers,

For peer review only

researcher and clinicians. Findings will also be shared via newsletters, policy briefs and social media forums.

Conclusion

This scoping review will cover the breadth of literature examining recovery from SMI in LMICs. We anticipate results which highlight research gaps and a dearth of information on how recovery is described in the literature from perspectives outside of high-income countries. Since we aim to appraise the definitions of recovery and its current utilisation for people with SMI in LMICs, the review findings could be used to inform adaptations to the current understanding of recovery to enhance its applicability to MHSUs in LMICs. This could form the basis for future research and inform new practice directions.

Author Contributions

This protocol was developed by the intellectual contributions of all the authors. All authors were involved in developing the review question and conceptualising the approach. FG developed and tested the search terms in consultation with a subject librarian. FG and KS developed the data extraction guideline, and this was revised based on feedback from the other authors. RG, KS and BM provided substantial critique and review of the draft protocol.

Funding

This work was supported by the DELTAS Africa Initiative [DEL-15-01]. The DELTAS Africa Initiative is an independent funding scheme of the African Academy of Sciences (AAS)'s Alliance for Accelerating Excellence in Science in Africa (AESA) and supported by the New Partnership for Africa's Development Planning and Coordinating Agency (NEPAD Agency) with funding from the Wellcome Trust [DEL-15-01] and the UK government. The views expressed in this publication are those of the author(s) and not necessarily those of AAS, NEPAD Agency, WellcomeTrust or the UK government. BM is funded through the South African Medical Research Council.

For peer review only - http://bmjopen.bmj.com/site/about/guidelines.xhtml

References

1. Vigo D, Thornicroft G, Atun R. Estimating the true global burden of mental illness. The Lancet Psychiatry. 2016;3(2):171-8.
2. Kessler RC, Berglund PA, Bruce ML, Koch JR, Laska EM, Leaf PJ, et al. The prevalence and correlates of untreated serious mental illness. Health Serv Res. 2001;36(6 Pt 1):987.
3. Petersen I, Lund C, Stein DJ. Optimizing mental health services in low-income and middle-income countries. Current opinion in psychiatry. 2011;24(4):318-23.
4. Sfetcu R, Musat S, Haaramo P, Ciutan M, Scintee G, Vladescu C, et al. Overview of post-discharge predictors for psychiatric re-hospitalisations: a systematic review of the literature. BMC Psychiatry. 2017;17(1).
5. Kohn R, Saxena S, Levav I, Saraceno B. The treatment gap in mental health care. World Health Organization Bulletin of the World Health Organization. 2004;82(11):858-66.
6. Demyttenaere K, Bruffaerts R, Posada-Villa J, Gasquet I, Kovess V, Lepine JP, et al. Prevalence, severity, and unmet need for treatment of mental disorders in the World Health Organization World Mental Health Surveys. Jama. 2004;291(21):2581-90.
7. Eaton J, McCay L, Semrau M, Chatterjee S, Baingana F, Araya R, et al. Scale up of services for mental health in low-income and middle-income countries. The Lancet. 2011;378(9802):1592-603.
8. Lund C, Oosthuizen P, Flisher AJ, Emsley R, Stein DJ, Botha U, et al. Pathways to Inpatient Mental Health Care Among People With Schizophrenia Spectrum Disorders in South Africa. Psychiatr Serv. 2010;61(3):235-40.
9. Leamy M, Bird V, Le Boutillier C, Williams J, Slade M. Conceptual framework for personal recovery in mental health: systematic review and narrative synthesis. Br J Psychiatry. 2011;199(6):445-52.
10. Slade M. Personal recovery and mental illness: A guide for mental health professionals: Cambridge University Press; 2009.
11. Slade M. Mental illness and well-being: the central importance of positive psychology and recovery approaches. BMC Health Serv Res. 2010;10:26-.
12. Myers NL, Myers NL. Culture, stress and recovery from schizophrenia: lessons from the field for global mental health. Cult Med Psychiatry. 2010;34(3):500-28.
13. Tew J, Ramon S, Slade M, Bird V, Melton J, Le Boutillier C. Social factors and recovery from mental health difficulties: a review of the evidence. The British Journal of Social Work. 2012;42(3):443-60.
14. Anthony WA. Recovery from mental illness: the guiding vision of the mental health service system in the 1990s. Psychosocial rehabilitation journal. 1993;16(4):11.
15. Deegan PE. Recovery: The lived experience of rehabilitation. Psychosocial rehabilitation journal. 1988;11(4):11.
16. Webb L. The recovery model and complex health needs: what health psychology can learn from mental health and substance misuse service provision. J Health Psychol. 2012;17(5):731-41.
17. Farkas M. The vision of recovery today: what it is and what it means for services. World Psychiatric Association (WPA). 2007;6(2):68.
18. Mathew ST, Nirmala BP, Kommu JVS. Meaning of Recovery Among Persons with Schizophrenia: A Literature Review. Journal of Psychosocial Rehabilitation and Mental Health. 2018;5(1):73-81.
19. Kelly M, Lamont S, Brunero S. An Occupational Perspective of the Recovery Journey in Mental Health. Br J Occup Ther. 2010;73(3):129-35.
20. Cabassa LJ, Nicasio A, Whitley R. Picturing Recovery: A Photovoice Exploration of Recovery Dimensions Among People With Serious Mental Illness. Psychiatric services (Washington, DC). 2013;64(9):10.1176/appi.ps.201200503.

For peer review only

21. Aldersey HM, Adeponle AB, Whitley R. Diverse Approaches to Recovery from Severe Mental Illness. The Palgrave Handbook of Sociocultural Perspectives on Global Mental Health2017. p. 109-27.
22. Frost BG, Tirupati S, Johnston S, Turrell M, Lewin TJ, Sly KA, et al. An Integrated Recovery-oriented Model (IRM) for mental health services: evolution and challenges. BMC Psychiatry. 2017;17:22.
23. Lysaker PH, Hamm JA, Hasson-Ohayon I, Pattison ML, Leonhardt BL. Promoting recovery from severe mental illness: Implications from research on metacognition and metacognitive reflection and insight therapy. World Journal of Psychiatry. 2018;8(1):1-11.
24. Schön U-K, Denhov A, Topor A. Social relationships as a decisive factor in recovering from severe mental illness. Int J Soc Psychiatry. 2009;55(4):336-47.
25. Llewellyn-Beardsley J, Rennick-Egglestone S, Callard F, Crawford P, Farkas M, Hui A, et al. Characteristics of mental health recovery narratives: Systematic review and narrative synthesis.(Research Article). Public Library of Science; 2019. p. e0214678.
26. Kpanake L. Cultural concepts of the person and mental health in Africa. Transcultural Psychiatry. 2018;55(2):198-218.
27. Janse van Rensburg ABR. South African Society of Psychiatrists guidelines for the integration of spirituality in the approach to psychiatric practice : guideline. 2014;20(4):133-9.
28. Swartz L, Foster D. Images of culture and mental illness : South African psychiatric approaches. Social Dynamics. 1984;10(1):17-25.
29. Peters MD, Godfrey CM, Khalil H, McInerney P, Parker D, Soares CB. Guidance for conducting systematic scoping reviews. International journal of evidence-based healthcare. 2015;13(3):141-6.
30. Arksey H, O'Malley L. Scoping studies: towards a methodological framework. International Journal of Social Research Methodology. 2005;8(1):19-32.
31. Dijkers M. What is a scoping review?. KT Update, 4 (1). 2015.
32. Myers NL. Culture, stress and recovery from schizophrenia: lessons from the field for global mental health. Cult Med Psychiatry. 2010;34(3):500-28.
33. McKinstry C, Brown T, Gustafsson L. Scoping reviews in occupational therapy: the what, why, and how to. Aust Occup Ther J. 2014;61(2):58-66.
34. Tricco AC, Lillie E, Zarin W, et al. Prisma extension for scoping reviews (prisma-scr): Checklist and explanation. Ann Intern Med. 2018;169(7):467-73.
35. Pham MT, Rajić A, Greig JD, Sargeant JM, Papadopoulos A, McEwen SA. A scoping review of scoping reviews: advancing the approach and enhancing the consistency. Research Synthesis Methods. 2014;5(4):371-85.
36. Levac D, Colquhoun H, O'Brien KK. Scoping studies: advancing the methodology. Implementation science. 2010;5(1):69.
37. Slade M, Longden E. Empirical evidence about recovery and mental health. BMC Psychiatry. 2015;15:285.
38. Morgan DL. Pragmatism as a Paradigm for Social Research. Qualitative Inquiry. 2014;20(8):1045-53.

For peer review only - http://bmjopen.bmj.com/site/about/guidelines.xhtml

Preferred Reporting Items for Systematic reviews and Meta-Analyses extension for Scoping Reviews (PRISMA-ScR) Checklist

SECTION	ITEM	PRISMA-ScR CHECKLIST ITEM	REPORTED ON PAGE #
**TITLE**			
Title	1	Identify the report as a scoping review.	1
**ABSTRACT**			
Structured summary	2	Provide a structured summary that includes (as applicable): background, objectives, eligibility criteria, sources of evidence, charting methods, results, and conclusions that relate to the review questions and objectives.	2
**INTRODUCTION**			
Rationale	3	Describe the rationale for the review in the context of what is already known. Explain why the review questions/objectives lend themselves to a scoping review approach.	6
Objectives	4	Provide an explicit statement of the questions and objectives being addressed with reference to their key elements (e.g., population or participants, concepts, and context) or other relevant key elements used to conceptualize the review questions and/or objectives.	7
**METHODS**			
Protocol and registration	5	Indicate whether a review protocol exists; state if and where it can be accessed (e.g., a Web address); and if available, provide registration information, including the registration number.	1
Eligibility criteria	6	Specify characteristics of the sources of evidence used as eligibility criteria (e.g., years considered, language, and publication status), and provide a rationale.	11
Information sources*	7	Describe all information sources in the search (e.g., databases with dates of coverage and contact with authors to identify additional sources), as well as the date the most recent search was executed.	8
Search	8	Present the full electronic search strategy for at least 1 database, including any limits used, such that it could be repeated.	9
Selection of sources of evidence†	9	State the process for selecting sources of evidence (i.e., screening and eligibility) included in the scoping review.	11
Data charting process‡	10	Describe the methods of charting data from the included sources of evidence (e.g., calibrated forms or forms that have been tested by the team before their use, and whether data charting was done independently or in duplicate) and any processes for obtaining and confirming data from investigators.	12
Data items	11	List and define all variables for which data were sought and any assumptions and simplifications made.	12
Critical appraisal of individual sources of evidence§	12	If done, provide a rationale for conducting a critical appraisal of included sources of evidence; describe the methods used and how this information was used in any data synthesis (if appropriate).	13

For peer review only - http://bmjopen.bmj.com/site/about/guidelines.xhtml

SECTION	ITEM	PRISMA-ScR CHECKLIST ITEM	REPORTED ON PAGE #
Synthesis of results	13	Describe the methods of handling and summarizing the data that were charted.	13
**RESULTS**			
Selection of sources of evidence	14	Give numbers of sources of evidence screened, assessed for eligibility, and included in the review, with reasons for exclusions at each stage, ideally using a flow diagram.	Click here to enter text.
Characteristics of sources of evidence	15	For each source of evidence, present characteristics for which data were charted and provide the citations.	Click here to enter text.
Critical appraisal within sources of evidence	16	If done, present data on critical appraisal of included sources of evidence (see item 12).	Click here to enter text.
Results of individual sources of evidence	17	For each included source of evidence, present the relevant data that were charted that relate to the review questions and objectives.	Click here to enter text.
Synthesis of results	18	Summarize and/or present the charting results as they relate to the review questions and objectives.	Click here to enter text.
**DISCUSSION**			
Summary of evidence	19	Summarize the main results (including an overview of concepts, themes, and types of evidence available), link to the review questions and objectives, and consider the relevance to key groups.	Click here to enter text.
Limitations	20	Discuss the limitations of the scoping review process.	3
Conclusions	21	Provide a general interpretation of the results with respect to the review questions and objectives, as well as potential implications and/or next steps.	14
**FUNDING**			
Funding	22	Describe sources of funding for the included sources of evidence, as well as sources of funding for the scoping review. Describe the role of the funders of the scoping review.	14

JBI = Joanna Briggs Institute; PRISMA-ScR = Preferred Reporting Items for Systematic reviews and Meta-Analyses extension for Scoping Reviews.

* Where *sources of evidence* (see second footnote) are compiled from, such as bibliographic databases, social media platforms, and Web sites.

† A more inclusive/heterogeneous term used to account for the different types of evidence or data sources (e.g., quantitative and/or qualitative research, expert opinion, and policy documents) that may be eligible in a scoping review as opposed to only studies. This is not to be confused with *information sources* (see first footnote).

‡ The frameworks by Arksey and O'Malley (6) and Levac and colleagues (7) and the JBI guidance (4, 5) refer to the process of data extraction in a scoping review as data charting.

§ The process of systematically examining research evidence to assess its validity, results, and relevance before using it to inform a decision. This term is used for items 12 and 19 instead of "risk of bias" (which is more applicable to systematic reviews of interventions) to include and acknowledge the various sources of evidence that may be used in a scoping review (e.g., quantitative and/or qualitative research, expert opinion, and policy document).

From: Tricco AC, Lillie E, Zarin W, O'Brien KK, Colquhoun H, Levac D, et al. PRISMA Extension for Scoping Reviews (PRISMA-ScR): Checklist and Explanation. Ann Intern Med. ;169:467–473. doi: 10.7326/M18-0850

For peer review only - http://bmjopen.bmj.com/site/about/guidelines.xhtml

BMJ Open

An exploration of recovery of people living with severe mental illness (SMI) in low- and- middle -income countries (LMIC): a scoping review protocol

Journal:	*BMJ Open*
Manuscript ID	bmjopen-2019-032912.R1
Article Type:	Protocol
Date Submitted by the Author:	15-Oct-2019
Complete List of Authors:	Gamieldien, Fadia; University of Cape Town, Psychiatry; University of Cape Town, Health and Rehabilitation Sciences<br>Galvaan, Roshan ; University of Cape Town, Health and Rehabilitation Sciences<br>Myers, Bronwyn; South African Medical Research Council, Alcohol and Drug Abuse Research Unit<br>Sorsdahl, Katherine; University of Cape Town, Psychiatry
**Primary Subject Heading**:	Public health
Secondary Subject Heading:	Mental health, Public health
Keywords:	recovery, severe mental illness, scoping review, low-and-middle- income countries

SCHOLARONE™
Manuscripts

I, the Submitting Author has the right to grant and does grant on behalf of all authors of the Work (as defined in the below author licence), an exclusive licence and/or a non-exclusive licence for contributions from authors who are: i) UK Crown employees; ii) where BMJ has agreed a CC-BY licence shall apply, and/or iii) in accordance with the terms applicable for US Federal Government officers or employees acting as part of their official duties; on a worldwide, perpetual, irrevocable, royalty-free basis to BMJ Publishing Group Ltd ("BMJ") its licensees and where the relevant Journal is co-owned by BMJ to the co-owners of the Journal, to publish the Work in this journal and any other BMJ products and to exploit all rights, as set out in our licence.

The Submitting Author accepts and understands that any supply made under these terms is made by BMJ to the Submitting Author unless you are acting as an employee on behalf of your employer or a postgraduate student of an affiliated institution which is paying any applicable article publishing charge ("APC") for Open Access articles. Where the Submitting Author wishes to make the Work available on an Open Access basis (and intends to pay the relevant APC), the terms of reuse of such Open Access shall be governed by a Creative Commons licence – details of these licences and which Creative Commons licence will apply to this Work are set out in our licence referred to above.

Other than as permitted in any relevant BMJ Author's Self Archiving Policies, I confirm this Work has not been accepted for publication elsewhere, is not being considered for publication elsewhere and does not duplicate material already published. I confirm all authors consent to publication of this Work and authorise the granting of this licence.

For peer review only

An exploration of recovery of people living with severe mental illness (SMI) in low- and- middle -income countries (LMIC): a scoping review protocol

Fadia Gamieldien[1], ****Roshan Galvaan[2], Bronwyn Myers*,[3] **Katherine Sorsdahl [1]

[1] Alan J. Flisher Centre for Public Mental Health, Department of Psychiatry and Mental Health, University of Cape Town, Cape Town, South Africa

2. Division of Occupational Therapy, Department of Health and Rehabilitation Sciences, University of Cape Town, Cape Town, South Africa

3 Alcohol, Tobacco and Other Drug Research Unit, South African Medical Research Council and Division of Addiction Psychiatry, Department of Psychiatry and Mental Health, University of Cape Town,Cape Town, South Africa

Corresponding Author: Ms Fadia Gamieldien

Orcid number 0000-0003-2820-6484

Address: Alan J Flisher Centre for Public Mental Health

Department of Psychiatry and Mental Health

University of Cape Town

46 Sawkins Road

Rondebosch, 7700

Cape Town

South Africa

Fadia.Gamieldien@uct.ac.za

+27 82 974 7468

Keywords

Recovery, severe mental illness, low-and-middle-income countries, scoping review

Declaration of Interest: None

For peer review only

ABSTRACT

Introduction: The construct of recovery was conceptualised in high income countries and its applicability in low-and- middle- income countries is underexplored. A scoping review is proposed to synthesise knowledge, review conceptual overlap and map key elements of recovery from severe mental illness in low-and- middle- income countries. We aim to appraise the literature so as to inform future recovery-oriented services that consider the cultural and contextual influences on recovery from severe mental illness.

Methods and analysis: The following electronic databases: MEDLINE via PubMed, SCOPUS (which included contents of Embase), PsycINFO, CINAHL, Africa-Wide Information, PSYCHarticles, Health source: Nursing/Academic Edition, Academic Search Premier and SocINDEX all via the EBSCOHOST platform, the Latin American and Caribbean Health Sciences Literature (LILACS), the Cochrane Centre Register of Controlled Trials) and grey literature sources will be searched during the period May- June 2019. Eligible studies will be independently screened for inclusion and exclusion by two reviewers using a checklist developed for this purpose. Studies published between 1993 and 2019 that focuses on recovery from severe mental illness in a low-and -middle- income country will be included. Findings will be compared, and discrepancies will be discussed. Unresolved discrepancies will be referred to a third reviewer. All bibliographic data and study characteristics will be extracted and thematically analysed using a tool developed through an iterative process by the research team. Indicators will be classified according to a predefined conceptual framework and categorised and described using qualitative content analysis.

Ethics and dissemination: The review aims at synthesising information from available publications hence it does not require ethical approval. The results will be disseminated through publications, conference presentations and future workshops with stakeholders involved within the recovery paradigm of mental health policy and practice. The scoping review title is registered with the Joanna Briggs Institute.

For peer review only - http://bmjopen.bmj.com/site/about/guidelines.xhtml

Strengths and limitations of the study

- This scoping review allows for the systematic synthesis of knowledge on recovery from severe mental illness in to low- and middle-income countries in a rigorous and methodological manner
- This review will highlight the diversity of severe mental illness since there is no agreed upon criteria for including mental illnesses into this category
- Knowledge gaps related to recovery as a multidimensional construct in low- and middle-income countries will be identified
- Only conceptual and theoretical underpinnings of recovery as a construct in LMIC will be discussed without a focus on intervention outcomes.
- Data synthesis will be limited to full text articles available in English only published between 1993 and 2019. Grey literature and unpublished studies in Latin America will be affected by the decision to exclude articles not available in English.

Word count: 3310 (excludes abstract, strengths and limitations, table and references)

For peer review only

INTRODUCTION

Mental, neurological and substance use (MNS) disorders contribute significantly to the global burden of disease. Whiteford, Ferrari, Degenhardt, Feigin, and Vos (2015) report that MNS disorders account for 10% of disability-adjusted life years (DALYs). However, it has been argued that the true global burden of mental health problems may be underestimated by more than a third and that DALYs are closer to 13%[1]. The lifetime prevalence of severe mental illness (SMIs) ranges between 1% and 4%[2]. Although this is relatively low in comparison to common mental disorders (CMDs), SMIs are more disabling and require complex, long-term interventions for the person and their family[1]. Given the move towards deinstitutionalisation of people with psychiatric disorders, shorter hospital stays are resulting in premature discharge rates and repeated relapse, creating a revolving door phenomenon[3]. These frequent hospital readmissions are costly to the health care system[4].

In low- and- middle- income countries (LMICs) many people living with a SMI are not detected and as a result do not receive the treatment they need[2, 5, 6]. In a service utilisation review of World Health Organisation member countries, it was found that the lifetime prevalence of mental disorders amongst adults was between 12% to 49%[5]. The review found the treatment gap to be wide across the psychiatric disorders examined. The treatment gap for schizophrenia and non-affective psychosis was 32%. The gap for other disorders was: major depressive disorder, 56%; bipolar disorder, 50%; and alcohol abuse and dependence, 78% [5]. While present in all countries, this treatment gap is greatest in LMICs where there is a need to scale up mental health services[7].

A systematic review on the scaling up of mental health services in LMICs found that in many countries the majority of resources for mental health care are rendered on an inpatient or outpatient basis at large psychiatric hospitals, with little provision of services at primary healthcare facilities or via integration between hospitals and the community[7]. Despite the drive for deinstitutionalisation and for the rendering of services at decentralised locations, in many countries most of the mental health care budget continues to be allocated to large psychiatric hospitals. In these hospitals, the treatment approach is primarily through medication to alleviate acute clinical symptoms, giving little to no attention to the psychosocial needs of service users[3, 7, 8]. Unfortunately the sole focus on clinical recovery and the limited availability of interventions that focus on rehabilitation and personal recovery further exacerbates the revolving door phenomenon and ongoing treatment gap[3, 9].

Recovery oriented approaches offer an extension to medical models of intervention, as they are not solely focused on symptomatic improvement as a marker of clinical recovery[10]. Personal recovery for people with SMI is conceptually distinct from the medical definition of

For peer review only - http://bmjopen.bmj.com/site/about/guidelines.xhtml

recovery which is clinical in nature and mostly values symptom remission[11, 12]. Personal recovery proposes that a person develops new meaning and purpose in their life over the longitudinal course of their illness[10, 13]. Psychiatric rehabilitation has highlighted the need to move beyond treating the symptoms of the illness, to treating its functional and disabling consequences as well [14]. Deegan, a mental health professional and person living with schizophrenia, suggested that mental health service users do not "get rehabilitated" by others, but instead they are active participants in their journey to recover new and valued personal meaning and purpose[15].

The recovery model was initiated in high income countries (HICs) with the mental health consumer and survivor movement at a time when having a diagnosis of SMI was highly stigmatised and the prognosis was poor[15]. The idea that people with an SMI cannot recover was challenged by people living with schizophrenia who were leading meaningful lives in their communities after deinstutionalisation, highlighting that recovery was not dependant on long term psychiatric treatment[16, 17]. For a person living with an SMI, receiving a diagnosis and psychiatric treatment focussed on clinical recovery is a necessary step towards recovery, but personal recovery is also critical given the cyclical, non-linear, long term course of the illness. In Anthony's seminal work[14], he defined recovery as:

"*a deeply personal, unique process of changing ones' attitude, values, feelings, goals, skills and/or roles. It is a way of living a satisfying, hopeful and contributing life even within the limitations caused by illness"*

Research exploring the meaning of recovery among people living with SMI[18-20] found that recovery means different things to different people, supporting a view that there is no definition of recovery that applies to everyone. A literature review conducted in 2018 explored the meaning of recovery amongst people living with schizophrenia[18]. This review included 17 studies explaining the subjective meaning of recovery. A majority of the included studies were qualitative, with a few taking a quantitative approach to exploring the meaning of recovery. Of the 17 papers only four studies were from LMICs (India (n=2), China and South Africa). The results revealed that recovery is complex, non-linear and perceived as both a process and an outcome. The process was viewed as being individual and it was recommended that more consumer perspectives to inform recovery-oriented interventions which are aligned to individuals' personal goals and aspirations were needed. The complexity embedded in viewing recovery as a process and an outcome with multidimensional indicators has been documented by others[21-23]. Further to this, it was recommended that clinicians, caregivers and researchers explore more qualitative and personal narratives of recovery, the tools needed to measure recovery and the development of community-based recovery- oriented services[18].

For peer review only - http://bmjopen.bmj.com/site/about/guidelines.xhtml

The aforementioned review was limited to recovery in people living with Schizophrenia and only included four studies from LMIC countries. There is current debate around whether people with SMI in LMICs have a better prognosis and a call to re-examine the realities of living with an SMI amidst social, cultural and economic changes in LMICs [24, 25]. Conducting a scoping review focusing specifically on studies conducted in LMICs will contribute to the literature on recovery in these specific contexts and will be useful for the following reasons. First, little attention has been given to describing recovery as a personal and individual journey from LMIC perspectives. As recovery for the person with an SMI takes place in a social context through a range of relationships, more understanding about how contexts can help or hinder recovery is needed[26].

Second, individual meaning-making within recovery is influenced by social, political, economic and human rights factors but further research is needed to understand the diversity of recovery narratives from the perspectives of different population groups [27]. A systematic review and narrative analysis synthesising descriptions and models of personal recovery was undertaken to inform the development of a conceptual framework to guide recovery oriented research and practice[9]. Studies conducted in HIC specifically the United States featured predominantly in the review which included 97 papers. The review culminated in the emergence of a conceptual framework to describe the characteristics of recovery, the processes of recovery and the stages of recovery. The authors reported a difference in studies which included ethnic minorities and where more emphasis was placed on stigma, spirituality, culture and the collectivist nature of recovery. They acknowledged that this area is underexplored[9].

Third, in many African cultures an individual's personhood is manifested through their agency in relation to spirituality, society and self[28]. This may impact on the person and how they experience mental illness and recovery. Understanding cultural concepts and the dynamics of interconnectedness means recognising that personhood in LMICs is weaved into how people experience health, illness and recovery[28]. Considering the person as a spiritual and social being provides opportunities to collaborate with caregivers and communities in developing appropriate interventions[28]. In countries that are spiritually diverse, multicultural and home to people of different religious backgrounds it is important to contextualise mental illness and explore health professionals own perspectives on culture, psychiatry and mental health and where mental illness must be contextualised[29, 30]. Given the reasons cited previously, further exploration into the recovery journeys of people living with SMI in LMICs and how they view the dimensions of recovery is warranted[18].

A scoping review is proposed as a means of synthesising knowledge on a broadly defined topic and systematically mapping key concepts, theories, evidence and research gaps in the

For peer review only

topic area, while still being rigorous and methodological in its approach[31, 32]. While systematic reviews and meta-analyses limit their parameters to research trials and quantitative data synthesis, scoping reviews adopt a broader approach to the narrative integration of evidence[33]. We aim to appraise the definitions of recovery and its current utilisation for people with SMI in LMICs. The review findings could be used to inform future practice applications which considers the cultural and contextual factors impacting on recovery[34] from SMI outside of HICs. This scoping review title is registered with the Joanna Briggs Institute.

METHODS

A scoping review was deemed as the most suitable method to map existing research on recovery from SMI in LMICs because it allows for the topic to be located, examined, summarised and be presented rapidly and systematically. Additionally it could serve as a precursor to a systematic review [35]. The proposed review will be conducted using the Arksey and O'Malley[32] scoping review methodology and will be reported on using the PRISMA guidelines[36] with an understanding that there is much development as scoping reviews gain popularity especially in health-related topics [31, 37].

Arksley and O'Malley [32] offer a methodological framework for conducting a scoping review. Their approach describes six stages: (1) identification of the research question, (2) identification of relevant studies, (3) selection of studies, (4) charting the data, (5) collating, summarising and reporting of results and (6) consultation with stakeholders [32]. The last stage is optional according to Arksley and O'Malley[32] but others have deemed it a necessary step to aide in methodological rigour[38]. Recommendations to enhance the methodology at each stage will be incorporated into this review[31, 36, 38].

Stage 1: Identifying the research question

In order to guide the search strategy an iterative process of discussion amongst authors occurred in order to develop and refine the research question. The question needed to be clear enough to inform the subsequent stages while still reflecting the scope of inquiry[38]. Given that scoping studies are focused on summarizing the breadth of evidence on a given topic the authors refined the exploratory question to ask the following:

What is known about recovery from severe mental illness in low-and-middle-income countries?

This broad question was developed to allow for a comprehensive review of the literature in order to review and appraise the definition of recovery and how it is understood in mental

For peer review only

health research and practice in LMICs. While many have written about this in high income countries[20, 39] there is a dearth of such information in LMICs .

Stage 2: Identifying relevant studies

Given the aim of the study is to identify primary published and unpublished studies which will answer the research question, a comprehensive search strategy was developed to aide this. Two health sciences librarians assisted the first author in the iterative process of developing a search strategy including inclusion and exclusion criteria. Decisions were made about time frames, language, search terms and search strategies. Trial searches were run to assess whether relevant literature could be identified using the proposed strategy. A discussion of the identified databases, search strategy and selection criteria follow.

Databases

Electronic databases will be searched to identify studies published between January 1993-June 2019. This period was selected as it covers roughly 25 years in the recovery movement, starting with Anthony's initial definition of recovery from mental illness[14]. An expert librarian assisted the first author in identifying information sources that are relevant to the research question. The following databases were selected: MEDLINE via PubMed, SCOPUS (which included contents of Embase), PsycINFO, CINAHL, Africa-Wide Information, PSYCH articles, Health source: Nursing/Academic Edition, Academic Search Premier and SocINDEX all via the EBSCOHOST platform. Additionally, the Latin American and Caribbean Health Sciences Literature (LILACS), the Cochrane Centre Register of Controlled Trials. Grey literature sources will be identified to expand the search and enhance the data sources. Additional sources will be located by means of hand searching reference lists of relevant papers, contacting study authors, searching trial registers, and contacting key personnel involved in recovery-focussed programmes in LMICs. In this way peer reviewed and grey literature from the biomedical sciences, allied health sciences, social sciences and other disciplinary fields will be included.

Search Strategy

Two librarians and the first author developed the eligibility criteria for the scoping review. They identified various definitions of severe mental illness as well as recovery to include in this search strategy. A list of preliminary search terms and filtering methods was developed. The main filtering methods related to the date range of 25 years (1993-2018). This was extended to include January- June 2019 so that new publications would not be missed. A preliminary search was conducted to test the search strategy. Thereafter, the strategy was refined to include MESH terms, filters and Boolean operations to comply with searches across different

For peer review only - http://bmjopen.bmj.com/site/about/guidelines.xhtml

databases. A search strategy for the PubMed database is included in Supplementary file 1. After the initial search, key words and index terms used across the databases were identified and plotted on a table as indicated below (Table 1).

Table 1 Search strategy terms

Keyword	Alternative
Recovery	Recovery OR recover OR psychosocial rehabilitation OR mental health rehabilitation OR psychiatric rehabilitation OR mental health recovery OR Recovery of function OR Quality of life
Severe mental illness	Severe mental illness OR bipolar OR delusional disorder OR delusional disorders OR major depressive disorder OR major depressive disorders OR schizophrenia OR manic OR manic-depressive OR paranoid disorder OR paranoid disorders OR psychoses OR psychosis OR psychotic disorder OR psychotic disorders OR schizoaffective disorder OR schizoaffective disorders OR Schizophreniform OR serious mental disorder OR serious mental disorders<br>Schizophrenia Spectrum and Other Psychotic Disorders<br>Bipolar and Related Disorders<br>Depressive Disorder
Low- and-middle- income countries	Deprived Countries OR Deprived Population OR Deprived Populations OR Developing Countries OR Developing Country OR Developing Economies OR Developing Economy OR Developing Nation OR Developing Nations OR Developing Population OR Developing Populations OR Developing World OR LAMI Countries OR LAMI Country OR Less Developed Countries OR Less Developed Country OR Less Developed Economies OR Less Developed Nation OR Less Developed Nations OR Less Developed World OR Lesser Developed Countries OR Lesser Developed Nations OR LMIC OR LMICS OR Low GDP OR Low GNP OR Low Gross Domestic OR Low Gross National OR Low Income Countries OR Low Income Country OR Low Income Economies OR Low Income Economy OR Low Income Nations OR Low Income Population OR Low Income Populations OR Lower GDP OR lower gross domestic OR Lower Income Countries OR Lower Income Country OR Lower Income Nations OR Lower Income Population OR Lower Income Populations OR Middle Income Countries OR Middle Income Country OR Middle Income Economies OR Middle Income Nation OR Middle Income Nations OR Middle Income Population OR Middle Income Populations OR Poor Countries OR Poor Country OR Poor Economies OR Poor Economy OR Poor Nation OR Poor Nations OR Poor Population OR Poor Populations OR poor world OR Poorer Countries OR Poorer Economies OR Poorer Economy OR Poorer Nations OR Poorer Population OR Poorer Populations OR Third World OR Transitional Countries OR Transitional Country OR Transitional Economies OR Transitional

For peer review only

	Economy OR Under Developed Countries OR Under Developed Country OR under developed nations OR Under Developed World OR Under Served Population OR Under Served Populations OR Underdeveloped Countries OR Underdeveloped Country OR underdeveloped economies OR underdeveloped nations OR underdeveloped population OR Underdeveloped World OR Underserved Countries OR Underserved Nations OR Underserved Population OR Underserved Populations<br><br>OR<br>Afghanistan OR Albania OR Algeria OR American Samoa OR Angola OR Armenia OR Azerbaijan OR Bangladesh OR Belarus OR Byelarus OR Belorussia OR Belize OR Benin OR Bhutan OR Bolivia OR Bosnia OR Botswana OR Brazil OR Bulgaria OR Burma OR Burkina Faso OR Burundi OR Cabo Verde OR Cape Verde OR Cambodia OR Cameroon OR Central African Republic OR Chad OR China OR Colombia OR Comoros OR Comores OR Comoro OR Congo OR Costa Rica OR Côte d'Ivoire OR Cuba OR Djibouti OR Dominica OR Dominican Republic OR Ecuador OR Egypt OR El Salvador OR Equatorial Guinea OR Eritrea OR Ethiopia OR Fiji OR Gabon OR Gambia OR Gaza OR Georgia OR Georgia Republic OR Ghana OR Grenada OR Grenadines OR Guatemala OR Guinea OR Guinea- Bissau OR Guyana OR Haiti OR Herzegovina OR Hercegovina OR Honduras OR India OR Indonesia OR Iran OR Iraq OR Jamaica OR Jordan OR Kazakhstan OR Kenya OR Kiribati OR Democratic People's Republic of Korea OR Kosovo OR Kyrgyz OR Kirghizia OR Kirghiz OR Kyrgyzstan OR Lao PDR OR Laos OR Lebanon OR Lesotho OR Liberia OR Libya OR Macedonia OR Madagascar OR Malawi OR Malay OR Malaya OR Malaysia OR Maldives OR Mali OR Marshall Islands OR Mauritania OR Mauritius OR Mexico OR Micronesia OR Moldova OR Mongolia OR Montenegro OR Morocco OR Mozambique OR Myanmar OR Namibia OR Nepal OR Nicaragua OR Niger OR Nigeria OR Pakistan OR Palau OR Panama OR Papua New Guinea OR Paraguay OR Peru OR Philippines OR Principe OR Romania OR Ruanda OR Rwanda OR Samoa OR Sao Tome OR Senegal OR Serbia OR Sierra Leone OR Solomon Islands OR Somalia OR South Africa OR South Sudan OR Sri Lanka OR St Lucia OR St Vincent OR Sudan OR Surinam OR Suriname OR Swaziland OR Syria OR Syrian Arab Republic OR Tajikistan OR Tadzhikistan OR Tajikistan OR Tadzhik OR Tanzania OR Thailand OR Timor OR Togo OR Tonga OR Tunisia OR Turkey OR Turkmen OR Turkmenistan OR Tuvalu OR Uganda OR Ukraine OR Uzbek OR Uzbekistan OR Vanuatu OR Venezuela OR Vietnam OR West Bank OR Yemen OR Zambia OR Zimbabwe

For peer review only

Selection criteria

Articles will be included in the review if they meet the following inclusion and exclusion criteria (Table 2).

Table 2 Inclusion and exclusion criteria

Criteria for inclusion	Criteria for exclusion
• Published between January 1993- March 2019<br>• Qualitative and quantitative empirical (i.e. primary research) study designs<br>• Theoretical literature on the construct of recovery<br>• Studies will be included if they have been conducted in LMICs<br>• Peer reviewed scientific literature and “grey literature” from LMICs (literature found outside of formal academic publishing) will be considered<br>• Literature focussing on the population of interest; that is adults with SMI in LMICs will be included. The diagnosis of SMI includes schizophrenia spectrum disorders, schizoaffective disorders, bipolar affective disorders, major depressive disorder or related psychotic disorders (schizophreniform, delusional disorder, substance induced psychotic disorder or disorders not otherwise specified) as well as those with a comorbid substance use disorder. Studies and reports will be excluded if they do not include the keywords or alternatives as outlined in table 1	• The review will be limited to full text articles written in English. However, the authors acknowledge that this practical decision could mean that potentially relevant papers are missed<br>• Conference abstracts will be excluded although they will be reviewed to see if full reports were published<br>• Children will be excluded as the age range for inclusion is 18 years and older<br>• Duplicate articles from the same study will be excluded<br>• Policy documents will be excluded although they will be reviewed to access research in LMICs that they might have referenced<br>• Studies that occurred only in high income countries, without a LMIC focus will be excluded<br>• Studies must be conducted in LMIC and not in HIC with particpants from LMICs

For peer review only

• Explicit mention of severe mental illness and recovery in LMICS	

Stage 3: Study selection

The scoping review guideline for identifying and selecting studies informs article selection and data extraction [32]. Two screening levels are proposed: a title and abstract review and a full-text review. For the first level of screening, two authors (FG, ZS) will independently screen the title and abstracts of all retrieved citations against a few select inclusion criteria. Any articles that seem relevant by either or both reviewers will be included in the full-text review. Next, these two reviewers will independently screen the full text articles to assess whether they meet the inclusion and exclusion criteria. At both levels of screening, inter-rater agreement will be assessed by calculating Cohen's Kappa. Any disagreement on study selection will be resolved by discussion with a third rater, who will be a senior member of the research team.

Quality appraisal

The Mixed Methods Appraisal Tool (MMAT) version 2018[40] and the Critical Appraisal Skills Programme (CASP) Qualitative checklist will used to assess the quality of included studies although this is not required for a scoping review nor will it influence the inclusion of the study.

Using the aforementioned criteria, the number of studies identified and selected for inclusion in the scoping review will be reported on. A narrative description[31] will accompany the search decision flow chart[36]. The flow chart will show the results from the search, the removal of duplicates, study selection, full retrieval numbers, additional searches from reference lists and a final summary of decisions related to the search decision and an explanation of the review decision process[31].

Stage 4: Charting the data

Next, the two reviewers (FG and ZS) will independently extract data from the identified articles and chart it[32]. A draft data charting formwill be created in the MS Excel programme to facilitate this process[32]. This form will be pre-tested and an iterative process of refining the form will be undertaken as the review commences to ensure that all relevant information is extracted[31].

The form will allow the reviewers to confirm study relevance, record study characteristics and extract information relevant to the review question. The following information will be extracted from the articles: 1) study title; 2) author; 3) study population; 4) participant characteristics; 5)

For peer review only - http://bmjopen.bmj.com/site/about/guidelines.xhtml

research question; 6) study methodology; 7) study description; 8) intervention type (if applicable); 9) intervention duration (if applicable); 10) outcome measures; 11 summary of findings; 12) definitions of key concepts (recovery and severe mental illness); 13) conceptual links identified; 14) practice implications and; 15) recommendations for further development.

While charting the data it is important to maintain flexibility around including emerging categories and consulting with the team as this occurs. The final version of the charting form along with definitions of included items will be provided as an appendix to the review. This process will facilitate the timeous completion of the review while also maintaining research rigour.

Stage 5: Collating, summarising and reporting the results

All results will be collated, summarised and reported on in order to provide a comprehensive and thorough overview of all the reviewed literature. The authors plan to follow an iterative process and not pre-empt or fit findings into what has already been written about recovery in HICs. This will allow new understandings to be uncovered. A three-step approach[38] of analysing the data, reporting results and applying meaning to results will be followed to provide methodological rigour. Collating and summarising results will include a summary of the types of studies conducted (including the quantity, types of study design, populations, setting) as well as a thematic analysis of the results. More specifically, we will code how studies describe the concept of recovery from SMI and how it is defined and understood in LMICs. The framework used to report the findings will be developed iteratively through the examination of results. Additionally, the authors will report on the broader implications of the review in terms of research, policy and practice.

Stage 6: Consultation

While Arksey and O'Malley[32] view the consultation stage as an optional step, Levac[38] views it as a requirement to ensure methodological rigour. During each stage of the review process we will consult with an existing stakeholder advisory committee. This advisory committee consists of the research team and key stakeholders including mental health service providers and service users from within the public health and non-profit organisation (NPO) sectors. This committee has been constituted from the onset of the project and some members are part of a pre-existing group for public mental health specialists. The advisory committee will be consulted to get their views on the relevance of the review, guidance for accessing grey literature and their perspectives on the data extraction process and preliminary findings. Including an advisory committee in the review is informed by a pragmatic approach[41]where the research is treated as a human experience with the understanding that this consultation

For peer review only - http://bmjopen.bmj.com/site/about/guidelines.xhtml

process is likely to enhance the relevance of this review. It will also support collaborative efforts to facilitate consumer participation, public involvement, access to information and transfer of knowledge[38].

Patient and public involvement

There is no patient involvement in the protocol development aspect of the study. Service users forming part of the advisory committee referred to in Stage 6 will be consulted on findings and dissemination of results.

Ethics and dissemination

Since the scoping review methodology aims at synthesising information from available publications, this study does not require ethical approval. Reviews of primary research allows for the mapping of evidence in fields where the body of literature has not yet been comprehensively reviewed or where working definitions and concepts are still developing. The findings will be made available in different formats to facilitate its dissemination. An article reporting on the results will be submitted for publication in a peer -reviewed journal. It will also be presented at relevant conferences and stakeholder engagements involved in mental health policy and practice. These stakeholders include MHSUs, academics, policy makers, researcher and clinicians. Findings will also be shared via newsletters, policy briefs and social media forums.

Conclusion

This scoping review will cover the breadth of literature examining recovery from SMI in LMICs. We anticipate results which highlight research gaps and a dearth of information on how recovery is described in the literature from perspectives outside of high-income countries. Since we aim to appraise the definitions of recovery and its current utilisation for people with SMI in LMICs, the review findings could be used to inform adaptations to the current understanding of recovery to enhance its applicability to MHSUs in LMICs. This could form the basis for future research and inform new practice directions.

Author Contributions

This protocol was developed by the intellectual contributions of all the authors. All authors were involved in developing the review question and conceptualising the approach. FG developed and tested the search terms in consultation with a subject librarian. FG and KS developed the data extraction guideline, and this was revised based on feedback from the other authors. RG, KS and BM provided substantial critique and review of the draft protocol.

For peer review only - http://bmjopen.bmj.com/site/about/guidelines.xhtml

Funding

This work was supported by the DELTAS Africa Initiative [DEL-15-01]. The DELTAS Africa Initiative is an independent funding scheme of the African Academy of Sciences (AAS)'s Alliance for Accelerating Excellence in Science in Africa (AESA) and supported by the New Partnership for Africa's Development Planning and Coordinating Agency (NEPAD Agency) with funding from the Wellcome Trust [DEL-15-01] and the UK government. The views expressed in this publication are those of the author(s) and not necessarily those of AAS, NEPAD Agency, WellcomeTrust or the UK government. BM is funded through the South African Medical Research Council.

For peer review only

For peer review only - http://bmjopen.bmj.com/site/about/guidelines.xhtml

References

1. Vigo D, Thornicroft G, Atun R. Estimating the true global burden of mental illness. The Lancet Psychiatry. 2016;3(2):171-8.
2. Kessler RC, Berglund PA, Bruce ML, Koch JR, Laska EM, Leaf PJ, et al. The prevalence and correlates of untreated serious mental illness. Health Serv Res. 2001;36(6 Pt 1):987.
3. Petersen I, Lund C, Stein DJ. Optimizing mental health services in low-income and middle-income countries. Current opinion in psychiatry. 2011;24(4):318-23.
4. Sfetcu R, Musat S, Haaramo P, Ciutan M, Scintee G, Vladescu C, et al. Overview of post-discharge predictors for psychiatric re-hospitalisations: a systematic review of the literature. BMC Psychiatry. 2017;17(1).
5. Kohn R, Saxena S, Levav I, Saraceno B. The treatment gap in mental health care. World Health Organization Bulletin of the World Health Organization. 2004;82(11):858-66.
6. Demyttenaere K, Bruffaerts R, Posada-Villa J, Gasquet I, Kovess V, Lepine JP, et al. Prevalence, severity, and unmet need for treatment of mental disorders in the World Health Organization World Mental Health Surveys. Jama. 2004;291(21):2581-90.
7. Eaton J, McCay L, Semrau M, Chatterjee S, Baingana F, Araya R, et al. Scale up of services for mental health in low-income and middle-income countries. The Lancet. 2011;378(9802):1592-603.
8. Lund C, Oosthuizen P, Flisher AJ, Emsley R, Stein DJ, Botha U, et al. Pathways to Inpatient Mental Health Care Among People With Schizophrenia Spectrum Disorders in South Africa. Psychiatr Serv. 2010;61(3):235-40.
9. Leamy M, Bird V, Le Boutillier C, Williams J, Slade M. Conceptual framework for personal recovery in mental health: systematic review and narrative synthesis. Br J Psychiatry. 2011;199(6):445-52.
10. Slade M. Personal recovery and mental illness: A guide for mental health professionals: Cambridge University Press; 2009.
11. Slade M. Mental illness and well-being: the central importance of positive psychology and recovery approaches. BMC Health Serv Res. 2010;10:26-.
12. Myers NL, Myers NL. Culture, stress and recovery from schizophrenia: lessons from the field for global mental health. Cult Med Psychiatry. 2010;34(3):500-28.
13. Tew J, Ramon S, Slade M, Bird V, Melton J, Le Boutillier C. Social factors and recovery from mental health difficulties: a review of the evidence. The British Journal of Social Work. 2012;42(3):443-60.
14. Anthony WA. Recovery from mental illness: the guiding vision of the mental health service system in the 1990s. Psychosocial rehabilitation journal. 1993;16(4):11.
15. Deegan PE. Recovery: The lived experience of rehabilitation. Psychosocial rehabilitation journal. 1988;11(4):11.
16. Webb L. The recovery model and complex health needs: what health psychology can learn from mental health and substance misuse service provision. J Health Psychol. 2012;17(5):731-41.
17. Farkas M. The vision of recovery today: what it is and what it means for services. World Psychiatric Association (WPA). 2007;6(2):68.
18. Mathew ST, Nirmala BP, Kommu JVS. Meaning of Recovery Among Persons with Schizophrenia: A Literature Review. Journal of Psychosocial Rehabilitation and Mental Health. 2018;5(1):73-81.
19. Kelly M, Lamont S, Brunero S. An Occupational Perspective of the Recovery Journey in Mental Health. Br J Occup Ther. 2010;73(3):129-35.
20. Cabassa LJ, Nicasio A, Whitley R. Picturing Recovery: A Photovoice Exploration of Recovery Dimensions Among People With Serious Mental Illness. Psychiatric services (Washington, DC). 2013;64(9):10.1176/appi.ps.201200503.

For peer review only - http://bmjopen.bmj.com/site/about/guidelines.xhtml

21. Aldersey HM, Adeponle AB, Whitley R. Diverse Approaches to Recovery from Severe Mental Illness. The Palgrave Handbook of Sociocultural Perspectives on Global Mental Health2017. p. 109-27.
22. Frost BG, Tirupati S, Johnston S, Turrell M, Lewin TJ, Sly KA, et al. An Integrated Recovery-oriented Model (IRM) for mental health services: evolution and challenges. BMC Psychiatry. 2017;17:22.
23. Lysaker PH, Hamm JA, Hasson-Ohayon I, Pattison ML, Leonhardt BL. Promoting recovery from severe mental illness: Implications from research on metacognition and metacognitive reflection and insight therapy. World Journal of Psychiatry. 2018;8(1):1-11.
24. Cohen A, Patel V, Thara R, Gureje O. Questioning an axiom: better prognosis for schizophrenia in the developing world? Schizophr Bull. 2008;34(2):229-44.
25. Patel V, Saxena S, Lund C, Thornicroft G, Baingana F, Bolton P, et al. The Lancet Commission on global mental health and sustainable development. The Lancet. 2018;392(10157):1553-98.
26. Schön U-K, Denhov A, Topor A. Social relationships as a decisive factor in recovering from severe mental illness. Int J Soc Psychiatry. 2009;55(4):336-47.
27. Llewellyn-Beardsley J, Rennick-Egglestone S, Callard F, Crawford P, Farkas M, Hui A, et al. Characteristics of mental health recovery narratives: Systematic review and narrative synthesis.(Research Article). Public Library of Science; 2019. p. e0214678.
28. Kpanake L. Cultural concepts of the person and mental health in Africa. Transcultural Psychiatry. 2018;55(2):198-218.
29. Janse van Rensburg ABR. South African Society of Psychiatrists guidelines for the integration of spirituality in the approach to psychiatric practice : guideline. 2014;20(4):133-9.
30. Swartz L, Foster D. Images of culture and mental illness : South African psychiatric approaches. Social Dynamics. 1984;10(1):17-25.
31. Peters MD, Godfrey CM, Khalil H, McInerney P, Parker D, Soares CB. Guidance for conducting systematic scoping reviews. International journal of evidence-based healthcare. 2015;13(3):141-6.
32. Arksey H, O'Malley L. Scoping studies: towards a methodological framework. International Journal of Social Research Methodology. 2005;8(1):19-32.
33. Dijkers M. What is a scoping review?. KT Update, 4 (1). 2015.
34. Myers NL. Culture, stress and recovery from schizophrenia: lessons from the field for global mental health. Cult Med Psychiatry. 2010;34(3):500-28.
35. McKinstry C, Brown T, Gustafsson L. Scoping reviews in occupational therapy: the what, why, and how to. Aust Occup Ther J. 2014;61(2):58-66.
36. Tricco AC, Lillie E, Zarin W, et al. Prisma extension for scoping reviews (prisma-scr): Checklist and explanation. Ann Intern Med. 2018;169(7):467-73.
37. Pham MT, Rajić A, Greig JD, Sargeant JM, Papadopoulos A, McEwen SA. A scoping review of scoping reviews: advancing the approach and enhancing the consistency. Research Synthesis Methods. 2014;5(4):371-85.
38. Levac D, Colquhoun H, O'Brien KK. Scoping studies: advancing the methodology. Implementation science. 2010;5(1):69.
39. Slade M, Longden E. Empirical evidence about recovery and mental health. BMC Psychiatry. 2015;15:285.
40. Hong QN, Pluye P, Fàbregues S, Bartlett G, Boardman F, Cargo M, et al. Mixed methods appraisal tool (MMAT), version 2018. IC Canadian Intellectual Property Office, Industry Canada. 2018.
41. Morgan DL. Pragmatism as a Paradigm for Social Research. Qualitative Inquiry. 2014;20(8):1045-53.

For peer review only

Preliminary searches were run during the periods 10-20 May 2019. Search was fine-tuned with librarian and final searches were run in week of 27-31 May 2019

Table: PubMed Search strategy, modified as needed for other electronic databases

PubMed search run date 29 May 2019

HITS 887

Population, Concept, Context (PCC)

**Population:**		
#1	MeSH terms:	Schizophrenia Spectrum and Other Psychotic Disorders<br>Bipolar and Related Disorders<br>Depressive disorder, Major
#2	Free text:	Severe mental illness OR bipolar OR delusional disorder OR delusional disorders OR major depressive disorder OR major depressive disorders OR schizophrenia OR manic OR manic-depressive OR paranoid disorder OR paranoid disorders OR psychoses OR psychosis OR psychotic disorder OR psychotic disorders OR schizoaffective disorder OR schizoaffective disorders OR Schizophreniform OR serious mental disorder OR serious mental disorders
#3	#1 OR #2	
**Concept:**		
#4	MeSH terms:	Psychiatric Rehabilitation<br>Mental Health Recovery<br>Recovery of Function
#5	Free text:	Recovery OR recover OR psychosocial rehabilitation OR mental health rehabilitation OR psychiatric rehabilitation
#6	#4 OR #5	
**Context: Low and Middle Income Countries (LMICS)**		
#7	Free text:	Deprived Countries OR Deprived Population OR Deprived Populations OR Developing Countries OR Developing Country OR Developing Economies OR Developing Economy OR Developing Nation OR Developing Nations OR Developing Population OR Developing Populations OR Developing World OR LAMI Countries OR LAMI Country OR Less Developed Countries OR Less Developed Country OR Less Developed Economies OR Less Developed Nation OR Less Developed Nations OR Less Developed World OR Lesser Developed Countries OR Lesser Developed Nations OR LMIC OR LMICS OR Low GDP OR Low GNP OR Low Gross Domestic OR Low Gross National OR Low Income Countries OR Low Income Country OR Low Income Economies OR Low Income Economy OR Low Income Nations OR Low Income Population OR Low Income Populations OR Lower GDP OR lower gross domestic OR Lower Income Countries OR Lower Income Country OR Lower Income Nations OR Lower Income Population OR Lower Income Populations OR Middle Income Countries OR Middle Income Country OR Middle Income Economies OR Middle Income Nation OR Middle Income Nations OR Middle Income Population OR Middle Income Populations OR Poor Countries OR Poor Country OR Poor Economies OR Poor Economy OR Poor Nation OR Poor Nations OR Poor Population

		OR Poor Populations OR poor world OR Poorer Countries OR Poorer Economies OR Poorer Economy OR Poorer Nations OR Poorer Population OR Poorer Populations OR Third World OR Transitional Countries OR Transitional Country OR Transitional Economies OR Transitional Economy OR Under Developed Countries OR Under Developed Country OR under developed nations OR Under Developed World OR Under Served Population OR Under Served Populations OR Underdeveloped Countries OR Underdeveloped Country OR underdeveloped economies OR underdeveloped nations OR underdeveloped population OR Underdeveloped World OR Underserved Countries OR Underserved Nations OR Underserved Population OR Underserved Populations
#8	Free text:	Afghanistan OR Albania OR Algeria OR American Samoa OR Angola OR Armenia OR Azerbaijan OR Bangladesh OR Belarus OR Byelarus OR Belorussia OR Belize OR Benin OR Bhutan OR Bolivia OR Bosnia OR Botswana OR Brazil OR Bulgaria OR Burma OR Burkina Faso OR Burundi OR Cabo Verde OR Cape Verde OR Cambodia OR Cameroon OR Central African Republic OR Chad OR China OR Colombia OR Comoros OR Comores OR Comoro OR Congo OR Costa Rica OR Côte d'Ivoire OR Cuba OR Djibouti OR Dominica OR Dominican Republic OR Ecuador OR Egypt OR El Salvador OR Equatorial Guinea OR Eritrea OR Ethiopia OR Fiji OR Gabon OR Gambia OR Gaza OR Georgia OR Georgia Republic OR Ghana OR Grenada OR Grenadines OR Guatemala OR Guinea OR Guinea- Bissau OR Guyana OR Haiti OR Herzegovina OR Hercegovina OR Honduras OR India OR Indonesia OR Iran OR Iraq OR Ivory Coast OR Jamaica OR Jordan OR Kazakhstan OR Kenya OR Kiribati OR Democratic People's Republic of Korea OR Kosovo OR Kyrgyz OR Kirghizia OR Kirghiz OR Kyrgyzstan OR Lao PDR OR Laos OR Lebanon OR Lesotho OR Liberia OR Libya OR Macedonia OR Madagascar OR Malawi OR Malay OR Malaya OR Malaysia OR Maldives OR Mali OR Marshall Islands OR Mauritania OR Mauritius OR Mexico OR Micronesia OR Moldova OR Mongolia OR Montenegro OR Morocco OR Mozambique OR Myanmar OR Namibia OR Nepal OR Nicaragua OR Niger OR Nigeria OR Pakistan OR Palau OR Papua New Guinea OR Paraguay OR Peru OR Philippines OR Principe OR Romania OR Ruanda OR Rwanda OR Samoa OR Sao Tome OR Senegal OR Serbia OR Sierra Leone OR Solomon Islands OR Somalia OR South Africa OR South Sudan OR Sri Lanka OR St Lucia OR St Vincent OR Sudan OR Surinam OR Suriname OR Swaziland OR Syria OR Syrian Arab Republic OR Tajikistan OR Tadzhikistan OR Tajikistan OR Tadzhik OR Tanzania OR Thailand OR Timor OR Togo OR Tonga OR Tunisia OR Turkey OR Turkmen OR Turkmenistan OR Tuvalu OR Uganda OR Ukraine OR Uzbek OR Uzbekistan OR Vanuatu OR Venezuela OR Vietnam OR West Bank OR Yemen OR Zambia OR Zimbabwe
#9	#7 OR #8	

For peer review only

#10	#3 AND #6 AND #9	887 Results on 29 May 2019
Filter	Only Language filter used for PubMed as a preliminary search showed that all recent articles not yet indexed were excluded when filtered for NOT animals and year range	

For peer review only

Preferred Reporting Items for Systematic reviews and Meta-Analyses extension for Scoping Reviews (PRISMA-ScR) Checklist

SECTION	ITEM	PRISMA-ScR CHECKLIST ITEM	REPORTED ON PAGE #
**TITLE**			
Title	1	Identify the report as a scoping review.	1
**ABSTRACT**			
Structured summary	2	Provide a structured summary that includes (as applicable): background, objectives, eligibility criteria, sources of evidence, charting methods, results, and conclusions that relate to the review questions and objectives.	2
**INTRODUCTION**			
Rationale	3	Describe the rationale for the review in the context of what is already known. Explain why the review questions/objectives lend themselves to a scoping review approach.	6
Objectives	4	Provide an explicit statement of the questions and objectives being addressed with reference to their key elements (e.g., population or participants, concepts, and context) or other relevant key elements used to conceptualize the review questions and/or objectives.	7
**METHODS**			
Protocol and registration	5	Indicate whether a review protocol exists; state if and where it can be accessed (e.g., a Web address); and if available, provide registration information, including the registration number.	1
Eligibility criteria	6	Specify characteristics of the sources of evidence used as eligibility criteria (e.g., years considered, language, and publication status), and provide a rationale.	11
Information sources*	7	Describe all information sources in the search (e.g., databases with dates of coverage and contact with authors to identify additional sources), as well as the date the most recent search was executed.	8
Search	8	Present the full electronic search strategy for at least 1 database, including any limits used, such that it could be repeated.	9
Selection of sources of evidence†	9	State the process for selecting sources of evidence (i.e., screening and eligibility) included in the scoping review.	11
Data charting process‡	10	Describe the methods of charting data from the included sources of evidence (e.g., calibrated forms or forms that have been tested by the team before their use, and whether data charting was done independently or in duplicate) and any processes for obtaining and confirming data from investigators.	12
Data items	11	List and define all variables for which data were sought and any assumptions and simplifications made.	12
Critical appraisal of individual sources of evidence§	12	If done, provide a rationale for conducting a critical appraisal of included sources of evidence; describe the methods used and how this information was used in any data synthesis (if appropriate).	13

St. Michael's
Inspired Care.
Inspiring Science.

For peer review only - http://bmjopen.bmj.com/site/about/guidelines.xhtml

SECTION	ITEM	PRISMA-ScR CHECKLIST ITEM	REPORTED ON PAGE #
Synthesis of results	13	Describe the methods of handling and summarizing the data that were charted.	13
**RESULTS**			
Selection of sources of evidence	14	Give numbers of sources of evidence screened, assessed for eligibility, and included in the review, with reasons for exclusions at each stage, ideally using a flow diagram.	Click here to enter text.
Characteristics of sources of evidence	15	For each source of evidence, present characteristics for which data were charted and provide the citations.	Click here to enter text.
Critical appraisal within sources of evidence	16	If done, present data on critical appraisal of included sources of evidence (see item 12).	Click here to enter text.
Results of individual sources of evidence	17	For each included source of evidence, present the relevant data that were charted that relate to the review questions and objectives.	Click here to enter text.
Synthesis of results	18	Summarize and/or present the charting results as they relate to the review questions and objectives.	Click here to enter text.
**DISCUSSION**			
Summary of evidence	19	Summarize the main results (including an overview of concepts, themes, and types of evidence available), link to the review questions and objectives, and consider the relevance to key groups.	Click here to enter text.
Limitations	20	Discuss the limitations of the scoping review process.	3
Conclusions	21	Provide a general interpretation of the results with respect to the review questions and objectives, as well as potential implications and/or next steps.	14
**FUNDING**			
Funding	22	Describe sources of funding for the included sources of evidence, as well as sources of funding for the scoping review. Describe the role of the funders of the scoping review.	14

JBI = Joanna Briggs Institute; PRISMA-ScR = Preferred Reporting Items for Systematic reviews and Meta-Analyses extension for Scoping Reviews.

* Where *sources of evidence* (see second footnote) are compiled from, such as bibliographic databases, social media platforms, and Web sites.

† A more inclusive/heterogeneous term used to account for the different types of evidence or data sources (e.g., quantitative and/or qualitative research, expert opinion, and policy documents) that may be eligible in a scoping review as opposed to only studies. This is not to be confused with *information sources* (see first footnote).

‡ The frameworks by Arksey and O'Malley (6) and Levac and colleagues (7) and the JBI guidance (4, 5) refer to the process of data extraction in a scoping review as data charting.

§ The process of systematically examining research evidence to assess its validity, results, and relevance before using it to inform a decision. This term is used for items 12 and 19 instead of "risk of bias" (which is more applicable to systematic reviews of interventions) to include and acknowledge the various sources of evidence that may be used in a scoping review (e.g., quantitative and/or qualitative research, expert opinion, and policy document).

From: Tricco AC, Lillie E, Zarin W, O'Brien KK, Colquhoun H, Levac D, et al. PRISMA Extension for Scoping Reviews (PRISMA-ScR): Checklist and Explanation. Ann Intern Med. ;169:467–473. doi: 10.7326/M18-0850

BMJ Open

An exploration of recovery of people living with severe mental illness (SMI) in low- and- middle -income countries (LMIC): a scoping review protocol

Journal:	*BMJ Open*
Manuscript ID	bmjopen-2019-032912.R2
Article Type:	Protocol
Date Submitted by the Author:	02-Dec-2019
Complete List of Authors:	Gamieldien, Fadia; University of Cape Town, Psychiatry; University of Cape Town, Health and Rehabilitation Sciences<br>Galvaan, Roshan ; University of Cape Town, Health and Rehabilitation Sciences<br>Myers, Bronwyn; South African Medical Research Council, Alcohol and Drug Abuse Research Unit<br>Sorsdahl, Katherine; University of Cape Town, Psychiatry
**Primary Subject Heading**:	Public health
Secondary Subject Heading:	Mental health, Public health
Keywords:	recovery, severe mental illness, scoping review, low-and-middle- income countries

SCHOLARONE™
Manuscripts

I, the Submitting Author has the right to grant and does grant on behalf of all authors of the Work (as defined in the below author licence), an exclusive licence and/or a non-exclusive licence for contributions from authors who are: i) UK Crown employees; ii) where BMJ has agreed a CC-BY licence shall apply, and/or iii) in accordance with the terms applicable for US Federal Government officers or employees acting as part of their official duties; on a worldwide, perpetual, irrevocable, royalty-free basis to BMJ Publishing Group Ltd ("BMJ") its licensees and where the relevant Journal is co-owned by BMJ to the co-owners of the Journal, to publish the Work in this journal and any other BMJ products and to exploit all rights, as set out in our licence.

The Submitting Author accepts and understands that any supply made under these terms is made by BMJ to the Submitting Author unless you are acting as an employee on behalf of your employer or a postgraduate student of an affiliated institution which is paying any applicable article publishing charge ("APC") for Open Access articles. Where the Submitting Author wishes to make the Work available on an Open Access basis (and intends to pay the relevant APC), the terms of reuse of such Open Access shall be governed by a Creative Commons licence – details of these licences and which Creative Commons licence will apply to this Work are set out in our licence referred to above.

Other than as permitted in any relevant BMJ Author's Self Archiving Policies, I confirm this Work has not been accepted for publication elsewhere, is not being considered for publication elsewhere and does not duplicate material already published. I confirm all authors consent to publication of this Work and authorise the granting of this licence.

For peer review only

An exploration of recovery of people living with severe mental illness (SMI) in low- and- middle -income countries (LMIC): a scoping review protocol

***Fadia Gamieldien*[1], *Roshan Galvaan*[2], *Bronwyn Myers*,[3] *Katherine Sorsdahl* [1]**

[1] Alan J. Flisher Centre for Public Mental Health, Department of Psychiatry and Mental Health, University of Cape Town, Cape Town, South Africa

2. Division of Occupational Therapy, Department of Health and Rehabilitation Sciences, University of Cape Town, Cape Town, South Africa

3 Alcohol, Tobacco and Other Drug Research Unit, South African Medical Research Council and Division of Addiction Psychiatry, Department of Psychiatry and Mental Health, University of Cape Town,Cape Town, South Africa

Corresponding Author: Ms Fadia Gamieldien

Orcid number 0000-0003-2820-6484

Address: Alan J Flisher Centre for Public Mental Health

Department of Psychiatry and Mental Health

University of Cape Town

46 Sawkins Road

Rondebosch, 7700

Cape Town

South Africa

Fadia.Gamieldien@uct.ac.za

+27 82 974 7468

Keywords

Recovery, severe mental illness, low-and-middle-income countries, scoping review

Declaration of Interest: None

For peer review only

ABSTRACT

Introduction: The construct of recovery was conceptualised in high income countries and its applicability in low-and- middle- income countries is underexplored. A scoping review is proposed to synthesise knowledge, review conceptual overlap and map key elements of recovery from severe mental illness in low-and- middle- income countries. We aim to appraise the literature so as to inform future recovery-oriented services that consider the cultural and contextual influences on recovery from severe mental illness.

Methods and analysis: The following electronic databases: MEDLINE via PubMed, SCOPUS (which included contents of Embase), PsycINFO, CINAHL, Africa-Wide Information, PSYCHarticles, Health source: Nursing/Academic Edition, Academic Search Premier and SocINDEX all via the EBSCOHOST platform, the Latin American and Caribbean Health Sciences Literature (LILACS), the Cochrane Centre Register of Controlled Trials) and grey literature sources will be searched between May and December 2019. Eligible studies will be independently screened for inclusion and exclusion by two reviewers using a checklist developed for this purpose. Studies published between January 1993 and November 2019 that focus on recovery from severe mental illness in a low-and -middle- income country will be included. Findings will be compared and discrepancies will be discussed. Unresolved discrepancies will be referred to a third reviewer. All bibliographic data and study characteristics will be extracted and thematically analysed using a tool developed through an iterative process by the research team. Indicators will be classified according to a predefined conceptual framework and categorised and described using qualitative content analysis.

Ethics and dissemination: The review aims at synthesising information from available publications hence it does not require ethical approval. The results will be disseminated through publications, conference presentations and future workshops with stakeholders involved within the recovery paradigm of mental health policy and practice. The scoping review title is registered with the Joanna Briggs Institute.

https://joannabriggs.org/research/registered_titles.aspx

For peer review only

For peer review only - http://bmjopen.bmj.com/site/about/guidelines.xhtml

Strengths and limitations of the study

- This scoping review allows for the systematic synthesis of knowledge on recovery from severe mental illness in to low- and middle-income countries in a rigorous and methodological manner
- This review will highlight the diversity of severe mental illness since there is no agreed upon criteria for including mental illnesses into this category
- Knowledge gaps related to recovery as a multidimensional construct in low- and middle-income countries will be identified
- Only conceptual and theoretical underpinnings of recovery as a construct in LMIC will be discussed without a focus on intervention outcomes.
- Data synthesis will be limited to full text articles available in English only and published between January 1993 and November 2019. Grey literature and unpublished studies in Latin America will be affected by the decision to exclude articles not available in English.

For peer review only

INTRODUCTION

Mental, neurological and substance use (MNS) disorders contribute significantly to the global burden of disease. Whiteford, Ferrari, Degenhardt, Feigin, and Vos (2015) report that MNS disorders account for 10% of disability-adjusted life years (DALYs). However, it has been argued that the true global burden of mental health problems may be underestimated by more than a third and that DALYs are closer to 13%[1]. The lifetime prevalence of severe mental illness (SMIs) ranges between 1% and 4%[2]. Although this is relatively low in comparison to common mental disorders (CMDs), SMIs are more disabling and require complex, long-term interventions for the person and their family[1]. Given the move towards deinstitutionalisation of people with psychiatric disorders, shorter hospital stays are resulting in premature discharge rates and repeated relapse, creating a revolving door phenomenon[3]. These frequent hospital readmissions are costly to the health care system[4].

In low- and- middle- income countries (LMICs) many people living with a SMI are not detected and as a result do not receive the treatment they need[2 5 6]. In a service utilisation review of World Health Organisation member countries, it was found that the lifetime prevalence of mental disorders amongst adults was between 12% to 49%[5]. The review found the treatment gap to be wide across the psychiatric disorders examined. The treatment gap for schizophrenia and non-affective psychosis was 32%. The gap for other disorders was: major depressive disorder, 56%; bipolar disorder, 50%; and alcohol abuse and dependence, 78% [5]. While present in all countries, this treatment gap is greatest in LMICs where there is a need to scale up mental health services[7].

A systematic review on the scaling up of mental health services in LMICs found that in many countries the majority of resources for mental health care are rendered on an inpatient or outpatient basis at large psychiatric hospitals, with little provision of services at primary healthcare facilities or via integration between hospitals and the community[7]. Although there are some nascent programmes to provide services for SMI at primary care facilities, these are generally limited because of a poor integration of mental health into primary health care. Additionally, while the lack of coordination between sectors providing mental health services has seen some countries such as India adopt the District Mental Health Programme, the treatment gap continues in rural areas[8 9].

Despite the drive for deinstitutionalisation and for the rendering of services at decentralised locations, in many countries most of the mental health care budget continues to be allocated to large psychiatric hospitals. In these hospitals, the treatment approach is primarily through medication to alleviate acute clinical symptoms, giving little to no attention to the psychosocial needs of service users[3 7 10]. Unfortunately the sole focus on clinical recovery and the limited

For peer review only - http://bmjopen.bmj.com/site/about/guidelines.xhtml

availability of interventions that focus on rehabilitation and personal recovery further exacerbates the revolving door phenomenon and ongoing treatment gap[3 11].

Recovery oriented approaches offer an extension to medical models of intervention, as they are not solely focused on symptomatic improvement as a marker of clinical recovery[12]. Personal recovery for people with SMI is conceptually distinct from the medical definition of recovery which is clinical in nature and mostly values symptom remission[13 14]. Personal recovery proposes that a person develops new meaning and purpose in their life over the longitudinal course of their illness[12 15]. Psychiatric rehabilitation has highlighted the need to move beyond treating the symptoms of the illness, to treating its functional and disabling consequences as well [16]. Deegan, a mental health professional and person living with schizophrenia, suggested that mental health service users do not "get rehabilitated" by others, but instead they are active participants in their journey to recover new and valued personal meaning and purpose[17].

The recovery model was initiated in high income countries (HICs) with the mental health consumer and survivor movement at a time when having a diagnosis of SMI was highly stigmatised and the prognosis was poor[17]. The idea that people with an SMI cannot recover was challenged by people living with schizophrenia who were leading meaningful lives in their communities after deinstutionalisation, highlighting that recovery was not dependant on long term psychiatric treatment[18 19]. For a person living with an SMI, receiving a diagnosis and psychiatric treatment focussed on clinical recovery is a necessary step towards recovery, but personal recovery is also critical given the cyclical, non-linear, long term course of the illness. In Anthony's seminal work[16], he defined recovery as:

"*a deeply personal, unique process of changing ones' attitude, values, feelings, goals, skills and/or roles. It is a way of living a satisfying, hopeful and contributing life even within the limitations caused by illness*"

Research exploring the meaning of recovery among people living with SMI[20-22] found that recovery means different things to different people, supporting a view that there is no definition of recovery that applies to everyone. A literature review conducted in 2018 explored the meaning of recovery amongst people living with schizophrenia[20]. This review included 17 studies explaining the subjective meaning of recovery. A majority of the included studies were qualitative, with a few taking a quantitative approach to exploring the meaning of recovery. Of the 17 papers only four studies were from LMICs (India (n=2), China and South Africa). The results revealed that recovery is complex, non-linear and perceived as both a process and an outcome. The process was viewed as being individual and it was recommended that more consumer perspectives to inform recovery-oriented interventions which are aligned to

For peer review only - http://bmjopen.bmj.com/site/about/guidelines.xhtml

individuals' personal goals and aspirations were needed. The complexity embedded in viewing recovery as a process and an outcome with multidimensional indicators has been documented by others[23-25]. Further to this, it was recommended that clinicians, caregivers and researchers explore more qualitative and personal narratives of recovery, the tools needed to measure recovery and the development of community-based recovery- oriented services[20].

The aforementioned review was limited to recovery in people living with Schizophrenia and only included four studies from LMIC countries. There is current debate around whether people with SMI in LMICs have a better prognosis and a call to re-examine the realities of living with an SMI amidst social, cultural and economic changes in LMICs [26 27].

Recovery has been viewed as a process or an outcome, with current debates and research seeking to offer conceptual clarity [11]. A systematic review conducted by Leamy et al.[11] led to a proposed conceptual framework for personal recovery in mental illness. The review focused on 97 papers from 13 high income countries and identified characteristics, processes and stages of recovery. While the review included individuals from Black and minority ethnic groups, it did not include any LMICs [11]. The CHIME framework emerged from this review process. This framework conceptualises personal recovery to incorporate the following concepts: **c**onnectedness, **h**ope and optimism about the future, **i**dentity, **m**eaning in life and **e**mpowerment [11]. This highlights that literature on personal recovery is dominated by research conducted in HICs and that there is a gap in understanding recovery from culturally diverse groups[28].

Conducting a scoping review focusing specifically on studies conducted in LMICs will contribute to the literature on recovery in these specific contexts and will be useful for the following reasons. First, little attention has been given to describing recovery as a personal and individual journey from LMIC perspectives. As recovery for the person with an SMI takes place in a social context through a range of relationships, more understanding about how contexts can help or hinder recovery is needed[29].

Second, individual meaning-making within recovery is influenced by social, political, economic and human rights factors but further research is needed to understand the diversity of recovery narratives from the perspectives of different population groups [30]. A systematic review and narrative analysis synthesising descriptions and models of personal recovery was undertaken to inform the development of a conceptual framework to guide recovery oriented research and practice[11]. Studies conducted in HIC specifically the United States featured predominantly in the review which included 97 papers. The review culminated in the emergence of a conceptual framework to describe the characteristics of recovery, the processes of recovery and the stages of recovery. The authors reported a difference in studies which included ethnic

For peer review only

minorities and where more emphasis was placed on stigma, spirituality, culture and the collectivist nature of recovery. They acknowledged that this area is underexplored[11].

Third, in many African cultures an individual's personhood is manifested through their agency in relation to spirituality, society and self[31]. This may impact on the person and how they experience mental illness and recovery. Understanding cultural concepts and the dynamics of interconnectedness means recognising that personhood in LMICs is weaved into how people experience health, illness and recovery[31]. Considering the person as a spiritual and social being provides opportunities to collaborate with caregivers and communities in developing appropriate interventions[31]. In countries that are spiritually diverse, multicultural and home to people of different religious backgrounds it is important to contextualise mental illness and explore health professionals own perspectives on culture, psychiatry and mental health and where mental illness must be contextualised[32 33]. Given the reasons cited previously, further exploration into the recovery journeys of people living with SMI in LMICs and how they view the dimensions of recovery is warranted[20].

A scoping review is proposed as a means of synthesising knowledge on a broadly defined topic and systematically mapping key concepts, theories, evidence and research gaps in the topic area, while still being rigorous and methodological in its approach[34 35]. While systematic reviews and meta-analyses limit their parameters to research trials and quantitative data synthesis, scoping reviews adopt a broader approach to the narrative integration of evidence[36]. We aim to appraise the definitions of recovery and its current utilisation for people with SMI in LMICs. The review findings could be used to inform future practice applications which considers the cultural and contextual factors impacting on recovery[37] from SMI outside of HICs. This scoping review title is registered with the Joanna Briggs Institute (https://joannabriggs.org/research/registered_titles.aspx) although JBI and PROSPERO do not provide registry numbers for scoping reviews at this time[34].

METHODS

A scoping review was deemed as the most suitable method to map existing research on recovery from SMI in LMICs because it allows for the topic to be located, examined, summarised and be presented rapidly and systematically. Additionally it could serve as a precursor to a systematic review [38]. The proposed review will be conducted using the Arksey and O'Malley[35] scoping review methodology and will be reported on using the PRISMA guidelines[39] with an understanding that there is much development as scoping reviews gain popularity especially in health-related topics [34 40].

Arksley and O'Malley [35] offer a methodological framework for conducting a scoping review. Their approach describes six stages: (1) identification of the research question, (2) identification of relevant studies, (3) selection of studies, (4) charting the data, (5) collating, summarising and reporting of results and (6) consultation with stakeholders [35]. The last stage is optional according to Arksley and O'Malley[35] but others have deemed it a necessary step to aide in methodological rigour[41]. Recommendations to enhance the methodology at each stage will be incorporated into this review[34 39 41].

Stage 1: Identifying the research question

In order to guide the search strategy an iterative process of discussion amongst authors occurred in order to develop and refine the research question. The question needed to be clear enough to inform the subsequent stages while still reflecting the scope of inquiry[41]. Given that scoping studies are focused on summarizing the breadth of evidence on a given topic the authors refined the exploratory question to ask the following:

What is known about recovery from severe mental illness in low-and-middle-income countries?

This broad question was developed to allow for a comprehensive review of the literature in order to review and appraise the definition of recovery and how it is understood in mental health research and practice in LMICs. While many have written about this in high income countries[22 42] there is a dearth of such information in LMICs .

Stage 2: Identifying relevant studies

Given the aim of the study is to identify primary published and unpublished studies which will answer the research question, a comprehensive search strategy was developed to aide this. Two health sciences librarians assisted the first author in the iterative process of developing a search strategy including inclusion and exclusion criteria. Decisions were made about time frames, language, search terms and search strategies. Trial searches were run to assess whether relevant literature could be identified using the proposed strategy. A discussion of the identified databases, search strategy and selection criteria follow.

Databases

Electronic databases will be searched to identify studies published between January 1993-November 2019. This period was selected as it covers roughly 25 years in the recovery movement. Personal recovery is a construct that has already been defined by Anthony [16] hence the authors were looking specifically for literature on this construct. Terms used in other disciplines that could also mean recovery were not included as the focus of the search is on recovery as explicitly defined by the recovery movement. Furthermore, recovery

For peer review only - http://bmjopen.bmj.com/site/about/guidelines.xhtml

knowledge and attitudes among health professionals will be included if reported in the selected studies

An expert librarian assisted the first author in identifying information sources that are relevant to the research question. The following databases were selected: MEDLINE via PubMed, SCOPUS (which included contents of Embase), PsycINFO, CINAHL, Africa-Wide Information, PSYCH articles, Health source: Nursing/Academic Edition, Academic Search Premier and SocINDEX all via the EBSCOHOST platform. Additionally, the Latin American and Caribbean Health Sciences Literature (LILACS), the Cochrane Centre Register of Controlled Trials. Grey literature sources will be identified to expand the search and enhance the data sources. Additional sources will be located by means of hand searching reference lists of relevant papers, contacting study authors, searching trial registers, and contacting key personnel involved in recovery-focussed programmes in LMICs. In this way peer reviewed and grey literature from the biomedical sciences, allied health sciences, social sciences and other disciplinary fields will be included.

Search Strategy

Two librarians and the first author developed the eligibility criteria for the scoping review. They identified various definitions of severe mental illness as well as recovery to include in this search strategy. A list of preliminary search terms and filtering methods was developed. The main filtering methods related to the date range of 25 years (January 1993- November 2019). This period was extended so that new publications would not be missed. The search strategy was refined to include MESH terms, filters and Boolean operations to comply with searches across different databases. A search strategy for the PubMed database is included in Supplementary file 1. After the initial search, key words and index terms used across the databases were identified and plotted on a table as indicated below (Table 1).

Table 1 Search strategy terms

Keyword	Alternative
Recovery	Recovery OR recover OR psychosocial rehabilitation OR mental health rehabilitation OR psychiatric rehabilitation OR mental health recovery OR Recovery of function OR Quality of life
Severe mental illness	Severe mental illness OR bipolar OR delusional disorder OR delusional disorders OR major depressive disorder OR major depressive disorders OR schizophrenia OR manic OR manic-depressive OR paranoid disorder OR paranoid disorders OR psychoses OR psychosis OR psychotic disorder OR psychotic disorders OR schizoaffective disorder OR schizoaffective disorders OR Schizophreniform

For peer review only - http://bmjopen.bmj.com/site/about/guidelines.xhtml

	OR serious mental disorder OR serious mental disorders<br>Schizophrenia Spectrum and Other Psychotic Disorders<br>Bipolar and Related Disorders<br>Depressive Disorder
Low- and-middle- income countries	Deprived Countries OR Deprived Population OR Deprived Populations OR Developing Countries OR Developing Country OR Developing Economies OR Developing Economy OR Developing Nation OR Developing Nations OR Developing Population OR Developing Populations OR Developing World OR LAMI Countries OR LAMI Country OR Less Developed Countries OR Less Developed Country OR Less Developed Economies OR Less Developed Nation OR Less Developed Nations OR Less Developed World OR Lesser Developed Countries OR Lesser Developed Nations OR LMIC OR LMICS OR Low GDP OR Low GNP OR Low Gross Domestic OR Low Gross National OR Low Income Countries OR Low Income Country OR Low Income Economies OR Low Income Economy OR Low Income Nations OR Low Income Population OR Low Income Populations OR Lower GDP OR lower gross domestic OR Lower Income Countries OR Lower Income Country OR Lower Income Nations OR Lower Income Population OR Lower Income Populations OR Middle Income Countries OR Middle Income Country OR Middle Income Economies OR Middle Income Nation OR Middle Income Nations OR Middle Income Population OR Middle Income Populations OR Poor Countries OR Poor Country OR Poor Economies OR Poor Economy OR Poor Nation OR Poor Nations OR Poor Population OR Poor Populations OR poor world OR Poorer Countries OR Poorer Economies OR Poorer Economy OR Poorer Nations OR Poorer Population OR Poorer Populations OR Third World OR Transitional Countries OR Transitional Country OR Transitional Economies OR Transitional Economy OR Under Developed Countries OR Under Developed Country OR under developed nations OR Under Developed World OR Under Served Population OR Under Served Populations OR Underdeveloped Countries OR Underdeveloped Country OR underdeveloped economies OR underdeveloped nations OR underdeveloped population OR Underdeveloped World OR Underserved Countries OR Underserved Nations OR Underserved Population OR Underserved Populations<br><br>OR<br>Afghanistan OR Albania OR Algeria OR American Samoa OR Angola OR Armenia OR Azerbaijan OR Bangladesh OR Belarus OR Byelarus OR Belorussia OR Belize OR Benin OR Bhutan OR Bolivia OR Bosnia OR Botswana OR Brazil OR Bulgaria OR Burma OR Burkina Faso OR Burundi OR Cabo Verde OR Cape Verde OR Cambodia OR Cameroon OR Central African Republic OR Chad OR China OR Colombia OR Comoros OR Comores OR Comoro OR Congo OR Costa Rica OR Côte d'Ivoire OR

For peer review only - http://bmjopen.bmj.com/site/about/guidelines.xhtml

	Cuba OR Djibouti OR Dominica OR Dominican Republic OR Ecuador OR Egypt OR El Salvador OR Equatorial Guinea OR Eritrea OR Ethiopia OR Fiji OR Gabon OR Gambia OR Gaza OR Georgia OR Georgia Republic OR Ghana OR Grenada OR Grenadines OR Guatemala OR Guinea OR Guinea- Bissau OR Guyana OR Haiti OR Herzegovina OR Hercegovina OR Honduras OR India OR Indonesia OR Iran OR Iraq OR Jamaica OR Jordan OR Kazakhstan OR Kenya OR Kiribati OR Democratic People's Republic of Korea OR Kosovo OR Kyrgyz OR Kirghizia OR Kirghiz OR Kyrgyzstan OR Lao PDR OR Laos OR Lebanon OR Lesotho OR Liberia OR Libya OR Macedonia OR Madagascar OR Malawi OR Malay OR Malaya OR Malaysia OR Maldives OR Mali OR Marshall Islands OR Mauritania OR Mauritius OR Mexico OR Micronesia OR Moldova OR Mongolia OR Montenegro OR Morocco OR Mozambique OR Myanmar OR Namibia OR Nepal OR Nicaragua OR Niger OR Nigeria OR Pakistan OR Palau OR Panama OR Papua New Guinea OR Paraguay OR Peru OR Philippines OR Principe OR Romania OR Ruanda OR Rwanda OR Samoa OR Sao Tome OR Senegal OR Serbia OR Sierra Leone OR Solomon Islands OR Somalia OR South Africa OR South Sudan OR Sri Lanka OR St Lucia OR St Vincent OR Sudan OR Surinam OR Suriname OR Swaziland OR Syria OR Syrian Arab Republic OR Tajikistan OR Tadzhikistan OR Tajikistan OR Tadzhik OR Tanzania OR Thailand OR Timor OR Togo OR Tonga OR Tunisia OR Turkey OR Turkmen OR Turkmenistan OR Tuvalu OR Uganda OR Ukraine OR Uzbek OR Uzbekistan OR Vanuatu OR Venezuela OR Vietnam OR West Bank OR Yemen OR Zambia OR Zimbabwe

There are no universally accepted guidelines to operationalise the concept of recovery and there is no measure of a gold standard of recovery[43]. The CHIME framework is one proposed guide for the development of recovery measures and identifying recovery outcomes for consideration in clinical practice, but this is limited to HICs. In this review, the key concept is personal recovery as defined by the seminal work of Anthony [16]. In the search strategy, the concept of recovery will be explored through the MeSH terms: Psychiatric Rehabilitation; Mental Health Recovery and Recovery of Function as well as through the free text terms: Recovery OR recover OR psychosocial rehabilitation OR mental health rehabilitation OR psychiatric rehabilitation.

For peer review only

Selection criteria

Articles will be included in the review if they meet the following inclusion and exclusion criteria (Table 2).

Table 2 Inclusion and exclusion criteria

Criteria for inclusion	Criteria for exclusion
• Published between January 1993-November 2019<br>• Qualitative and quantitative empirical (i.e. primary research) study designs<br>• Theoretical literature on the construct of recovery<br>• Studies will be included if they have been conducted in LMICs<br>• Peer reviewed scientific literature<br>• Literature focussing on the population of interest; that is adults with SMI in LMICs will be included. The diagnosis of SMI includes schizophrenia spectrum disorders, schizoaffective disorders, bipolar affective disorders, major depressive disorder or related psychotic disorders (schizophreniform, delusional disorder, substance induced psychotic disorder or disorders not otherwise specified) as well as those with a comorbid substance use disorder. Studies and reports will be excluded if they do not include the keywords or alternatives as outlined in table 1<br>• Explicit mention of severe mental illness and recovery in LMICS	• The review will be limited to full text articles written in English. However, the authors acknowledge that this practical decision could mean that potentially relevant papers are missed<br>• Conference abstracts will be excluded although they will be reviewed to see if full reports were published<br>• Children will be excluded as the age range for inclusion is 18 years and older<br>• Duplicate articles from the same study will be excluded<br>• Policy documents will be excluded although they will be reviewed to access research in LMICs that they might have referenced<br>• Studies that occurred only in high income countries, without a LMIC focus will be excluded<br>• Studies must be conducted in LMIC and not in HIC with participants from LMICs

For peer review only - http://bmjopen.bmj.com/site/about/guidelines.xhtml

Stage 3: Study selection

The scoping review guideline for identifying and selecting studies informs article selection and data extraction [35]. Two screening levels are proposed: a title and abstract review and a full-text review. For the first level of screening, two authors (FG, ZS) will independently screen the title and abstracts of all retrieved citations against a few select inclusion criteria. Any articles that seem relevant by either or both reviewers will be included in the full-text review. Next, these two reviewers will independently screen the full text articles to assess whether they meet the inclusion and exclusion criteria. At both levels of screening, inter-rater agreement will be assessed by calculating Cohen's Kappa. Any disagreement on study selection will be resolved by discussion with a third rater, who will be a senior member of the research team.

Quality appraisal

The Mixed Methods Appraisal Tool (MMAT) version 2018[44] and the Critical Appraisal Skills Programme (CASP) Qualitative checklist will used to assess the quality of included studies although this is not required for a scoping review nor will it influence the inclusion of the study.

Using the aforementioned criteria, the number of studies identified and selected for inclusion in the scoping review will be reported on. A narrative description[34] will accompany the search decision flow chart[39]. The flow chart will show the results from the search, the removal of duplicates, study selection, full retrieval numbers, additional searches from reference lists and a final summary of decisions related to the search decision and an explanation of the review decision process[34].

Stage 4: Charting the data

Next, the two reviewers (FG and ZS) will independently extract data from the identified articles and chart it[35]. A draft data charting form will be created in the MS Excel programme to facilitate this process[35]. This form will be pre-tested and an iterative process of refining the form will be undertaken as the review commences to ensure that all relevant information is extracted[34].

The form will allow the reviewers to confirm study relevance, record study characteristics and extract information relevant to the review question. The following information will be extracted from the articles: 1) study title; 2) author; 3) study population; 4) participant characteristics; 5) research question; 6) study methodology; 7) study description; 8) intervention type (if applicable); 9) intervention duration (if applicable); 10) outcome measures; 11 summary of findings; 12) definitions of key concepts (recovery and severe mental illness); 13) conceptual links identified; 14) practice implications and; 15) recommendations for further development.

For peer review only - http://bmjopen.bmj.com/site/about/guidelines.xhtml

While charting the data it is important to maintain flexibility around including emerging categories and consulting with the team as this occurs. The final version of the charting form along with definitions of included items will be provided as an appendix to the review. This process will facilitate the timeous completion of the review while also maintaining research rigour.

Stage 5: Collating, summarising and reporting the results

All results will be collated, summarised and reported on in order to provide a comprehensive and thorough overview of all the reviewed literature. The authors plan to follow an iterative process and not pre-empt or fit findings into what has already been written about recovery in HICs. This will allow new understandings to be uncovered. While the CHIME framework is considered to offer a comprehensive overview of the process of recovery, it has only been used in HICs with predominantly White populations so its applicability with ethnically and culturally diverse groups is unknown[11 28]. The role of non-government organisations (NGOs) and traditional healers in providing services that facilitate recovery for people living with SMIs will be examined if this arises from the scoping review.

A three-step approach[41] of analysing the data, reporting results and applying meaning to results will be followed to provide methodological rigour. Collating and summarising results will include a summary of the types of studies conducted (including the quantity, types of study design, populations, setting) as well as a thematic analysis of the results. More specifically, we will code how studies describe the concept of recovery from SMI and how it is defined and understood in LMICs. The framework used to report the findings will be developed iteratively through the examination of results. Additionally, the authors will report on the broader implications of the review in terms of research, policy and practice.

Stage 6: Consultation

While Arksey and O'Malley[35] view the consultation stage as an optional step, Levac[41] views it as a requirement to ensure methodological rigour. During each stage of the review process we will consult with an existing stakeholder advisory committee. This advisory committee consists of the research team and key stakeholders including mental health service providers and service users from within the public health and non-profit organisation (NPO) sectors. This committee has been constituted from the onset of the project and some members are part of a pre-existing group for public mental health specialists. The advisory committee will be consulted to get their views on the relevance of the review, guidance for accessing grey literature and their perspectives on the data extraction process and preliminary findings.

For peer review only - http://bmjopen.bmj.com/site/about/guidelines.xhtml

Including an advisory committee in the review is informed by a pragmatic approach[45]where the research is treated as a human experience with the understanding that this consultation process is likely to enhance the relevance of this review. It will also support collaborative efforts to facilitate consumer participation, public involvement, access to information and transfer of knowledge[41].

Patient and public involvement

There is no patient involvement in the protocol development aspect of the study. Service users forming part of the advisory committee referred to in Stage 6 will be consulted on findings and dissemination of results.

Ethics and dissemination

Since the scoping review methodology aims at synthesising information from available publications, this study does not require ethical approval. Reviews of primary research allows for the mapping of evidence in fields where the body of literature has not yet been comprehensively reviewed or where working definitions and concepts are still developing. The findings will be made available in different formats to facilitate its dissemination. An article reporting on the results will be submitted for publication in a peer -reviewed journal. It will also be presented at relevant conferences and stakeholder engagements involved in mental health policy and practice. These stakeholders include MHSUs, academics, policy makers, researcher and clinicians. Findings will also be shared via newsletters, policy briefs and social media forums.

Author Contributions

This protocol was developed by the intellectual contributions of all the authors. All authors were involved in developing the review question and conceptualising the approach. FG developed and tested the search terms in consultation with a subject librarian. FG and KS developed the data extraction guideline, and this was revised based on feedback from the other authors. RG, KS and BM provided substantial critique and review of the draft protocol.

Funding

This work was supported by the DELTAS Africa Initiative [DEL-15-01]. The DELTAS Africa Initiative is an independent funding scheme of the African Academy of Sciences (AAS)'s Alliance for Accelerating Excellence in Science in Africa (AESA) and supported by the New Partnership for Africa's Development Planning and Coordinating Agency (NEPAD Agency) with funding from the Wellcome Trust [DEL-15-01] and the UK government. The views expressed in this publication are those of the author(s) and not necessarily those of AAS,

For peer review only - http://bmjopen.bmj.com/site/about/guidelines.xhtml

NEPAD Agency, WellcomeTrust or the UK government. BM is funded through the South African Medical Research Council.

For peer review only

References

1. Vigo D, Thornicroft G, Atun R. Estimating the true global burden of mental illness. *The Lancet Psychiatry* 2016;3(2):171-78. doi: 10.1016/S2215-0366(15)00505-2
2. Kessler RC, Berglund PA, Bruce ML, et al. The prevalence and correlates of untreated serious mental illness. *Health Serv Res* 2001;36(6 Pt 1):987.
3. Petersen I, Lund C, Stein DJ. Optimizing mental health services in low-income and middle-income countries. *Current opinion in psychiatry* 2011;24(4):318-23. doi: 10.1097/YCO.0b013e3283477afb
4. Sfetcu R, Musat S, Haaramo P, et al. Overview of post-discharge predictors for psychiatric re-hospitalisations: a systematic review of the literature. *BMC Psychiatry* 2017;17(1) doi: 10.1186/s12888-017-1386-z
5. Kohn R, Saxena S, Levav I, et al. The treatment gap in mental health care. *World Health Organization Bulletin of the World Health Organization* 2004;82(11):858-66. doi: 10.1590/S0042-96862004001100011
6. Demyttenaere K, Bruffaerts R, Posada-Villa J, et al. Prevalence, severity, and unmet need for treatment of mental disorders in the World Health Organization World Mental Health Surveys. *Jama* 2004;291(21):2581-90. doi: 10.1001/jama.291.21.2581 [published Online First: 2004/06/03]
7. Eaton J, McCay L, Semrau M, et al. Scale up of services for mental health in low-income and middle-income countries. *The Lancet* 2011;378(9802):1592-603. doi: https://doi.org/10.1016/S0140-6736(11)60891-X
8. Thornicroft G, Cooper S, Bortel TV, et al. Capacity Building in Global Mental Health Research. *Harv Rev Psychiatry* 2012;20(1):13-24. doi: 10.3109/10673229.2012.649117
9. Kumar A. Mental health services in rural India: challenges and prospects. *Health (N Y)* 2011;3(12):757-61.
10. Lund C, Oosthuizen P, Flisher AJ, et al. Pathways to Inpatient Mental Health Care Among People With Schizophrenia Spectrum Disorders in South Africa. *Psychiatr Serv* 2010;61(3):235-40. doi: 10.1176/ps.2010.61.3.235
11. Leamy M, Bird V, Le Boutillier C, et al. Conceptual framework for personal recovery in mental health: systematic review and narrative synthesis. *Br J Psychiatry* 2011;199(6):445-52. doi: 10.1192/bjp.bp.110.083733
12. Slade M. Personal recovery and mental illness: A guide for mental health professionals: Cambridge University Press 2009.
13. Slade M. Mental illness and well-being: the central importance of positive psychology and recovery approaches. *BMC Health Serv Res* 2010;10:26-26. doi: 10.1186/1472-6963-10-26
14. Myers NL, Myers NL. Culture, stress and recovery from schizophrenia: lessons from the field for global mental health. *Cult Med Psychiatry* 2010;34(3):500-28. doi: 10.1007/s11013-010-9186-7
15. Tew J, Ramon S, Slade M, et al. Social factors and recovery from mental health difficulties: a review of the evidence. *The British Journal of Social Work* 2012;42(3):443-60.
16. Anthony WA. Recovery from mental illness: the guiding vision of the mental health service system in the 1990s. *Psychosocial rehabilitation journal* 1993;16(4):11.
17. Deegan PE. Recovery: The lived experience of rehabilitation. *Psychosocial rehabilitation journal* 1988;11(4):11.
18. Webb L. The recovery model and complex health needs: what health psychology can learn from mental health and substance misuse service provision. *J Health Psychol* 2012;17(5):731-41. doi: 10.1177/1359105311425276
19. Farkas M. The vision of recovery today: what it is and what it means for services. *World Psychiatric Association (WPA)* 2007;6(2):68.

For peer review only

20. Mathew ST, Nirmala BP, Kommu JVS. Meaning of Recovery Among Persons with Schizophrenia: A Literature Review. *Journal of Psychosocial Rehabilitation and Mental Health* 2018;5(1):73-81. doi: 10.1007/s40737-018-0111-7
21. Kelly M, Lamont S, Brunero S. An Occupational Perspective of the Recovery Journey in Mental Health. *Br J Occup Ther* 2010;73(3):129-35. doi: 10.4276/030802210x12682330090532
22. Cabassa LJ, Nicasio A, Whitley R. Picturing Recovery: A Photovoice Exploration of Recovery Dimensions Among People With Serious Mental Illness. *Psychiatric services (Washington, DC)* 2013;64(9):10.1176/appi.ps.201200503. doi: 10.1176/appi.ps.201200503
23. Aldersey HM, Adeponle AB, Whitley R. Diverse Approaches to Recovery from Severe Mental Illness. The Palgrave Handbook of Sociocultural Perspectives on Global Mental Health2017:109-27.
24. Frost BG, Tirupati S, Johnston S, et al. An Integrated Recovery-oriented Model (IRM) for mental health services: evolution and challenges. *BMC Psychiatry* 2017;17:22. doi: 10.1186/s12888-016-1164-3
25. Lysaker PH, Hamm JA, Hasson-Ohayon I, et al. Promoting recovery from severe mental illness: Implications from research on metacognition and metacognitive reflection and insight therapy. *World Journal of Psychiatry* 2018;8(1):1-11. doi: 10.5498/wjp.v8.i1.1 [published Online First: 2018/03/24]
26. Cohen A, Patel V, Thara R, et al. Questioning an axiom: better prognosis for schizophrenia in the developing world? *Schizophr Bull* 2008;34(2):229-44. doi: 10.1093/schbul/sbm105 [published Online First: 2007/10/02]
27. Patel V, Saxena S, Lund C, et al. The Lancet Commission on global mental health and sustainable development. *The Lancet* 2018;392(10157):1553-98.
28. Brijnath B. Applying the CHIME recovery framework in two culturally diverse Australian communities: Qualitative results. *Int J Soc Psychiatry* 2015;61(7):660-67. doi: 10.1177/0020764015573084
29. Schön U-K, Denhov A, Topor A. Social relationships as a decisive factor in recovering from severe mental illness. *Int J Soc Psychiatry* 2009;55(4):336-47.
30. Llewellyn-Beardsley J, Rennick-Egglestone S, Callard F, et al. Characteristics of mental health recovery narratives: Systematic review and narrative synthesis.(Research Article): Public Library of Science, 2019:e0214678.
31. Kpanake L. Cultural concepts of the person and mental health in Africa. *Transcultural Psychiatry* 2018;55(2):198-218. doi: 10.1177/1363461517749435
32. Janse van Rensburg ABR. South African Society of Psychiatrists guidelines for the integration of spirituality in the approach to psychiatric practice : guideline. 2014;20(4):133-39. doi: 10.4102/sajpsychiatry.v20i4.593
33. Swartz L, Foster D. Images of culture and mental illness : South African psychiatric approaches. *Social Dynamics* 1984;10(1):17-25. doi: 10.1080/02533958408458354
34. Peters MD, Godfrey CM, Khalil H, et al. Guidance for conducting systematic scoping reviews. *International journal of evidence-based healthcare* 2015;13(3):141-46.
35. Arksey H, O'Malley L. Scoping studies: towards a methodological framework. *International Journal of Social Research Methodology* 2005;8(1):19-32. doi: 10.1080/1364557032000119616
36. Dijkers M. What is a scoping review?. KT Update, 4 (1), 2015.
37. Myers NL. Culture, stress and recovery from schizophrenia: lessons from the field for global mental health. *Cult Med Psychiatry* 2010;34(3):500-28. doi: 10.1007/s11013-010-9186-7
38. McKinstry C, Brown T, Gustafsson L. Scoping reviews in occupational therapy: the what, why, and how to. *Aust Occup Ther J* 2014;61(2):58-66. doi: 10.1111/1440-1630.12080
39. Tricco AC, Lillie E, Zarin W, et al. Prisma extension for scoping reviews (prisma-scr): Checklist and explanation. *Ann Intern Med* 2018;169(7):467-73. doi: 10.7326/M18-0850

For peer review only

40. Pham MT, Rajić A, Greig JD, et al. A scoping review of scoping reviews: advancing the approach and enhancing the consistency. *Research Synthesis Methods* 2014;5(4):371-85. doi: doi:10.1002/jrsm.1123
41. Levac D, Colquhoun H, O'Brien KK. Scoping studies: advancing the methodology. *Implementation science* 2010;5(1):69.
42. Slade M, Longden E. Empirical evidence about recovery and mental health. *BMC Psychiatry* 2015;15:285. doi: 10.1186/s12888-015-0678-4
43. Shanks V, Williams J, Leamy M, et al. Measures of personal recovery: a systematic review. *Psychiatr Serv* 2013;64(10):974-80.
44. Hong QN, Pluye P, Fàbregues S, et al. Mixed methods appraisal tool (MMAT), version 2018. *IC Canadian Intellectual Property Office, Industry Canada* 2018
45. Morgan DL. Pragmatism as a Paradigm for Social Research. *Qualitative Inquiry* 2014;20(8):1045-53. doi: 10.1177/1077800413513733

For peer review only

For peer review only - http://bmjopen.bmj.com/site/about/guidelines.xhtml

Supplementary file 1

PubMed Search

Population, Concept, Context (PCC)

**Population:**		
#1	MeSH terms:	Schizophrenia Spectrum and Other Psychotic Disorders<br>Bipolar and Related Disorders<br>Depressive disorder, Major
#2	Free text:	Severe mental illness OR bipolar OR delusional disorder OR delusional disorders OR major depressive disorder OR major depressive disorders OR schizophrenia OR manic OR manic-depressive OR paranoid disorder OR paranoid disorders OR psychoses OR psychosis OR psychotic disorder OR psychotic disorders OR schizoaffective disorder OR schizoaffective disorders OR Schizophreniform OR serious mental disorder OR serious mental disorders
#3	#1 OR #2	
**Concept:**		
#4	MeSH terms:	Psychiatric Rehabilitation<br>Mental Health Recovery<br>Recovery of Function
#5	Free text:	Recovery OR recover OR psychosocial rehabilitation OR mental health rehabilitation OR psychiatric rehabilitation
#6	#4 OR #5	
**Context: Low and Middle Income Countries (LMICS)**		
#7	Free text:	Deprived Countries OR Deprived Population OR Deprived Populations OR Developing Countries OR Developing Country OR Developing Economies OR Developing Economy OR Developing Nation OR Developing Nations OR Developing Population OR Developing Populations OR Developing World OR LAMI Countries OR LAMI Country OR Less Developed Countries OR Less Developed Country OR Less Developed Economies OR Less Developed Nation OR Less Developed Nations OR Less Developed World OR Lesser Developed Countries OR Lesser Developed Nations OR LMIC OR LMICS OR Low GDP OR Low GNP OR Low Gross Domestic OR Low Gross National OR Low Income Countries OR Low Income Country OR Low Income Economies OR Low Income Economy OR Low Income Nations OR Low Income Population OR Low Income Populations OR Lower GDP OR lower gross domestic OR Lower Income Countries OR Lower Income Country OR Lower Income Nations OR Lower Income Population OR Lower Income Populations OR Middle Income Countries OR Middle Income Country OR Middle Income Economies OR Middle Income Nation OR Middle Income Nations OR Middle Income Population OR Middle Income Populations OR Poor Countries OR Poor Country OR Poor Economies OR Poor Economy OR Poor Nation OR Poor Nations OR Poor Population OR Poor Populations OR poor world OR Poorer Countries OR Poorer Economies OR Poorer Economy OR Poorer Nations OR Poorer Population OR Poorer Populations OR Third World OR Transitional Countries OR Transitional Country OR Transitional Economies OR Transitional Economy OR Under Developed Countries OR Under Developed Country OR under developed nations OR Under Developed World OR Under Served Population OR Under Served Populations OR Underdeveloped Countries OR Underdeveloped Country OR

For peer review only - http://bmjopen.bmj.com/site/about/guidelines.xhtml

		underdeveloped economies OR underdeveloped nations OR underdeveloped population OR Underdeveloped World OR Underserved Countries OR Underserved Nations OR Underserved Population OR Underserved Populations
#8	Free text:	Afghanistan OR Albania OR Algeria OR American Samoa OR Angola OR Armenia OR Azerbaijan OR Bangladesh OR Belarus OR Byelarus OR Belorussia OR Belize OR Benin OR Bhutan OR Bolivia OR Bosnia OR Botswana OR Brazil OR Bulgaria OR Burma OR Burkina Faso OR Burundi OR Cabo Verde OR Cape Verde OR Cambodia OR Cameroon OR Central African Republic OR Chad OR China OR Colombia OR Comoros OR Comores OR Comoro OR Congo OR Costa Rica OR Côte d'Ivoire OR Cuba OR Djibouti OR Dominica OR Dominican Republic OR Ecuador OR Egypt OR El Salvador OR Equatorial Guinea OR Eritrea OR Ethiopia OR Fiji OR Gabon OR Gambia OR Gaza OR Georgia OR Georgia Republic OR Ghana OR Grenada OR Grenadines OR Guatemala OR Guinea OR Guinea- Bissau OR Guyana OR Haiti OR Herzegovina OR Hercegovina OR Honduras OR India OR Indonesia OR Iran OR Iraq OR Ivory Coast OR Jamaica OR Jordan OR Kazakhstan OR Kenya OR Kiribati OR Democratic People's Republic of Korea OR Kosovo OR Kyrgyz OR Kirghizia OR Kirghiz OR Kyrgyzstan OR Lao PDR OR Laos OR Lebanon OR Lesotho OR Liberia OR Libya OR Macedonia OR Madagascar OR Malawi OR Malay OR Malaya OR Malaysia OR Maldives OR Mali OR Marshall Islands OR Mauritania OR Mauritius OR Mexico OR Micronesia OR Moldova OR Mongolia OR Montenegro OR Morocco OR Mozambique OR Myanmar OR Namibia OR Nepal OR Nicaragua OR Niger OR Nigeria OR Pakistan OR Palau OR Papua New Guinea OR Paraguay OR Peru OR Philippines OR Principe OR Romania OR Ruanda OR Rwanda OR Samoa OR Sao Tome OR Senegal OR Serbia OR Sierra Leone OR Solomon Islands OR Somalia OR South Africa OR South Sudan OR Sri Lanka OR St Lucia OR St Vincent OR Sudan OR Surinam OR Suriname OR Swaziland OR Syria OR Syrian Arab Republic OR Tajikistan OR Tadzhikistan OR Tajikistan OR Tadzhik OR Tanzania OR Thailand OR Timor OR Togo OR Tonga OR Tunisia OR Turkey OR Turkmen OR Turkmenistan OR Tuvalu OR Uganda OR Ukraine OR Uzbek OR Uzbekistan OR Vanuatu OR Venezuela OR Vietnam OR West Bank OR Yemen OR Zambia OR Zimbabwe
#9	#7 OR #8	
#10	#3 AND #6 AND #9	887 Results (290519)
Filter	Only language filter used for PubMed as a preliminary search showed that all recent articles not yet indexed were excluded when filtered for NOT animals and year range	

For peer review only

Preferred Reporting Items for Systematic reviews and Meta-Analyses extension for Scoping Reviews (PRISMA-ScR) Checklist

SECTION	ITEM	PRISMA-ScR CHECKLIST ITEM	REPORTED ON PAGE #
**TITLE**			
Title	1	Identify the report as a scoping review.	1
**ABSTRACT**			
Structured summary	2	Provide a structured summary that includes (as applicable): background, objectives, eligibility criteria, sources of evidence, charting methods, results, and conclusions that relate to the review questions and objectives.	2
**INTRODUCTION**			
Rationale	3	Describe the rationale for the review in the context of what is already known. Explain why the review questions/objectives lend themselves to a scoping review approach.	5
Objectives	4	Provide an explicit statement of the questions and objectives being addressed with reference to their key elements (e.g., population or participants, concepts, and context) or other relevant key elements used to conceptualize the review questions and/or objectives.	8
**METHODS**			
Protocol and registration	5	Indicate whether a review protocol exists; state if and where it can be accessed (e.g., a Web address); and if available, provide registration information, including the registration number.	2
Eligibility criteria	6	Specify characteristics of the sources of evidence used as eligibility criteria (e.g., years considered, language, and publication status), and provide a rationale.	11
Information sources*	7	Describe all information sources in the search (e.g., databases with dates of coverage and contact with authors to identify additional sources), as well as the date the most recent search was executed.	9
Search	8	Present the full electronic search strategy for at least 1 database, including any limits used, such that it could be repeated.	9
Selection of sources of evidence†	9	State the process for selecting sources of evidence (i.e., screening and eligibility) included in the scoping review.	11
Data charting process‡	10	Describe the methods of charting data from the included sources of evidence (e.g., calibrated forms or forms that have been tested by the team before their use, and whether data charting was done independently or in duplicate) and any processes for obtaining and confirming data from investigators.	13
Data items	11	List and define all variables for which data were sought and any assumptions and simplifications made.	13
Critical appraisal of individual sources of evidence§	12	If done, provide a rationale for conducting a critical appraisal of included sources of evidence; describe the methods used and how this information was used in any data synthesis (if appropriate).	13

For peer review only - http://bmjopen.bmj.com/site/about/guidelines.xhtml

SECTION	ITEM	PRISMA-ScR CHECKLIST ITEM	REPORTED ON PAGE #
Synthesis of results	13	Describe the methods of handling and summarizing the data that were charted.	14
**RESULTS**			
Selection of sources of evidence	14	Give numbers of sources of evidence screened, assessed for eligibility, and included in the review, with reasons for exclusions at each stage, ideally using a flow diagram.	Click here to enter text.
Characteristics of sources of evidence	15	For each source of evidence, present characteristics for which data were charted and provide the citations.	Click here to enter text.
Critical appraisal within sources of evidence	16	If done, present data on critical appraisal of included sources of evidence (see item 12).	Click here to enter text.
Results of individual sources of evidence	17	For each included source of evidence, present the relevant data that were charted that relate to the review questions and objectives.	Click here to enter text.
Synthesis of results	18	Summarize and/or present the charting results as they relate to the review questions and objectives.	Click here to enter text.
**DISCUSSION**			
Summary of evidence	19	Summarize the main results (including an overview of concepts, themes, and types of evidence available), link to the review questions and objectives, and consider the relevance to key groups.	Click here to enter text.
Limitations	20	Discuss the limitations of the scoping review process.	3
Conclusions	21	Provide a general interpretation of the results with respect to the review questions and objectives, as well as potential implications and/or next steps.	N/A
**FUNDING**			
Funding	22	Describe sources of funding for the included sources of evidence, as well as sources of funding for the scoping review. Describe the role of the funders of the scoping review.	15

JBI = Joanna Briggs Institute; PRISMA-ScR = Preferred Reporting Items for Systematic reviews and Meta-Analyses extension for Scoping Reviews.

* Where *sources of evidence* (see second footnote) are compiled from, such as bibliographic databases, social media platforms, and Web sites.

† A more inclusive/heterogeneous term used to account for the different types of evidence or data sources (e.g., quantitative and/or qualitative research, expert opinion, and policy documents) that may be eligible in a scoping review as opposed to only studies. This is not to be confused with *information sources* (see first footnote).

‡ The frameworks by Arksey and O'Malley (6) and Levac and colleagues (7) and the JBI guidance (4, 5) refer to the process of data extraction in a scoping review as data charting.

§ The process of systematically examining research evidence to assess its validity, results, and relevance before using it to inform a decision. This term is used for items 12 and 19 instead of "risk of bias" (which is more applicable to systematic reviews of interventions) to include and acknowledge the various sources of evidence that may be used in a scoping review (e.g., quantitative and/or qualitative research, expert opinion, and policy document).

From: Tricco AC, Lillie E, Zarin W, O'Brien KK, Colquhoun H, Levac D, et al. PRISMA Extension for Scoping Reviews (PRISMA-ScR): Checklist and Explanation. Ann Intern Med. ;169:467–473. doi: 10.7326/M18-0850

For peer review only - http://bmjopen.bmj.com/site/about/guidelines.xhtml

BMJ Open

An exploration of recovery of people living with severe mental illness (SMI) in low- and- middle -income countries (LMIC): a scoping review protocol

Journal:	*BMJ Open*
Manuscript ID	bmjopen-2019-032912.R3
Article Type:	Protocol
Date Submitted by the Author:	24-Dec-2019
Complete List of Authors:	Gamieldien, Fadia; University of Cape Town, Psychiatry; University of Cape Town, Health and Rehabilitation Sciences<br>Galvaan, Roshan ; University of Cape Town, Health and Rehabilitation Sciences<br>Myers, Bronwyn; South African Medical Research Council, Alcohol and Drug Abuse Research Unit<br>Sorsdahl, Katherine; University of Cape Town, Psychiatry
**Primary Subject Heading**:	Public health
Secondary Subject Heading:	Mental health, Public health
Keywords:	recovery, severe mental illness, scoping review, low-and-middle- income countries

SCHOLARONE™
Manuscripts

I, the Submitting Author has the right to grant and does grant on behalf of all authors of the Work (as defined in the below author licence), an exclusive licence and/or a non-exclusive licence for contributions from authors who are: i) UK Crown employees; ii) where BMJ has agreed a CC-BY licence shall apply, and/or iii) in accordance with the terms applicable for US Federal Government officers or employees acting as part of their official duties; on a worldwide, perpetual, irrevocable, royalty-free basis to BMJ Publishing Group Ltd ("BMJ") its licensees and where the relevant Journal is co-owned by BMJ to the co-owners of the Journal, to publish the Work in this journal and any other BMJ products and to exploit all rights, as set out in our licence.

The Submitting Author accepts and understands that any supply made under these terms is made by BMJ to the Submitting Author unless you are acting as an employee on behalf of your employer or a postgraduate student of an affiliated institution which is paying any applicable article publishing charge ("APC") for Open Access articles. Where the Submitting Author wishes to make the Work available on an Open Access basis (and intends to pay the relevant APC), the terms of reuse of such Open Access shall be governed by a Creative Commons licence – details of these licences and which Creative Commons licence will apply to this Work are set out in our licence referred to above.

Other than as permitted in any relevant BMJ Author's Self Archiving Policies, I confirm this Work has not been accepted for publication elsewhere, is not being considered for publication elsewhere and does not duplicate material already published. I confirm all authors consent to publication of this Work and authorise the granting of this licence.

For peer review only

An exploration of recovery of people living with severe mental illness (SMI) in low- and- middle -income countries (LMIC): a scoping review protocol

Fadia Gamieldien[1], *Roshan Galvaan[2], Bronwyn Myers*,[3] ***Katherine Sorsdahl [1]***

[1] Alan J. Flisher Centre for Public Mental Health, Department of Psychiatry and Mental Health, University of Cape Town, Cape Town, South Africa

2. Division of Occupational Therapy, Department of Health and Rehabilitation Sciences, University of Cape Town, Cape Town, South Africa

3 Alcohol, Tobacco and Other Drug Research Unit, South African Medical Research Council and Division of Addiction Psychiatry, Department of Psychiatry and Mental Health, University of Cape Town,Cape Town, South Africa

Corresponding Author: Ms Fadia Gamieldien

Orcid number 0000-0003-2820-6484

Address: Alan J Flisher Centre for Public Mental Health

Department of Psychiatry and Mental Health

University of Cape Town

46 Sawkins Road

Rondebosch, 7700

Cape Town

South Africa

Fadia.Gamieldien@uct.ac.za

+27 82 974 7468

Keywords

Recovery, severe mental illness, low-and-middle-income countries, scoping review

Declaration of Interest: None

For peer review only

ABSTRACT

Introduction: The construct of recovery was conceptualised in high income countries and its applicability in low-and- middle- income countries is underexplored. A scoping review is proposed to synthesise knowledge, review conceptual overlap and map key elements of recovery from severe mental illness in low-and- middle- income countries. We aim to appraise the literature so as to inform future recovery-oriented services that consider the cultural and contextual influences on recovery from severe mental illness.

Methods and analysis: The following electronic databases: MEDLINE via PubMed, SCOPUS (which included contents of Embase), PsycINFO, CINAHL, Africa-Wide Information, PSYCHarticles, Health source: Nursing/Academic Edition, Academic Search Premier and SocINDEX all via the EBSCOHOST platform, the Latin American and Caribbean Health Sciences Literature (LILACS), the Cochrane Centre Register of Controlled Trials) and grey literature sources will be searched between May and December 2019. Eligible studies will be independently screened for inclusion and exclusion by two reviewers using a checklist developed for this purpose. Studies published between January 1993 and November 2019 that focus on recovery from severe mental illness in a low-and -middle- income country will be included. Findings will be compared and discrepancies will be discussed. Unresolved discrepancies will be referred to a third reviewer. All bibliographic data and study characteristics will be extracted and thematically analysed using a tool developed through an iterative process by the research team. Indicators will be classified according to a predefined conceptual framework and categorised and described using qualitative content analysis.

Ethics and dissemination: The review aims at synthesising information from available publications hence it does not require ethical approval. The results will be disseminated through publications, conference presentations and future workshops with stakeholders involved within the recovery paradigm of mental health policy and practice. The scoping review title is registered with the Joanna Briggs Institute.

For peer review only

Strengths and limitations of the study

- This scoping review allows for the systematic synthesis of knowledge on recovery from severe mental illness in to low- and middle-income countries in a rigorous and methodological manner
- This review will highlight the diversity of severe mental illness since there is no agreed upon criteria for including mental illnesses into this category
- Knowledge gaps related to recovery as a multidimensional construct in low- and middle-income countries will be identified
- Only conceptual and theoretical underpinnings of recovery as a construct in LMIC will be discussed without a focus on intervention outcomes.
- Data synthesis will be limited to full text articles available in English only and published between January 1993 and November 2019. Grey literature and unpublished studies in Latin America will be affected by the decision to exclude articles not available in English.

For peer review only

INTRODUCTION

Mental, neurological and substance use (MNS) disorders contribute significantly to the global burden of disease. Whiteford, Ferrari, Degenhardt, Feigin, and Vos[1] report that MNS disorders account for 10% of disability-adjusted life years (DALYs). However, it has been argued that the true global burden of mental health problems may be underestimated by more than a third and that DALYs are closer to **13%.[2]** The lifetime prevalence of severe mental illness (SMIs) ranges between 1% and 4%.[3] Although this is relatively low in comparison to common mental disorders (CMDs), SMIs are more disabling and require complex, long-term interventions for the person and their family.[2] Given the move towards deinstitutionalisation of people with psychiatric disorders, shorter hospital stays are resulting in premature discharge rates and repeated relapse, creating a revolving door phenomenon.[4] These frequent hospital readmissions are costly to the health care system.[5]

In low- and- middle- income countries (LMICs) many people living with a SMI are not detected and as a result do not receive the treatment they need. [3 6 7] In a service utilisation review of World Health Organisation member countries, it was found that the lifetime prevalence of mental disorders amongst adults was between 12% to 49%.[6] The review found the treatment gap to be wide across the psychiatric disorders examined. The treatment gap for schizophrenia and non-affective psychosis was 32%. The gap for other disorders was: major depressive disorder, 56%; bipolar disorder, 50%; and alcohol abuse and dependence, 78%. [6] While present in all countries, this treatment gap is greatest in LMICs where there is a need to scale up mental health services.[8]

A systematic review on the scaling up of mental health services in LMICs found that in many countries the majority of resources for mental health care are rendered on an inpatient or outpatient basis at large psychiatric hospitals, with little provision of services at primary healthcare facilities or via integration between hospitals and the community.[8] Although there are some nascent programmes to provide services for SMI at primary care facilities, these are generally limited because of a poor integration of mental health into primary health care. Additionally, while the lack of coordination between sectors providing mental health services has seen some countries such as India adopt the District Mental Health Programme, the treatment gap continues in rural areas.[9 10]

Despite the drive for deinstitutionalisation and for the rendering of services at decentralised locations, in many countries most of the mental health care budget continues to be allocated to large psychiatric hospitals. In these hospitals, the treatment approach is primarily through medication to alleviate acute clinical symptoms, giving little to no attention to the psychosocial needs of service users.[4 8 11] Unfortunately the sole focus on clinical recovery and the limited

For peer review only - http://bmjopen.bmj.com/site/about/guidelines.xhtml

availability of interventions that focus on rehabilitation and personal recovery further exacerbates the revolving door phenomenon and ongoing treatment gap.[4 12]

Recovery oriented approaches offer an extension to medical models of intervention, as they are not solely focused on symptomatic improvement as a marker of clinical recovery.[13] Personal recovery for people with SMI is conceptually distinct from the medical definition of recovery which is clinical in nature and mostly values symptom remission.[14 15] Personal recovery proposes that a person develops new meaning and purpose in their life over the longitudinal course of their illness.[13 16] Psychiatric rehabilitation has highlighted the need to move beyond treating the symptoms of the illness, to treating its functional and disabling consequences as well.[17] Deegan, a mental health professional and person living with schizophrenia, suggested that mental health service users do not "get rehabilitated" by others, but instead they are active participants in their journey to recover new and valued personal meaning and purpose.[18]

The recovery model was initiated in high income countries (HICs) with the mental health consumer and survivor movement at a time when having a diagnosis of SMI was highly stigmatised and the prognosis was poor.[18] The idea that people with an SMI cannot recover was challenged by people living with schizophrenia who were leading meaningful lives in their communities after deinstutionalisation, highlighting that recovery was not dependant on long term psychiatric treatment.[19 20] For a person living with an SMI, receiving a diagnosis and psychiatric treatment focussed on clinical recovery is a necessary step towards recovery, but personal recovery is also critical given the cyclical, non-linear, long term course of the illness. In Anthony's seminal work,[17] he defined recovery as:

"a deeply personal, unique process of changing ones' attitude, values, feelings, goals, skills and/or roles. It is a way of living a satisfying, hopeful and contributing life even within the limitations caused by illness"

Research exploring the meaning of recovery among people living with SMI,[21-23] found that recovery means different things to different people, supporting a view that there is no definition of recovery that applies to everyone. A literature review conducted in 2018 explored the meaning of recovery amongst people living with schizophrenia.[21] This review included 17 studies explaining the subjective meaning of recovery. A majority of the included studies were qualitative, with a few taking a quantitative approach to exploring the meaning of recovery. Of the 17 papers only four studies were from LMICs (India (n=2), China and South Africa). The results revealed that recovery is complex, non-linear and perceived as both a process and an outcome. The process was viewed as being individual and it was recommended that more consumer perspectives to inform recovery-oriented interventions which are aligned to

For peer review only - http://bmjopen.bmj.com/site/about/guidelines.xhtml

individuals' personal goals and aspirations were needed. The complexity embedded in viewing recovery as a process and an outcome with multidimensional indicators has been documented by others.[24-26] Further to this, it was recommended that clinicians, caregivers and researchers explore more qualitative and personal narratives of recovery, the tools needed to measure recovery and the development of community-based recovery- oriented services.[21]

The aforementioned review was limited to recovery in people living with Schizophrenia and only included four studies from LMIC countries. There is current debate around whether people with SMI in LMICs have a better prognosis and a call to re-examine the realities of living with an SMI amidst social, cultural and economic changes in LMICs.[27 28]

Recovery has been viewed as a process or an outcome, with current debates and research seeking to offer conceptual clarity.[12 29] A systematic review conducted by Leamy et al.[12] led to a proposed conceptual framework for personal recovery in mental illness. The review focused on 97 papers from 13 high income countries and identified characteristics, processes and stages of recovery. While the review included individuals from Black and minority ethnic groups, it did not include any LMICs.[12] The CHIME framework emerged from this review process. This framework conceptualises personal recovery to incorporate the following concepts: **c**onnectedness, **h**ope and optimism about the future, **i**dentity, **m**eaning in life and **e**mpowerment.[12] This highlights that literature on personal recovery is dominated by research conducted in HICs and that there is a gap in understanding recovery from culturally diverse groups.[30]

Research seeking to understand recovery as a process and an outcome is ongoing.[31] The complexity of the concept and the range of factors impacting recovery has been the topic of recent studies. One such focal area has been on the relationship between homelessness amongst people living with severe mental illness and the contribution of stable housing towards recovery.[32 33] Additionally, a recent exploration of metacognition and recovery from mental illness challenged conventional treatment to embrace the notion that recovery is unique, individualised and self-directed and that service users need to be active participants in their recovery processes.[26] A call has been made for mental health service users to be partners in conceptualising recovery as a concept and treatment orientation in order to intergrate the notion of recovery into mainstream psychiatry.[31]

A scoping review of systematic reviews and meta-analyses conducted by van Weeghel et al.[29] mapped the concept of recovery and its assessment. It found that recovery is a personal process that is dynamic and evolves over time.[29] Although 25 studies were included in the review, only one study was conducted by authors from India. Conducting a scoping review focusing specifically on studies conducted in LMICs will contribute to the literature on recovery

For peer review only - http://bmjopen.bmj.com/site/about/guidelines.xhtml

in these specific contexts and will be useful for the following reasons. First, little attention has been given to describing recovery as a personal and individual journey from LMIC perspectives. As recovery for the person with an SMI takes place in a social context through a range of relationships, more understanding about how contexts can help or hinder recovery is needed.[34]

Second, individual meaning-making within recovery is influenced by social, political, economic and human rights factors but further research is needed to understand the diversity of recovery narratives from the perspectives of different population groups.[35] A systematic review and narrative analysis synthesising descriptions and models of personal recovery was undertaken to inform the development of a conceptual framework to guide recovery oriented research and practice.[12] Studies conducted in HIC specifically the United States featured predominantly in the review which included 97 papers. The review culminated in the emergence of a conceptual framework to describe the characteristics of recovery, the processes of recovery and the stages of recovery. The authors reported a difference in studies which included ethnic minorities and where more emphasis was placed on stigma, spirituality, culture and the collectivist nature of recovery. They acknowledged that this area is underexplored.[12]

Third, in many African cultures an individual's personhood is manifested through their agency in relation to spirituality, society and self.[36] This may impact on the person and how they experience mental illness and recovery. Understanding cultural concepts and the dynamics of interconnectedness means recognising that personhood in LMICs is weaved into how people experience health, illness and recovery.[36] Considering the person as a spiritual and social being provides opportunities to collaborate with caregivers and communities in developing appropriate interventions.[36] In countries that are spiritually diverse, multicultural and home to people of different religious backgrounds it is important to contextualise mental illness and explore health professionals own perspectives on culture, psychiatry and mental health and where mental illness must be contextualised.[37 38] Given the reasons cited previously, further exploration into the recovery journeys of people living with SMI in LMICs and how they view the dimensions of recovery is warranted.[21]

A scoping review is proposed as a means of synthesising knowledge on a broadly defined topic and systematically mapping key concepts, theories, evidence and research gaps in the topic area, while still being rigorous and methodological in its approach.[39 40] While systematic reviews and meta-analyses limit their parameters to research trials and quantitative data synthesis, scoping reviews adopt a broader approach to the narrative integration of evidence.[41] We aim to appraise the definitions of recovery and its current utilisation for people with SMI in LMICs. The review findings could be used to inform future practice applications

For peer review only - http://bmjopen.bmj.com/site/about/guidelines.xhtml

which considers the cultural and contextual factors impacting on recovery[42] from SMI outside of HICs. This scoping review title is registered with the Joanna Briggs Institute although JBI and PROSPERO do not provide registry numbers for scoping reviews at this time.[39]

METHODS

A scoping review was deemed as the most suitable method to map existing research on recovery from SMI in LMICs because it allows for the topic to be located, examined, summarised and be presented rapidly and systematically. Additionally it could serve as a precursor to a systematic review.[43] The proposed review will be conducted using the Arksey and O'Malley,[40] scoping review methodology and will be reported on using the PRISMA guidelines[44] with an understanding that there is much development as scoping reviews gain popularity especially in health-related topics.[39 45]

Arksley and O'Malley[40] offer a methodological framework for conducting a scoping review. Their approach describes six stages: (1) identification of the research question, (2) identification of relevant studies, (3) selection of studies, (4) charting the data, (5) collating, summarising and reporting of results and (6) consultation with stakeholders.[40] The last stage is optional according to Arksley and O'Malley,[40] but others have deemed it a necessary step to aide in methodological rigour.[46] Recommendations to enhance the methodology at each stage will be incorporated into this review.[39 44 46]

Stage 1: Identifying the research question

In order to guide the search strategy an iterative process of discussion amongst authors occurred in order to develop and refine the research question. The question needed to be clear enough to inform the subsequent stages while still reflecting the scope of inquiry.[46] Given that scoping studies are focused on summarizing the breadth of evidence on a given topic the authors refined the exploratory question to ask the following:

What is known about recovery from severe mental illness in low-and-middle-income countries?

This broad question was developed to allow for a comprehensive review of the literature in order to review and appraise the definition of recovery and how it is understood in mental health research and practice in LMICs. While many have written about this in high income countries,[23 47] there is a dearth of such information in LMICs .

Stage 2: Identifying relevant studies

For peer review only - http://bmjopen.bmj.com/site/about/guidelines.xhtml

Given the aim of the study is to identify primary published and unpublished studies which will answer the research question, a comprehensive search strategy was developed to aide this. Two health sciences librarians assisted the first author in the iterative process of developing a search strategy including inclusion and exclusion criteria. Decisions were made about time frames, language, search terms and search strategies. Trial searches were run to assess whether relevant literature could be identified using the proposed strategy. A discussion of the identified databases, search strategy and selection criteria follow.

Databases

Electronic databases will be searched to identify studies published between January 1993-November 2019. This period was selected as it covers roughly 25 years in the recovery movement. Personal recovery is a construct that has already been defined by Anthony,[17] hence the authors were looking specifically for literature on this construct. Terms used in other disciplines that could also mean recovery were not included as the focus of the search is on recovery as explicitly defined by the recovery movement. Furthermore, recovery knowledge and attitudes among health professionals will be included if reported in the selected studies.

An expert librarian assisted the first author in identifying information sources that are relevant to the research question. The following databases were selected: MEDLINE via PubMed, SCOPUS (which included contents of Embase), PsycINFO, CINAHL, Africa-Wide Information, PSYCH articles, Health source: Nursing/Academic Edition, Academic Search Premier and SocINDEX all via the EBSCOHOST platform. Additionally, the Latin American and Caribbean Health Sciences Literature (LILACS), the Cochrane Centre Register of Controlled Trials. Grey literature sources will be identified to expand the search and enhance the data sources. Additional sources will be located by means of hand searching reference lists of relevant papers, contacting study authors, searching trial registers, and contacting key personnel involved in recovery-focussed programmes in LMICs. In this way peer reviewed and grey literature from the biomedical sciences, allied health sciences, social sciences and other disciplinary fields will be included.

Search Strategy

Two librarians and the first author developed the eligibility criteria for the scoping review. They identified various definitions of severe mental illness as well as recovery to include in this search strategy. A list of preliminary search terms and filtering methods was developed. The main filtering methods related to the date range of 25 years (January 1993- November 2019). This period was extended so that new publications would not be missed. The search strategy was refined to include MESH terms, filters and Boolean operations to comply with searches across different databases. A search strategy for the PubMed database is included in

For peer review only - http://bmjopen.bmj.com/site/about/guidelines.xhtml

Supplementary file 1. After the initial search, key words and index terms used across the databases were identified and plotted on a table as indicated below (Table 1).

Table 1 Search strategy terms

Keyword	Alternative
Recovery	Recovery OR recover OR psychosocial rehabilitation OR mental health rehabilitation OR psychiatric rehabilitation OR mental health recovery OR Recovery of function OR Quality of life
Severe mental illness	Severe mental illness OR bipolar OR delusional disorder OR delusional disorders OR major depressive disorder OR major depressive disorders OR schizophrenia OR manic OR manic-depressive OR paranoid disorder OR paranoid disorders OR psychoses OR psychosis OR psychotic disorder OR psychotic disorders OR schizoaffective disorder OR schizoaffective disorders OR Schizophreniform OR serious mental disorder OR serious mental disorders Schizophrenia Spectrum and Other Psychotic Disorders Bipolar and Related Disorders Depressive Disorder
Low- and-middle- income countries	Deprived Countries OR Deprived Population OR Deprived Populations OR Developing Countries OR Developing Country OR Developing Economies OR Developing Economy OR Developing Nation OR Developing Nations OR Developing Population OR Developing Populations OR Developing World OR LAMI Countries OR LAMI Country OR Less Developed Countries OR Less Developed Country OR Less Developed Economies OR Less Developed Nation OR Less Developed Nations OR Less Developed World OR Lesser Developed Countries OR Lesser Developed Nations OR LMIC OR LMICS OR Low GDP OR Low GNP OR Low Gross Domestic OR Low Gross National OR Low Income Countries OR Low Income Country OR Low Income Economies OR Low Income Economy OR Low Income Nations OR Low Income Population OR Low Income Populations OR Lower GDP OR lower gross domestic OR Lower Income Countries OR Lower Income Country OR Lower Income Nations OR Lower Income Population OR Lower Income Populations OR Middle Income Countries OR Middle Income Country OR Middle Income Economies OR Middle Income Nation OR Middle Income Nations OR Middle Income Population OR Middle Income Populations OR Poor Countries OR Poor Country OR Poor Economies OR Poor Economy OR Poor Nation OR Poor Nations OR Poor Population OR Poor Populations OR poor world OR Poorer Countries OR Poorer Economies OR Poorer Economy OR Poorer Nations OR Poorer Population OR Poorer Populations OR Third World OR Transitional Countries OR Transitional Country OR Transitional Economies OR Transitional Economy OR Under Developed Countries OR Under Developed Country OR under developed nations OR Under

For peer review only - http://bmjopen.bmj.com/site/about/guidelines.xhtml

	Developed World OR Under Served Population OR Under Served Populations OR Underdeveloped Countries OR Underdeveloped Country OR underdeveloped economies OR underdeveloped nations OR underdeveloped population OR Underdeveloped World OR Underserved Countries OR Underserved Nations OR Underserved Population OR Underserved Populations<br><br>OR<br>Afghanistan OR Albania OR Algeria OR American Samoa OR Angola OR Armenia OR Azerbaijan OR Bangladesh OR Belarus OR Byelarus OR Belorussia OR Belize OR Benin OR Bhutan OR Bolivia OR Bosnia OR Botswana OR Brazil OR Bulgaria OR Burma OR Burkina Faso OR Burundi OR Cabo Verde OR Cape Verde OR Cambodia OR Cameroon OR Central African Republic OR Chad OR China OR Colombia OR Comoros OR Comores OR Comoro OR Congo OR Costa Rica OR Côte d'Ivoire OR Cuba OR Djibouti OR Dominica OR Dominican Republic OR Ecuador OR Egypt OR El Salvador OR Equatorial Guinea OR Eritrea OR Ethiopia OR Fiji OR Gabon OR Gambia OR Gaza OR Georgia OR Georgia Republic OR Ghana OR Grenada OR Grenadines OR Guatemala OR Guinea OR Guinea- Bissau OR Guyana OR Haiti OR Herzegovina OR Hercegovina OR Honduras OR India OR Indonesia OR Iran OR Iraq OR Jamaica OR Jordan OR Kazakhstan OR Kenya OR Kiribati OR Democratic People's Republic of Korea OR Kosovo OR Kyrgyz OR Kirghizia OR Kirghiz OR Kyrgyzstan OR Lao PDR OR Laos OR Lebanon OR Lesotho OR Liberia OR Libya OR Macedonia OR Madagascar OR Malawi OR Malay OR Malaya OR Malaysia OR Maldives OR Mali OR Marshall Islands OR Mauritania OR Mauritius OR Mexico OR Micronesia OR Moldova OR Mongolia OR Montenegro OR Morocco OR Mozambique OR Myanmar OR Namibia OR Nepal OR Nicaragua OR Niger OR Nigeria OR Pakistan OR Palau OR Panama OR Papua New Guinea OR Paraguay OR Peru OR Philippines OR Principe OR Romania OR Ruanda OR Rwanda OR Samoa OR Sao Tome OR Senegal OR Serbia OR Sierra Leone OR Solomon Islands OR Somalia OR South Africa OR South Sudan OR Sri Lanka OR St Lucia OR St Vincent OR Sudan OR Surinam OR Suriname OR Swaziland OR Syria OR Syrian Arab Republic OR Tajikistan OR Tadzhikistan OR Tajikistan OR Tadzhik OR Tanzania OR Thailand OR Timor OR Togo OR Tonga OR Tunisia OR Turkey OR Turkmen OR Turkmenistan OR Tuvalu OR Uganda OR Ukraine OR Uzbek OR Uzbekistan OR Vanuatu OR Venezuela OR Vietnam OR West Bank OR Yemen OR Zambia OR Zimbabwe

For peer review only - http://bmjopen.bmj.com/site/about/guidelines.xhtml

There are no universally accepted guidelines to operationalise the concept of recovery and there is no measure of a gold standard of recovery [48]. The CHIME framework is one proposed guide for the development of recovery measures and identifying recovery outcomes for consideration in clinical practice, but this is limited to HICs. In this review, the key concept is personal recovery as defined by the seminal work of Anthony [17]. In the search strategy, the concept of recovery will be explored through the MeSH terms: Psychiatric Rehabilitation; Mental Health Recovery and Recovery of Function as well as through the free text terms: Recovery OR recover OR psychosocial rehabilitation OR mental health rehabilitation OR psychiatric rehabilitation.

For peer review only

Selection criteria

Articles will be included in the review if they meet the following inclusion and exclusion criteria (Table 2).

Table 2 Inclusion and exclusion criteria

Criteria for inclusion	Criteria for exclusion
• Published between January 1993- November 2019<br>• Qualitative and quantitative empirical (i.e. primary research) study designs<br>• Theoretical literature on the construct of recovery<br>• Studies will be included if they have been conducted in LMICs<br>• Peer reviewed scientific literature<br>• Literature focussing on the population of interest; that is adults with SMI in LMICs will be included. The diagnosis of SMI includes schizophrenia spectrum disorders, schizoaffective disorders, bipolar affective disorders, major depressive disorder or related psychotic disorders (schizophreniform, delusional	• The review will be limited to full text articles written in English. However, the authors acknowledge that this practical decision could mean that potentially relevant papers are missed<br>• Conference abstracts will be excluded although they will be reviewed to see if full reports were published<br>• Children will be excluded as the age range for inclusion is 18 years and older<br>• Duplicate articles from the same study will be excluded<br>• Policy documents will be excluded although they will be reviewed to access research in LMICs that they might have referenced

For peer review only - http://bmjopen.bmj.com/site/about/guidelines.xhtml

disorder, substance induced psychotic disorder or disorders not otherwise specified) as well as those with a comorbid substance use disorder. Studies and reports will be excluded if they do not include the keywords or alternatives as outlined in table 1<br>• Explicit mention of severe mental illness and recovery in LMICS	• Studies that occurred only in high income countries, without a LMIC focus will be excluded<br>• Studies must be conducted in LMIC and not in HIC with participants from LMICs

Stage 3: Study selection

The scoping review guideline for identifying and selecting studies informs article selection and data extraction.[40] Two screening levels are proposed: a title and abstract review and a full-text review. For the first level of screening, two authors (FG, ZS) will independently screen the title and abstracts of all retrieved citations against a few select inclusion criteria. Any articles that seem relevant by either or both reviewers will be included in the full-text review. Next, these two reviewers will independently screen the full text articles to assess whether they meet the inclusion and exclusion criteria. At both levels of screening, inter-rater agreement will be assessed by calculating Cohen's Kappa. Any disagreement on study selection will be resolved by discussion with a third rater, who will be a senior member of the research team.

Quality appraisal

The Mixed Methods Appraisal Tool (MMAT) version 2018[49] and the Critical Appraisal Skills Programme (CASP) Qualitative checklist will be used to assess the quality of included studies although this is not required for a scoping review nor will it influence the inclusion of the study.

Using the aforementioned criteria, the number of studies identified and selected for inclusion in the scoping review will be reported on. A narrative description [39] will accompany the search decision flow chart.[44] The flow chart will show the results from the search, the removal of duplicates, study selection, full retrieval numbers, additional searches from reference lists and a final summary of decisions related to the search decision and an explanation of the review decision process.[39]

For peer review only - http://bmjopen.bmj.com/site/about/guidelines.xhtml

Stage 4: Charting the data

Next, the two reviewers (FG and ZS) will independently extract data from the identified articles and chart it.[40] A draft data charting form will be created in the MS Excel programme to facilitate this process.[40] This form will be pre-tested and an iterative process of refining the form will be undertaken as the review commences to ensure that all relevant information is extracted.[39]

The form will allow the reviewers to confirm study relevance, record study characteristics and extract information relevant to the review question. The following information will be extracted from the articles: 1) study title; 2) author; 3) study population; 4) participant characteristics; 5) research question; 6) study methodology; 7) study description; 8) intervention type (if applicable); 9) intervention duration (if applicable); 10) outcome measures; 11 summary of findings; 12) definitions of key concepts (recovery and severe mental illness); 13) conceptual links identified; 14) practice implications and; 15) recommendations for further development.

While charting the data it is important to maintain flexibility around including emerging categories and consulting with the team as this occurs. The final version of the charting form along with definitions of included items will be provided as an appendix to the review. This process will facilitate the timeous completion of the review while also maintaining research rigour.

Stage 5: Collating, summarising and reporting the results

All results will be collated, summarised and reported on in order to provide a comprehensive and thorough overview of all the reviewed literature. The authors plan to follow an iterative process and not pre-empt or fit findings into what has already been written about recovery in HICs. This will allow new understandings to be uncovered. While the CHIME framework is considered to offer a comprehensive overview of the process of recovery, it has only been used in HICs with predominantly White populations so its applicability with ethnically and culturally diverse groups is unknown.[12 30] The role of non-government organisations (NGOs) and traditional healers in providing services that facilitate recovery for people living with SMIs will be examined if this arises from the scoping review.

A three-step approach[46] of analysing the data, reporting results and applying meaning to results will be followed to provide methodological rigour. Collating and summarising results will include a summary of the types of studies conducted (including the quantity, types of study design, populations, setting) as well as a thematic analysis of the results. More specifically, we will code how studies describe the concept of recovery from SMI and how it is defined and understood in LMICs. The framework used to report the findings will be developed iteratively

For peer review only - http://bmjopen.bmj.com/site/about/guidelines.xhtml

through the examination of results. Additionally, the authors will report on the broader implications of the review in terms of research, policy and practice.

Stage 6: Consultation

While Arksey and O'Malley[40] view the consultation stage as an optional step, Levac[46] views it as a requirement to ensure methodological rigour. During each stage of the review process we will consult with an existing stakeholder advisory committee. This advisory committee consists of the research team and key stakeholders including mental health service providers and service users from within the public health and non-profit organisation (NPO) sectors. This committee has been constituted from the onset of the project and some members are part of a pre-existing group for public mental health specialists. The advisory committee will be consulted to get their views on the relevance of the review, guidance for accessing grey literature and their perspectives on the data extraction process and preliminary findings. Including an advisory committee in the review is informed by a pragmatic approach[50] where the research is treated as a human experience with the understanding that this consultation process is likely to enhance the relevance of this review. It will also support collaborative efforts to facilitate consumer participation, public involvement, access to information and transfer of knowledge.[46]

Patient and public involvement

There is no patient involvement in the protocol development aspect of the study. Service users forming part of the advisory committee referred to in Stage 6 will be consulted on findings and dissemination of results.

Ethics and dissemination

Since the scoping review methodology aims at synthesising information from available publications, this study does not require ethical approval. Reviews of primary research allows for the mapping of evidence in fields where the body of literature has not yet been comprehensively reviewed or where working definitions and concepts are still developing. The findings will be made available in different formats to facilitate its dissemination. An article reporting on the results will be submitted for publication in a peer -reviewed journal. It will also be presented at relevant conferences and stakeholder engagements involved in mental health policy and practice. These stakeholders include MHSUs, academics, policy makers, researcher and clinicians. Findings will also be shared via newsletters, policy briefs and social media forums.

Author Contributions

For peer review only - http://bmjopen.bmj.com/site/about/guidelines.xhtml

This protocol was developed by the intellectual contributions of all the authors. All authors were involved in developing the review question and conceptualising the approach. FG developed and tested the search terms in consultation with a subject librarian. FG and KS developed the data extraction guideline, and this was revised based on feedback from the other authors. RG, KS and BM provided substantial critique and review of the draft protocol.

Funding

This work was supported by the DELTAS Africa Initiative [DEL-15-01]. The DELTAS Africa Initiative is an independent funding scheme of the African Academy of Sciences (AAS)'s Alliance for Accelerating Excellence in Science in Africa (AESA) and supported by the New Partnership for Africa's Development Planning and Coordinating Agency (NEPAD Agency) with funding from the Wellcome Trust [DEL-15-01] and the UK government. The views expressed in this publication are those of the author(s) and not necessarily those of AAS, NEPAD Agency, WellcomeTrust or the UK government. BM is funded through the South African Medical Research Council.

For peer review only - http://bmjopen.bmj.com/site/about/guidelines.xhtml

References

1. Whiteford HA, Ferrari AJ, Degenhardt L, et al. The global burden of mental, neurological and substance use disorders: an analysis from the Global Burden of Disease Study 2010. *PLoS One* 2015;10(2):e0116820.
2. Vigo D, Thornicroft G, Atun R. Estimating the true global burden of mental illness. *Lancet Psychiatry* 2016;3(2):171-78. doi: 10.1016/S2215-0366(15)00505-2
3. Kessler RC, Berglund PA, Bruce ML, et al. The prevalence and correlates of untreated serious mental illness. *Health Serv Res* 2001;36(6 Pt 1):987.
4. Petersen I, Lund C, Stein DJ. Optimizing mental health services in low-income and middle-income countries. *Curr Opin Psychiatry* 2011;24(4):318-23. doi: 10.1097/YCO.0b013e3283477afb
5. Sfetcu R, Musat S, Haaramo P, et al. Overview of post-discharge predictors for psychiatric re-hospitalisations: a systematic review of the literature. *BMC Psychiatry* 2017;17(1) doi: 10.1186/s12888-017-1386-z
6. Kohn R, Saxena S, Levav I, et al. The treatment gap in mental health care. *Bull World Health Organ* 2004;82(11):858-66. doi: 10.1590/S0042-96862004001100011
7. Demyttenaere K, Bruffaerts R, Posada-Villa J, et al. Prevalence, severity, and unmet need for treatment of mental disorders in the World Health Organization World Mental Health Surveys. *JAMA* 2004;291(21):2581-90.
8. Eaton J, McCay L, Semrau M, et al. Scale up of services for mental health in low-income and middle-income countries. *Lancet* 2011;378(9802):1592-603. doi: https://doi.org/10.1016/S0140-6736(11)60891-X
9. Thornicroft G, Cooper S, Bortel TV, et al. Capacity Building in Global Mental Health Research. *Harv Rev Psychiatry* 2012;20(1):13-24. doi: 10.3109/10673229.2012.649117
10. Kumar A. Mental health services in rural India: challenges and prospects. *Health (N Y)* 2011;3(12):757-61.
11. Lund C, Oosthuizen P, Flisher AJ, et al. Pathways to Inpatient Mental Health Care Among People With Schizophrenia Spectrum Disorders in South Africa. *Psychiatr Serv* 2010;61(3):235-40. doi: 10.1176/ps.2010.61.3.235
12. Leamy M, Bird V, Le Boutillier C, et al. Conceptual framework for personal recovery in mental health: systematic review and narrative synthesis. *Br J Psychiatry* 2011;199(6):445-52. doi: 10.1192/bjp.bp.110.083733
13. Slade M. Personal recovery and mental illness: A guide for mental health professionals: Cambridge University Press 2009.
14. Slade M. Mental illness and well-being: the central importance of positive psychology and recovery approaches. *BMC Health Serv Res* 2010;10:26-26. doi: 10.1186/1472-6963-10-26
15. Myers NL, Myers NL. Culture, stress and recovery from schizophrenia: lessons from the field for global mental health. *Cult Med Psychiatry* 2010;34(3):500-28. doi: 10.1007/s11013-010-9186-7
16. Tew J, Ramon S, Slade M, et al. Social factors and recovery from mental health difficulties: a review of the evidence. *Br J Soc Work* 2012;42(3):443-60.
17. Anthony WA. Recovery from mental illness: the guiding vision of the mental health service system in the 1990s. *Psychiatr Rehabil J* 1993;16(4):11.
18. Deegan PE. Recovery: The lived experience of rehabilitation. *Psychiatr Rehabil J* 1988;11(4):11.
19. Webb L. The recovery model and complex health needs: what health psychology can learn from mental health and substance misuse service provision. *J Health Psychol* 2012;17(5):731-41. doi: 10.1177/1359105311425276
20. Farkas M. The vision of recovery today: what it is and what it means for services. *World Psychiatry* 2007;6(2):68.

For peer review only

21. Mathew ST, Nirmala BP, Kommu JVS. Meaning of Recovery Among Persons with Schizophrenia: A Literature Review. *J Psychosoc Rehabil Mental Health* 2018;5(1):73-81. doi: 10.1007/s40737-018-0111-7
22. Kelly M, Lamont S, Brunero S. An Occupational Perspective of the Recovery Journey in Mental Health. *Br J Occup Ther* 2010;73(3):129-35. doi: 10.4276/030802210x12682330090532
23. Cabassa LJ, Nicasio A, Whitley R. Picturing Recovery: A Photovoice Exploration of Recovery Dimensions Among People With Serious Mental Illness. *Psychiatr Serv* 2013;64(9):10.1176/appi.ps.201200503. doi: 10.1176/appi.ps.201200503
24. Aldersey HM, Adeponle AB, Whitley R. Diverse Approaches to Recovery from Severe Mental Illness. The Palgrave Handbook of Sociocultural Perspectives on Global Mental Health2017:109-27.
25. Frost BG, Tirupati S, Johnston S, et al. An Integrated Recovery-oriented Model (IRM) for mental health services: evolution and challenges. *BMC Psychiatry* 2017;17:22. doi: 10.1186/s12888-016-1164-3
26. Lysaker PH, Hamm JA, Hasson-Ohayon I, et al. Promoting recovery from severe mental illness: Implications from research on metacognition and metacognitive reflection and insight therapy. *World J Psychiatry* 2018;8(1):1-11. doi: 10.5498/wjp.v8.i1.1 [published Online First: 2018/03/24]
27. Cohen A, Patel V, Thara R, et al. Questioning an axiom: better prognosis for schizophrenia in the developing world? *Schizophr Bull* 2008;34(2):229-44. doi: 10.1093/schbul/sbm105 [published Online First: 2007/10/02]
28. Patel V, Saxena S, Lund C, et al. The Lancet Commission on global mental health and sustainable development. *Lancet* 2018;392(10157):1553-98.
29. van Weeghel J, van Zelst C, Boertien D, et al. Conceptualizations, assessments, and implications of personal recovery in mental illness: A scoping review of systematic reviews and meta-analyses. *Psychiatr Rehabil J* 2019;42(2):169.
30. Brijnath B. Applying the CHIME recovery framework in two culturally diverse Australian communities: Qualitative results. *Int J Soc Psychiatry* 2015;61(7):660-67. doi: 10.1177/0020764015573084
31. Leonhardt BL, Huling K, Hamm JA, et al. Recovery and serious mental illness: a review of current clinical and research paradigms and future directions. *Expert Rev Neurother* 2017;17(11):1117-30.
32. Kirst M, Zerger S, Harris DW, et al. The promise of recovery: narratives of hope among homeless individuals with mental illness participating in a Housing First randomised controlled trial in Toronto, Canada. *BMJ Open* 2014;4(3):e004379.
33. Smartt C, Prince M, Frissa S, et al. Homelessness and severe mental illness in low-and middle-income countries: scoping review. *BJPsych Open* 2019;5(4)
34. Schön U-K, Denhov A, Topor A. Social relationships as a decisive factor in recovering from severe mental illness. *Int J Soc Psychiatry* 2009;55(4):336-47.
35. Llewellyn-Beardsley J, Rennick-Egglestone S, Callard F, et al. Characteristics of mental health recovery narratives: Systematic review and narrative synthesis. *PLoS One* 2019;14:e0214678. doi: 10.1371/journal.pone.0214678
36. Kpanake L. Cultural concepts of the person and mental health in Africa. *Transcult Psychiatry* 2018;55(2):198-218. doi: 10.1177/1363461517749435
37. Janse van Rensburg ABR. South African Society of Psychiatrists guidelines for the integration of spirituality in the approach to psychiatric practice. *S Afr J Psychiatr* 2014;20(4):133-39. doi: 10.4102/sajpsychiatry.v20i4.593
38. Swartz L, Foster D. Images of culture and mental illness : South African psychiatric approaches. *Social Dynamics* 1984;10(1):17-25. doi: 10.1080/02533958408458354
39. Peters MD, Godfrey CM, Khalil H, et al. Guidance for conducting systematic scoping reviews. *Int J Evid Based Healthc* 2015;13(3):141-46.
40. Arksey H, O'Malley L. Scoping studies: towards a methodological framework. *Int J Soc Res* 2005;8(1):19-32. doi: 10.1080/1364557032000119616
41. Dijkers M. What is a scoping review?. KT Update, 4 (1), 2015.

For peer review only - http://bmjopen.bmj.com/site/about/guidelines.xhtml

42. Myers NL. Culture, stress and recovery from schizophrenia: lessons from the field for global mental health. *Cult Med Psychiatry* 2010;34(3):500-28. doi: 10.1007/s11013-010-9186-7
43. McKinstry C, Brown T, Gustafsson L. Scoping reviews in occupational therapy: the what, why, and how to. *Aust Occup Ther J* 2014;61(2):58-66. doi: 10.1111/1440-1630.12080
44. Tricco AC, Lillie E, Zarin W, et al. Prisma extension for scoping reviews (prisma-scr): Checklist and explanation. *Ann Intern Med* 2018;169(7):467-73. doi: 10.7326/M18-0850
45. Pham MT, Rajić A, Greig JD, et al. A scoping review of scoping reviews: advancing the approach and enhancing the consistency. *Res Synth Methods* 2014;5(4):371-85. doi: doi:10.1002/jrsm.1123
46. Levac D, Colquhoun H, O'Brien KK. Scoping studies: advancing the methodology. *Implement Sci* 2010;5(1):69.
47. Slade M, Longden E. Empirical evidence about recovery and mental health. *BMC Psychiatry* 2015;15:285. doi: 10.1186/s12888-015-0678-4
48. Shanks V, Williams J, Leamy M, et al. Measures of personal recovery: a systematic review. *Psychiatr Serv* 2013;64(10):974-80.
49. Hong QN, Pluye P, Fàbregues S, et al. Mixed methods appraisal tool (MMAT), version 2018. *IC Canadian Intellectual Property Office, Industry Canada* 2018
50. Morgan DL. Pragmatism as a Paradigm for Social Research. *Qual Inq* 2014;20(8):1045-53. doi: 10.1177/1077800413513733

For peer review only

Supplementary file 1

PubMed Search

Population, Concept, Context (PCC)

**Population:**		
#1	MeSH terms:	Schizophrenia Spectrum and Other Psychotic Disorders<br>Bipolar and Related Disorders<br>Depressive disorder, Major
#2	Free text:	Severe mental illness OR bipolar OR delusional disorder OR delusional disorders OR major depressive disorder OR major depressive disorders OR schizophrenia OR manic OR manic-depressive OR paranoid disorder OR paranoid disorders OR psychoses OR psychosis OR psychotic disorder OR psychotic disorders OR schizoaffective disorder OR schizoaffective disorders OR Schizophreniform OR serious mental disorder OR serious mental disorders
#3	#1 OR #2	
**Concept:**		
#4	MeSH terms:	Psychiatric Rehabilitation<br>Mental Health Recovery<br>Recovery of Function
#5	Free text:	Recovery OR recover OR psychosocial rehabilitation OR mental health rehabilitation OR psychiatric rehabilitation
#6	#4 OR #5	
**Context: Low and Middle Income Countries (LMICS)**		
#7	Free text:	Deprived Countries OR Deprived Population OR Deprived Populations OR Developing Countries OR Developing Country OR Developing Economies OR Developing Economy OR Developing Nation OR Developing Nations OR Developing Population OR Developing Populations OR Developing World OR LAMI Countries OR LAMI Country OR Less Developed Countries OR Less Developed Country OR Less Developed Economies OR Less Developed Nation OR Less Developed Nations OR Less Developed World OR Lesser Developed Countries OR Lesser Developed Nations OR LMIC OR LMICS OR Low GDP OR Low GNP OR Low Gross Domestic OR Low Gross National OR Low Income Countries OR Low Income Country OR Low Income Economies OR Low Income Economy OR Low Income Nations OR Low Income Population OR Low Income Populations OR Lower GDP OR lower gross domestic OR Lower Income Countries OR Lower Income Country OR Lower Income Nations OR Lower Income Population OR Lower Income Populations OR Middle Income Countries OR Middle Income Country OR Middle Income Economies OR Middle Income Nation OR Middle Income Nations OR Middle Income Population OR Middle Income Populations OR Poor Countries OR Poor Country OR Poor Economies OR Poor Economy OR Poor Nation OR Poor Nations OR Poor Population OR Poor Populations OR poor world OR Poorer Countries OR Poorer Economies OR Poorer Economy OR Poorer Nations OR Poorer Population OR Poorer Populations OR Third World OR Transitional Countries OR Transitional Country OR Transitional Economies OR Transitional Economy OR Under Developed Countries OR Under Developed Country OR under developed nations OR Under Developed World OR Under Served Population OR Under Served Populations OR Underdeveloped Countries OR Underdeveloped Country OR

For peer review only

		underdeveloped economies OR underdeveloped nations OR underdeveloped population OR Underdeveloped World OR Underserved Countries OR Underserved Nations OR Underserved Population OR Underserved Populations
#8	Free text:	Afghanistan OR Albania OR Algeria OR American Samoa OR Angola OR Armenia OR Azerbaijan OR Bangladesh OR Belarus OR Byelarus OR Belorussia OR Belize OR Benin OR Bhutan OR Bolivia OR Bosnia OR Botswana OR Brazil OR Bulgaria OR Burma OR Burkina Faso OR Burundi OR Cabo Verde OR Cape Verde OR Cambodia OR Cameroon OR Central African Republic OR Chad OR China OR Colombia OR Comoros OR Comores OR Comoro OR Congo OR Costa Rica OR Côte d'Ivoire OR Cuba OR Djibouti OR Dominica OR Dominican Republic OR Ecuador OR Egypt OR El Salvador OR Equatorial Guinea OR Eritrea OR Ethiopia OR Fiji OR Gabon OR Gambia OR Gaza OR Georgia OR Georgia Republic OR Ghana OR Grenada OR Grenadines OR Guatemala OR Guinea OR Guinea- Bissau OR Guyana OR Haiti OR Herzegovina OR Hercegovina OR Honduras OR India OR Indonesia OR Iran OR Iraq OR Ivory Coast OR Jamaica OR Jordan OR Kazakhstan OR Kenya OR Kiribati OR Democratic People's Republic of Korea OR Kosovo OR Kyrgyz OR Kirghizia OR Kirghiz OR Kyrgyzstan OR Lao PDR OR Laos OR Lebanon OR Lesotho OR Liberia OR Libya OR Macedonia OR Madagascar OR Malawi OR Malay OR Malaya OR Malaysia OR Maldives OR Mali OR Marshall Islands OR Mauritania OR Mauritius OR Mexico OR Micronesia OR Moldova OR Mongolia OR Montenegro OR Morocco OR Mozambique OR Myanmar OR Namibia OR Nepal OR Nicaragua OR Niger OR Nigeria OR Pakistan OR Palau OR Papua New Guinea OR Paraguay OR Peru OR Philippines OR Principe OR Romania OR Ruanda OR Rwanda OR Samoa OR Sao Tome OR Senegal OR Serbia OR Sierra Leone OR Solomon Islands OR Somalia OR South Africa OR South Sudan OR Sri Lanka OR St Lucia OR St Vincent OR Sudan OR Surinam OR Suriname OR Swaziland OR Syria OR Syrian Arab Republic OR Tajikistan OR Tadzhikistan OR Tajikistan OR Tadzhik OR Tanzania OR Thailand OR Timor OR Togo OR Tonga OR Tunisia OR Turkey OR Turkmen OR Turkmenistan OR Tuvalu OR Uganda OR Ukraine OR Uzbek OR Uzbekistan OR Vanuatu OR Venezuela OR Vietnam OR West Bank OR Yemen OR Zambia OR Zimbabwe
#9	#7 OR #8	
#10	#3 AND #6 AND #9	887 Results (290519)
Filter	Only language filter used for PubMed as a preliminary search showed that all recent articles not yet indexed were excluded when filtered for NOT animals and year range	

For peer review only

Preferred Reporting Items for Systematic reviews and Meta-Analyses extension for Scoping Reviews (PRISMA-ScR) Checklist

SECTION	ITEM	PRISMA-ScR CHECKLIST ITEM	REPORTED ON PAGE #
**TITLE**			
Title	1	Identify the report as a scoping review.	1
**ABSTRACT**			
Structured summary	2	Provide a structured summary that includes (as applicable): background, objectives, eligibility criteria, sources of evidence, charting methods, results, and conclusions that relate to the review questions and objectives.	2
**INTRODUCTION**			
Rationale	3	Describe the rationale for the review in the context of what is already known. Explain why the review questions/objectives lend themselves to a scoping review approach.	5
Objectives	4	Provide an explicit statement of the questions and objectives being addressed with reference to their key elements (e.g., population or participants, concepts, and context) or other relevant key elements used to conceptualize the review questions and/or objectives.	8
**METHODS**			
Protocol and registration	5	Indicate whether a review protocol exists; state if and where it can be accessed (e.g., a Web address); and if available, provide registration information, including the registration number.	2
Eligibility criteria	6	Specify characteristics of the sources of evidence used as eligibility criteria (e.g., years considered, language, and publication status), and provide a rationale.	11
Information sources*	7	Describe all information sources in the search (e.g., databases with dates of coverage and contact with authors to identify additional sources), as well as the date the most recent search was executed.	9
Search	8	Present the full electronic search strategy for at least 1 database, including any limits used, such that it could be repeated.	9
Selection of sources of evidence†	9	State the process for selecting sources of evidence (i.e., screening and eligibility) included in the scoping review.	11
Data charting process‡	10	Describe the methods of charting data from the included sources of evidence (e.g., calibrated forms or forms that have been tested by the team before their use, and whether data charting was done independently or in duplicate) and any processes for obtaining and confirming data from investigators.	13
Data items	11	List and define all variables for which data were sought and any assumptions and simplifications made.	13
Critical appraisal of individual sources of evidence§	12	If done, provide a rationale for conducting a critical appraisal of included sources of evidence; describe the methods used and how this information was used in any data synthesis (if appropriate).	13

For peer review only - http://bmjopen.bmj.com/site/about/guidelines.xhtml

SECTION	ITEM	PRISMA-ScR CHECKLIST ITEM	REPORTED ON PAGE #
Synthesis of results	13	Describe the methods of handling and summarizing the data that were charted.	14
**RESULTS**			
Selection of sources of evidence	14	Give numbers of sources of evidence screened, assessed for eligibility, and included in the review, with reasons for exclusions at each stage, ideally using a flow diagram.	Click here to enter text.
Characteristics of sources of evidence	15	For each source of evidence, present characteristics for which data were charted and provide the citations.	Click here to enter text.
Critical appraisal within sources of evidence	16	If done, present data on critical appraisal of included sources of evidence (see item 12).	Click here to enter text.
Results of individual sources of evidence	17	For each included source of evidence, present the relevant data that were charted that relate to the review questions and objectives.	Click here to enter text.
Synthesis of results	18	Summarize and/or present the charting results as they relate to the review questions and objectives.	Click here to enter text.
**DISCUSSION**			
Summary of evidence	19	Summarize the main results (including an overview of concepts, themes, and types of evidence available), link to the review questions and objectives, and consider the relevance to key groups.	Click here to enter text.
Limitations	20	Discuss the limitations of the scoping review process.	3
Conclusions	21	Provide a general interpretation of the results with respect to the review questions and objectives, as well as potential implications and/or next steps.	N/A
**FUNDING**			
Funding	22	Describe sources of funding for the included sources of evidence, as well as sources of funding for the scoping review. Describe the role of the funders of the scoping review.	15

JBI = Joanna Briggs Institute; PRISMA-ScR = Preferred Reporting Items for Systematic reviews and Meta-Analyses extension for Scoping Reviews.

* Where *sources of evidence* (see second footnote) are compiled from, such as bibliographic databases, social media platforms, and Web sites.

† A more inclusive/heterogeneous term used to account for the different types of evidence or data sources (e.g., quantitative and/or qualitative research, expert opinion, and policy documents) that may be eligible in a scoping review as opposed to only studies. This is not to be confused with *information sources* (see first footnote).

‡ The frameworks by Arksey and O'Malley (6) and Levac and colleagues (7) and the JBI guidance (4, 5) refer to the process of data extraction in a scoping review as data charting.

§ The process of systematically examining research evidence to assess its validity, results, and relevance before using it to inform a decision. This term is used for items 12 and 19 instead of "risk of bias" (which is more applicable to systematic reviews of interventions) to include and acknowledge the various sources of evidence that may be used in a scoping review (e.g., quantitative and/or qualitative research, expert opinion, and policy document).

From: Tricco AC, Lillie E, Zarin W, O'Brien KK, Colquhoun H, Levac D, et al. PRISMA Extension for Scoping Reviews (PRISMA-ScR): Checklist and Explanation. Ann Intern Med. ;169:467–473. doi: 10.7326/M18-0850

For peer review only
