# Supplementary material for: Exploration of recovery of people living with severe mental illness (SMI) in low-income and middle-income countries (LMIC): a scoping review protocol
Source: BMJ Open. 2020 Feb 3;10(2):e032912. doi: 10.1136/bmjopen-2019-032912 (PMC7044907; doi:10.1136/bmjopen-2019-032912)
Supplement: Reviewer comments [file bmjopen-2019-032912.reviewer_comments.pdf]

PEER REVIEW HISTORY

BMJ Open publishes all reviews undertaken for accepted manuscripts. Reviewers are asked to complete a checklist review form (**http://bmjopen.bmj.com/site/about/resources/checklist.pdf**) and are provided with free text boxes to elaborate on their assessment. These free text comments are reproduced below.

ARTICLE DETAILS

**TITLE (PROVISIONAL)**	An exploration of recovery of people living with severe mental illness (SMI) in low- and- middle -income countries (LMIC): a scoping review protocol
**AUTHORS**	Gamieldien, Fadia; Galvaan, Roshan; Myers, Bronwyn; Sorsdahl, Katherine

VERSION 1 – REVIEW

**REVIEWER**	Martin Agrest<br>Proyecto Suma, Argentina
**REVIEW RETURNED**	13-Aug-2019

**GENERAL COMMENTS**	This is a solid protocol, with an adequate rationale and a very good methods section, addressing an important knowledge gap in the literature. Recovery has been widely studied in high- income countries (HICs) in the last twenty years, but initiatives in low- and middle- income countries (LMICs) are strongly underrepresented in the literature. The undergoing mental health services reform in LMICs (i.e., the deinstitutionalisation process) may benefit from a research on how recovery is experimented and conceptualized in these countries.<br><br>The Abstract is very good, clear and thorough.<br><br>Strengths and limitations are good. See further comments regarding the limitations below.<br><br>Introduction is good. The authors frame their work in terms of the importance of mental illness and disability-adjusted life years (DALYs) and the global movement towards deinstitutionalization of people with severe mental illness (SMI). The authors then mention that in low- and middle- income countries (LMICs), the treatment gap is wider than in high- income countries (HICs). However, it may be interesting to mention here that there is profound discussion about people with SMI having better prognosis in LMICs compared to HICs (Jablensky A, Sartorius N, Ernberg G, et al. Schizophrenia: manifestations, incidence and course in different cultures. A World Health Organization ten-country study. Psychol Med Monogr Suppl. 1992;20:1–97; Cohen A, Patel V, Thara R, & Gureje O. Questioning an Axiom: Better Prognosis for Schizophrenia in the Developing World? Schizophrenia Bulletin 2008, vol. 34 no. 2 pp. 229–244). This may also contribute to the authors’ main idea about the importance of understanding how recovery is conceptualized in LMICs, which is likely to be different from HICs.<br>On the other side, the authors seem to give mental health services a greater importance than traditional recovery supporters give to

	mental health services when trying to understand recovery. This is not necessarily a problem, but it may be important to mention that globally the consumer and survivor movement typically places less emphasis on services than authors are doing in their introduction of this protocol.<br><br>Methods section is excellent. One minor detail is that, at least in Latin America, interesting and important grey literature and unpublished studies most probably will not be in English but in Spanish and Portuguese. Even if "the authors acknowledge that this practical decision could mean that potentially relevant papers are missed", the authors may want to consider incorporating literature in Spanish and Portuguese or being more specific as to how the authors can anticipate that this will be a limitation (e.g., saying that, in particular, access to grey literature and unpublished studies in Latin America will be importantly affected by this decision). A greater emphasis on this limitation may be important to be acknowledged by the authors.<br><br>Ethics and dissemination section is fine.<br><br>Conclusion, Author contributions, Funding and References are fine.

**REVIEWER**	Marta Imamura<br>Departamento de Medicina Legal, Etica Medica e Medicina Social e do Trabalho<br>Faculdade de Medicina, Universidade de Sao Paulo, Sao Paulo, SP, BRAZIL. marta.imamura@fm.usp.br
**REVIEW RETURNED**	31-Aug-2019

**GENERAL COMMENTS**	An exploration of recovery of people living with severe mental illness (SMI) in low- and- middle -income countries (LMIC): a scoping review protocol<br><br>Authors present a scoping review protocol that aims to systematically search what is known from existing literature about recovery from severe illness in low- and middle-income countries. Authors provide the rationale and the background to justify a scoping review covering this large, multidimensional, complex and heterogeneous topic.<br><br>The topic is of clinical relevance as it may inform future recovery-oriented approaches and services. The protocol is clearly presented and follows a sound methodological process.<br><br>However, some issues should be better clarified before the scoping review protocol is approved for publication:<br><br>1. Authors should provide a clearer conceptual framework of the current knowledge obtained from the studies conducted in high income countries, that may guide all the aspects involved in the complex and heterogeneous process of the recovery from severe mental illness.<br>2. Languages of the included literature should be explicit mentioned: English, French, Arabic, Spanish, Portuguese, African languages, all languages?<br>3. Authors provide a comprehensive, detailed and complete search strategy mainly for the population to be covered: people with mental illness from low- and middle-income countries. On the

	other hand, the search terms included for the recovery outcomes should also be provided in detail, as some of the recovery outcomes may not be captured using a more general search strategy. Authors should consider adding some relevant terms: social participation; employment; employment, supported; power; volunteers, mental health recovery terms (learning, creative practice, mutuality); interpersonal relations)<br>4. Will authors also include recovery knowledge and attitudes among health professionals? Data from recovery knowledge inventory and other validated tools will also be included?<br>5. Scoping reviews do not require risk of bias assessment or quality appraisal. Will authors conduct any quality assessment of the included studies?<br>6. Is the protocol already registered? Please, provide the registry number/information.<br>7. Reference list should be updated.

**REVIEWER**	Roy Abraham Kallivayalil<br>Professor & Head, Department of Psychiatry<br>Pushpagiri Institute of Medical Sciences,<br>Thiruvalla, Kerala- 689 101, India
**REVIEW RETURNED**	08-Sep-2019

**GENERAL COMMENTS**	"LMICs ----- with little provision of services at primary healthcare facilities or via integration between hospitals and the community"- A mention should be made here about welcome initiatives like District Mental Programme in countries like India.<br>Besides, the role of NGOs (Non Governmental Organisations) in facilitating recovery in LMICs should be examined. Role of traditional healers in many LMICs also needs study.

VERSION 1 – AUTHOR RESPONSE

**Reviewers comments**	Response to comments	Amendments made
Reviewer 1		
Methods section is excellent. One minor detail is that, at least in Latin America, interesting and important grey literature and unpublished studies most probably will not be in English but in Spanish and Portuguese. Even if “the authors acknowledge that this practical decision could mean that potentially relevant papers are missed”, the authors may want to consider incorporating literature in Spanish and Portuguese or being more specific as to how the authors can anticipate that this will be a limitation (e.g., saying that, in particular, access to grey literature and unpublished studies in Latin America will be importantly affected by this decision). A greater emphasis on this limitation may be important to be acknowledged by the authors.	This is noted and acknowledged by the authors	Added in limitations section (page 3)<br><br>Data synthesis will be limited to full text articles available in English only published between 1993 and 2019. Grey literature and unpublished studies in Latin America will be affected by the decision to exclude articles not available in English.

Reviewer 2		
Comment 1<br>Authors should provide a clearer conceptual framework of the current knowledge obtained from the studies conducted in high income countries, that may guide<br>all the aspects involved in the complex and heterogeneous process of the recovery from severe mental illness.	The authors plan to follow an iterative process and not preempt or fit findings into what has already been written about recovery in HICs. This will allow new understadnings to be uncovered.	Added: page 11 (Stage 5) The authors plan to follow an iterative process and not pre-empt or fit findings into what has already been written about recovery in HICs. This will allow new understandings to be uncovered
2. Languages of the included literature should be explicit mentioned: English, French, Arabic, Spanish, Portuguese, African languages, all languages?	Indicated that only English would be used and also only articles available in full text English	See page 2 and page 11
3. Authors provide a comprehensive, detailed and complete search strategy mainly for the population to be covered: people with mental illness from low- and middle-income countries. On the other hand, the search terms included for the recovery outcomes should also be provided in detail, as some of the recovery outcomes may not be captured using a more general search strategy. Authors should consider adding some relevant terms: social participation; employment; employment, supported; power; volunteers, mental health recovery terms (learning, creative practice, mutuality); interpersonal relations)	Personal recovery is a construct that has already been defined, hence the authors were looking specifically for literature on this construct. Terms used in other disciplines that could also mean recovery were not included as the explicit mention of recovery as it is understood in recovery movement was the focus of the search	
4. Will authors also include recovery knowledge and attitudes among health professionals? Data from recovery knowledge inventory and other validated tools will also be included?	If this comes up in the database search then it will be included but same rationale applies to comments 3 and 4.	

5. Scoping reviews do not require risk of bias assessment or quality appraisal. Will authors conduct any quality assessment of the included studies?	Quality assessment will be conducted using the mixed methods assessment tool	Page 12<br>**Quality appraisal**<br>The Mixed Methods Appraisal Tool (MMAT) version 2018[1] and the Critical Appraisal Skills Programme (CASP) Qualitative checklist will used to assess the quality of included studies although this is not required for a scoping review nor will it influence the inclusion of the study.
6. Is the protocol already registered? Please, provide the registry number/information.	Yes, with Joanna Briggs Institute. They do not provide a registry number for scoping reviews but indicate the wording to be used for registered protocols.	Page 2<br>The scoping review title is registered with the Joanna Briggs Institute.
7. Reference list should be updated.	Attended to	
**Reviewer 3**		
"LMICs ----- with little provision of services at primary healthcare facilities or via integration between hospitals and the community"- A mention should be made here about welcome initiatives like District Mental Programme in countries like India. Besides, the role of NGOs (Non Governmental Organisations) in facilitating recovery in LMICs should be examined. Role of traditional healers in many LMICs also needs study.	The role of traditional healers will be discussed based on what emerges from the review	

VERSION 2 – REVIEW

**REVIEWER**	Martin Agrest<br>Proyecto Suma, Argentina
**REVIEW RETURNED**	28-Oct-2019

**GENERAL COMMENTS**	Requested changes in the first revision by this reviewer were done by authors

REVIEWER	Marta Imamura<br>Instituto de Medicina Fisica e Reabilitacao. Hospital das Clinicas HCFMUSP, Faculdade de Medicina, Universidade de Sao Paulo, Sao Paulo, SP, BR.marta.imamura@fm.usp.br<br><br>BRAZIL
REVIEW RETURNED	02-Nov-2019

GENERAL COMMENTS	An exploration of recovery of people living with severe mental illness (SMI) in low- and- middle -income countries (LMIC): a scoping review protocol<br><br>Authors present the R1 version of a scoping review protocol that aims to systematically search what is known from existing literature about recovery from severe illness in low- and middle-income countries.<br><br>In the R1 version authors have properly addressed some of the points raised in the peer review process:<br><br>1. Authors clarify that English will be the only language of the scoping review and list this as a limitation of the study<br>2. Authors explain that the Mixed Methods Appraisal Tool version 2018 and the Critical Appraisal Skills Programme Qualitative checklist will be used to assess the quality of the included studies.<br><br>However, some pointed issues have not been clarified or modified in the R1 version:<br><br>3. Authors have not provided a clearer conceptual framework of the current knowledge obtained from the studies conducted in high income countries, that may guide all the aspects involved in the complex and heterogeneous process of the recovery from severe mental illness.<br>4. Authors provide tentative search strategies in a preliminary search ran during the periods of 10-20 May 2019. However, a comprehensive, detailed and complete search strategy for the recovery outcomes should also be provided in the methods session.<br>5. Authors should also clarify whether they will include recovery knowledge and attitudes among health professionals in the methods session.<br>6. Authors state that the protocol is registered with the Joanna Briggs Institute (R1 version, Abstract session, page 26, lines 47-48), however registry number/information is not provided.<br>7. Reference list has not been updated. No clarifications have been provided on this regard.<br>8. If the literature search has been already performed “during the period of May-June 2019” (R1 version, Abstract session, page 26, line 26) authors should present the scoping review rather than its protocol.

VERSION 2 – AUTHOR RESPONSE

**Reviewers comments**	Response to comments	Amendments made
**Reviewer 2**		
3. Authors have not provided a clearer conceptual framework of the current knowledge obtained from the studies conducted in high income countries, that may guide all the aspects involved in the complex and heterogeneous process of the recovery from severe mental illness.	Personal recovery is a construct that has already been defined by Anthony [1] hence the authors were looking specifically for literature on this construct. Terms used in other disciplines that could also mean recovery were not included as the explicit mention of recovery as it is understood in the recovery movement was the focus of the search. In the literature there is much debate on whether recovery is a process or an outcome and research is underway to provide conceptual clarity on this [2].	Page 6<br><br>Recovery has been viewed as a process or an outcome, with current debates and research seeking to offer conceptual clarity [2]. A systematic review conducted by Leamy et al.[2] led to a proposed conceptual framework for personal recovery in mental illness. The review focused on 97 papers from 13 high income countries and identified characteristics, processes and stages of recovery. While the review included individuals from Black and minority ethnic groups, it did not include any LMICs [2]. The CHIME framework emerged from this review process. This framework conceptualises personal recovery to incorporate the following concepts: **c**onnectedness, **h**ope and optimism about the future, **i**dentity, **m**eaning in life and **e**mpowerment [2]. This highlights that literature on personal recovery is dominated by research conducted in HICs and that there is a gap in understanding recovery from culturally diverse groups[3].<br><br>Page 8<br><br>Personal recovery is a construct that has already been defined by Anthony [1] hence the authors were looking specifically for literature on this construct. Terms used in other disciplines that could also mean recovery were not included as the focus of the search is on recovery as explicitly defined by the recovery movement.
4. Authors provide tentative search	Recovery outcomes have not been specified in the search strategy because of the debate around what constitutes recovery and the	Page 11<br><br>There are no universally accepted guidelines to operationalise the concept

strategies in a preliminary search ran during the periods of 10-20 May 2019. However, a comprehensive, detailed and complete search strategy for the recovery outcomes should also be provided in the methods session.	dominance of literature on recovery emanating from HICs. A scoping review is an iterative process of mapping what is known about a concept. In this review the key concept is personal recovery as defined by the seminal work of Anthony [1]<br><br>In the search strategy the concept of recovery is explored through the MeSH terms: Psychiatric Rehabilitation<br><br>Mental Health Recovery<br><br>and Recovery of Function and<br><br>Free text terms: Recovery OR recover OR psychosocial rehabilitation OR mental health rehabilitation OR psychiatric rehabilitation<br><br>The idea behind the scoping review is to find out how recovery is conceptualised in LMICs. Personal recovery is viewed as a process and outcome in HICs and while research in this area is ongoing and there are proposed outcomes this might look different in LMICs. The authors do not want to pre-empt recovery outcomes but rather want to engage with the data in an iterative manner and the outcomes that emerge will be discussed in the review discussion	of recovery and there is no measure of a gold standard of recovery[4]. The CHIME framework is one proposed guide for the development of recovery measures and identifying recovery outcomes for consideration in clinical practice, but this is limited to HICs. In this review, the key concept is personal recovery as defined by the seminal work of Anthony [1]. In the search strategy, the concept of recovery will be explored through the MeSH terms: Psychiatric Rehabilitation; Mental Health Recovery and Recovery of Function as well as through the free text terms: Recovery OR recover OR psychosocial rehabilitation OR mental health rehabilitation OR psychiatric rehabilitation.
5. Authors should also clarify whether they will include recovery knowledge and attitudes among health professionals in the methods session.		Page 8<br><br>Furthermore, recovery knowledge and attitudes among health professionals will be included if reported in the selected studies

6. Authors state that the protocol is registered with the Joanna Briggs Institute (R1 version, Abstract session, page 26, lines 47-48), however registry number/information is not provided.	JBI and PROSPERO do not provide a registry number for scoping reviews at this stage but have provided the wording to be used for the registered protocol. The reviewers attention is directed to the 2017 Guidance for the conduct of JBI Scoping reviews (p.15) [5]. Article included in supplementary material along with email correspondence regarding the registration of the protocol with JBI. Registration with JBI does not come with an ID number but it is available on the link below (as per email)<br><br>https://joannabriggs.org/research/registered_titles.aspx	Page 2<br><br>The scoping review title is registered with the Joanna Briggs Institute. https://joannabriggs.org/research/registered_titles.aspx<br><br>Page 7<br><br>This scoping review title is registered with the Joanna Briggs Institute (https://joannabriggs.org/research/registered_titles.aspx) although JBI and PROSPERO do not provide registry numbers for scoping reviews at this time[6].
7. Reference list has not been updated. No clarifications have been provided on this regard.	The referencing style for BMJ has been used. Vancouver with abbreviated journals	
8. If the literature search has been already performed "during the period of May-June 2019" (R1 version, Abstract session, page 26, line 26) authors should present the scoping review rather than its protocol.	The literature search period has been updated. Sentence added.	Page 9<br><br>The main filtering methods related to the date range of 25 years (January 1993-November 2019). This period was extended so that new publications would not be missed.<br><br>Page 2<br><br>… will be searched in the May-December 2019.<br><br>Studies published between January 1993 and November 2019...<br><br>Page3<br><br>Data synthesis will be limited to full text articles available in English only

		published between January 1993 and November 2019
Authors have not provided a clearer conceptual framework of the current knowledge obtained from the studies conducted in high income countries, that may guide all the aspects involved in the complex and heterogeneous process of the recovery from severe mental illness.	The authors plan to follow an iterative process and not pre-empt or fit findings into what has already been written about recovery in HICs. The authors have also included the CHIME framework as one mechanism to describe the recovery process although this has only been used in HICs with predominantly White populations so its applicability with ethnically and culturally diverse groups is unknown.	Added: page 14 (Stage 5)<br><br>While the CHIME framework is considered to offer a comprehensive overview of the process of recovery, it has only been used in HICs with predominantly White populations so its applicability with ethnically and culturally diverse groups is unknown[2 3].
**Reviewer 3**		
"LMICs ----- with little provision of services at primary healthcare facilities or via integration between hospitals and the community"- A mention should be made here about welcome initiatives like District Mental	The roles of service providers, including NGOs and traditional healers, will be reported on based on what emerges during the process of charting the data as this is an iterative process which allows for the charting table to be updated as useful data becomes evident[6]	Page 4<br><br>Although there are some nascent programmes to provide services for SMI at PHC facilities, these are generally limited because of a poor integration of mental health into primary care. Additionally, while the lack of coordination between sectors providing mental health services has seen some countries such as India adopt the District Mental Health Programme, the treatment gap continues in rural areas[7 8].<br><br>Page 14<br><br>The role of non-government organisations (NGOs) and traditional healers in providing services that facilitate recovery for people living with

Programme in countries like India.<br><br>Besides, the role of NGOs (Non-Governmental Organisations) in facilitating recovery in LMICs should be examined. Role of traditional healers in many LMICs also needs study.		SMIs will be examined if they arise from the scoping review.
Please revise the sentence "Studies published between 1993 and 2019...." to include the full systematic search dates in the Abstract (month and year).	Amended	Page 2: Abstract<br><br>Inserted -<br><br>…will be searched between May and December 2019.<br><br>"Studies published between January 1993 and November 2019…"<br><br>Page 3<br><br>Data synthesis will be limited to full text articles available in English only and published between January 1993 and November 2019.<br><br>Page 9<br><br>This period was extended so that new publications would not be missed.<br><br>Page 12<br><br>Published between January 1993-November 2019
Please remove your Conclusions	Removed	

sections as this section is not a requirement for study protocols.		

References (these are included in the amended article and the numbering follows the in text citations in the scoping review protocol)

1. Anthony WA. Recovery from mental illness: the guiding vision of the mental health service system in the 1990s. *Psychosocial rehabilitation journal* 1993;16(4):11.
2. Leamy M, Bird V, Le Boutillier C, et al. Conceptual framework for personal recovery in mental health: systematic review and narrative synthesis. *Br J Psychiatry* 2011;199(6):445-52. doi: 10.1192/bjp.bp.110.083733
3. Brijnath B. Applying the CHIME recovery framework in two culturally diverse Australian communities: Qualitative results. *Int J Soc Psychiatry* 2015;61(7):660-67. doi: 10.1177/0020764015573084
4. Shanks V, Williams J, Leamy M, et al. Measures of personal recovery: a systematic review. *Psychiatr Serv* 2013;64(10):974-80.
5. Aromataris E, Munn Z. Joanna Briggs Institute reviewer's manual. *The Joanna Briggs* 2017;299
6. Peters MD, Godfrey CM, Khalil H, et al. Guidance for conducting systematic scoping reviews. *International journal of evidence-based healthcare* 2015;13(3):141-46.
7. Kumar A. Mental health services in rural India: challenges and prospects. *Health (N Y)* 2011;3(12):757-61.
8. Thornicroft G, Cooper S, Bortel TV, et al. Capacity Building in Global Mental Health Research. *Harv Rev Psychiatry* 2012;20(1):13-24. doi: 10.3109/10673229.2012.649117

VERSION 3 – REVIEW

**REVIEWER**	Marta Imamura<br>Instituto de Medicina Fisica e Reabilitacao, Hospital das Clinicas HCFMUSP, Faculdade de Medicina, Universidade de Sao Paulo, BRAZIL
**REVIEW RETURNED**	09-Dec-2019

**GENERAL COMMENTS**	An exploration of recovery of people living with severe mental illness (SMI) in low- and- middle -income countries (LMIC): a scoping review protocol<br><br>Authors present the R2 version of a scoping review protocol that aims to systematically search what is known from existing literature about recovery from severe illness in low- and middle-income countries. All the answers were provided and highlighted in red in the R2 version of the scoping review protocol.<br><br>In the R2 version authors have properly addressed all the points raised in the peer review process:<br><br>1. Authors provided information about the conceptual framework of the current knowledge obtained from the studies conducted in high income countries based on a systematic review by Leamy et al., 2011 (R2 version, Introduction session, page 30, lines 19 to 35).

	2. Authors clarified the search strategy for the recovery outcomes (R2 version, Methods session, page 35, lines 42-55) and also extended the search period to December 2019 (R2 version, Abstract session, page 26, line 26). Authors have also clarified that studies published between January 1993 and November 2019 will be included in the scoping review (R2 version, Abstract session, page 26, line30; page 27, lines 15-16; R2 version, Methods session, page 32, lines 54-55; page 33, lines 32-34; page 36, lines 14-16).<br>3. Authors have clearly stated that the key concept of the scoping review is personal recovery (page 36, lines 47-48).<br>4. Authors explained that the Joanna Briggs Institute does not provide a registry number for scoping reviews (R2 version, Methods session, page 31, lines 40-43). However, as requested, in the R2 versios authors provide information about the registry: link of the scoping review registry at the Joanna Briggs Institute (https://joannabriggs.org/research/registered_titles.aspx) dated 2019-07-03 (R2 version, Abstract session, page 26, line 50; Introduction session page 31, lines 40-41).<br>5. Reference list has not been updated. Authors focus the rationale for the scoping review in the findings of the systematic review published by Leamy et al., 2011. However, updated literature has provided some novel insights with regards to personal recovery from mental illness: please, find some examples below:<br>a. Leonhardt BL, Huling K, Hamm JA, Roe D, Hasson-Ohayon I, McLeod HJ, Lysaker PH. Recovery and serious mental illness: a review of current clinical and research paradigms and future directions. Expert Rev Neurother. 2017 Nov;17(11):1117-1130.<br>b. Lysaker PH, Hamm JA, Hasson-Ohayon I, Pattison ML, Leonhardt BL. Promoting recovery from severe mental illness: Implications from research on metacognition and metacognitive reflection and insight therapy. World J Psychiatry. 2018 Mar 22;8(1):1-11.<br>c. van Weeghel J, van Zelst C, Boertien D, Hasson-Ohayon I. Conceptualizations, assessments, and implications of personal recovery in mental illness: A scoping review of systematic reviews and meta-analyses. Psychiatr Rehabil J. 2019 Jun;42(2):169-181.<br>d. Kirst M, Zerger S, Wise Harris D, Plenert E, Stergiopoulos V. The promise of recovery: narratives of hope among homeless individuals with mental illness participating in a Housing First randomised controlled trial in Toronto, Canada. BMJ Open. 2014 Mar 3;4(3):e004379<br>e. Smartt C, Prince M, Frissa S, Eaton J, Fekadu A, Hanlon C. Homelessness and severe mental illness in low- and middle-income countries: scoping review. BJPsych Open. 2019 Jun 24;5(4):e57. doi: 10.1192/bjo.2019.32.<br><br>6. Authors extended the search period to December 2019 (R2 version, Abstract session, page 26, line 26).

VERSION 3 – AUTHOR RESPONSE

**Reviewers comments**<br><br>**Reviewer 2**	Response to comments	Amendments made

5. Reference list has not been updated. No clarifications have been provided on this regard.	The reference list and in-text citations have been changed as per the BMJ Open Journal recommendations. Period before superscript for in-text citations and abbreviated journals included in reference list. Please direct the authors' attention to any other reference errors or omissions that are related to this comment	
Authors focus the rationale for the scoping review in the findings of the systematic review published by Leamy et al., 2011. However, updated literature has provided some novel insights with regards to personal recovery from mental illness: please, find some examples below: a. Leonhardt BL, Huling K, Hamm JA, Roe D, Hasson-Ohayon I, McLeod HJ, Lysaker PH. Recovery and serious mental illness: a review of current clinical and research paradigms and future directions. Expert Rev Neurother. 2017 Nov;17(11):1117-1130. b. Lysaker PH, Hamm JA, Hasson-Ohayon I, Pattison ML, Leonhardt BL. Promoting recovery from severe mental illness: Implications from research on metacognition and metacognitive reflection and insight therapy. World J Psychiatry. 2018 Mar 22;8(1):1-11.	Thank you for these updated references. These have been relevant and the authors acknowledge their importance. The have been incorporated into a short summary on page 6 however the authors feel they be cited in the review and given this is the protocol theu have not been expanded on.	Added Page 6<br><br>Research seeking to understand recovery as a process and an outcome is ongoing.[2] The complexity of the concept and the range of factors impacting recovery has been the topic of recent studies. One such focal area has been on the relationship between homelessness amongst people living with severe mental illness and the contribution of stable housing towards recovery.[3] [4] Additionally, a recent exploration of metacognition and recovery from mental illness challenged conventional treatment to embrace the notion that recovery is unique, individualised and self-directed and that service users need to be active

c. van Weeghel J, van Zelst C, Boertien D, Hasson-Ohayon I. Conceptualizations, assessments, and implications of personal recovery in mental illness: A scoping review of systematic reviews and meta-analyses. Psychiatr Rehabil J. 2019 Jun;42(2):169-181.<br>d. Kirst M, Zerger S, Wise Harris D, Plenert E, Stergiopoulos V. The promise of recovery: narratives of hope among homeless individuals with mental illness participating in a Housing First randomised controlled trial in Toronto, Canada. BMJ Open. 2014 Mar 3;4(3):e004379<br>e. Smartt C, Prince M, Frissa S, Eaton J, Fekadu A, Hanlon C. Homelessness and severe mental illness in low- and middle-income countries: scoping review. BJPsych Open. 2019 Jun 24;5(4):e57. doi: 10.1192/bjo.2019.32.		participants in their recovery processes.[5] A call has been made for mental health service users to be partners in conceptualising recovery as a concept and treatment orientation in order to intergrate the notion of recovery into mainstream psychiatry.[2]<br><br>A scoping review of systematic reviews and meta-analyses conducted by van Weeghel et al.[6] mapped the concept of recovery and its assessment. It found that recovery is a personal process that is dynamic and evolves over time.[6] Although 25 studies were included in the review, only one study was conducted by authors from India.
**Reviewer 3**	Addressed previously	
Besides, the role of NGOs (Non-Governmental Organisations) in facilitating recovery in LMICs should be examined. Role of	The roles of service providers, including NGOs and traditional healers, will be reported on based on what emerges during the process of charting the data as this is an iterative process which allows for the charting table to be updated as useful data becomes evident[1]	Page 14<br><br>The role of non-government organisations (NGOs) and traditional

traditional healers in many LMICs also needs study.		healers in providing services that facilitate recovery for people living with SMIs will be examined if they arise from the scoping review.
We note that the link provided in the Abstract is broken, please provide an alternate link or remove this from your manuscript.	There appears to be a permanent problem with the link and I have emailed the JBI administrators about this.<br><br>Removed link https://joannabriggs.org/research/registered_titles.aspx<br><br>The authors think it would be best to remove the link from the article and interested parties can go directly to the website. As per the supplementary material submitted for your attention, the protocol has been registered so a search of the scoping review title should register relevant information regarding title registration	Page 2<br><br>The scoping review title is registered with the Joanna Briggs Institute.<br><br>Removed link<br><br>Page 8<br><br>This scoping review title is registered with the Joanna Briggs Institute although JBI and PROSPERO do not provide registry numbers for scoping reviews at this time[1]. Removed link

References (these are included in the amended article and the correct numbering follows the in text citations in the scoping review protocol)

1. Peters MD, Godfrey CM, Khalil H, et al. Guidance for conducting systematic scoping reviews. *Int J Evid Based Healthc* 2015;13(3):141-46.
2. Leonhardt BL, Huling K, Hamm JA, et al. Recovery and serious mental illness: a review of current clinical and research paradigms and future directions. *Expert Rev Neurother* 2017;17(11):1117-30.
3. Kirst M, Zerger S, Harris DW, et al. The promise of recovery: narratives of hope among homeless individuals with mental illness participating in a Housing First randomised controlled trial in Toronto, Canada. *BMJ Open* 2014;4(3):e004379.
4. Smartt C, Prince M, Frissa S, et al. Homelessness and severe mental illness in low-and middle-income countries: scoping review. *BJPsych Open* 2019;5(4)

5. Lysaker PH, Hamm JA, Hasson-Ohayon I, et al. Promoting recovery from severe mental illness: Implications from research on metacognition and metacognitive reflection and insight therapy. *World J Psychiatry* 2018;8(1):1-11. doi: 10.5498/wjp.v8.i1.1 [published Online First: 2018/03/24]
6. van Weeghel J, van Zelst C, Boertien D, et al. Conceptualizations, assessments, and implications of personal recovery in mental illness: A scoping review of systematic reviews and meta-analyses. *Psychiatr Rehabil J* 2019;42(2):169.
